# Supplementary material for: Modeling the Complete Dynamics of the SARS-CoV-2 Pandemic of Germany and Its Federal States Using Multiple Levels of Data
Source: Viruses. 2025 Jul 14;17(7):981. doi: 10.3390/v17070981 (PMC12297911; doi:10.3390/v17070981)
Supplement: Supplementary file 1 [file viruses-17-00981-s001.zip › viruses-3604628-supplementary.pdf]

viruses

MDPI

Supplementary Material

Contents

Supplementary Material A. Explanation of model structure, compartments and parameters 2

Section S0. General description of the model structure 2

Table S1. Description of model compartments. 4

Table S2. Basic epidemiologic parameters. 6

Table S3. Probabilities of becoming hospitalized, critical (ICU requirement) or dead...................... 7

Table S4. Parameters of residual infectivity and starting dates of new virus variants. 8

Table S5. Contact matrix. 9

Table S6. Parameters used to define the input layer of our IO-NLDS. 10

Table S7 Estimated values of residual infectivity of new variant vv+1 relative to the preceding variant vv. 10

Section S8. Groups of SARS-CoV-2 variants considered in the model 11

Table S9. Probability of infection pIS for different constellations of immune stati and infecting variants. 12

Supplementary Material B. Model equations 13

Susceptible compartments.. 13

Sub-model of infected compartments 13

Recovered compartments 15

The recovered compartment is devided into three sub-compartments to mimic immune-waning.15

Vaccinated compartments: 15

Supplementary Material C. Input layer 16

Supplementary Material D. Output Layer 17

Supplementary Material E. Parameter Estimation methods 19

Supplementary Material F. Justification of fixed parameter values from the literature 22

Supplementary Material G. Estimation of unreported Cases (Dark Figure) until November 15, 2022 23

Supplementary Material H. Agreement of model and data of case numbers by age group per federal state 27

Supplementary Material I. Agreement of model and data of severe disease courses per federal state 43

Supplementary Material J. Age-group specific dynamics of infectivity for Germany 48

Supplementary Material K. Age-stratified hospitalization transition rates across German federal states for different SARS-CoV-2 variants. 52

Supplementary Material L. Age-stratified ICU admission transition rates across German federal states for different SARS-CoV-2 variants. 53

Supplementary Material M. Prediction of the dynamics of immune states for the federal states 54

Supplementary Material N. Modelled age-stratified immune state dynamics of infected subjects and vaccination coverage across German federal states during the COVID-19 pandemic.......................... 70

Supplementary Material O. Development of age-groups over the course of the pandemic 88

References.. 88

Supplementary Material A. Explanation of model structure, compartments and parameters

Section S0. General description of the model structure

We here present a detailed description of our model structure. A schematic overview of the model can be found at **Figures 1&2** of the main paper. We neglect influences of the birth rate on the age-compartment sizes, as well as changes due to aging or non-COVID-19 related mortality. This is justified by a relative stability of the age groups over the relatively short duration of the pandemic (see **Supplementary Material O**).

1. Infectible subjects are not currently but could become infected. This compartment comprises
    a. Non-vaccinated subjects without previous infections (*Sc*): We assume that this applies to 100% of the population at the beginning of the epidemic.
    b. Vaccinated compartment. Vaccination effect is on one hand delayed and on the other hand subjected to waning. We model this by four sub-compartments with different susceptibility to infection:
        i. People from *S* directly switch to Vac_0 according to a daily vaccination rate imposed on the model and given by data. Vac_0 can be infected with the same probability as *S* mirroring a delayed vaccination effect.
        ii. The following three vaccinated compartments Vac_1, Vac_2 and Vac_3 represent different states of immune waning after vaccination with a transition rate of r_w = 1/73 days^{-1}. Degree of protection of the different *Vac* compartments is assumed variant dependent (see below). Booster vaccinations refresh immunity by returning Vac_2 and Vac_3 back to Vac_1.
    c. The compartment of recovered subjects *R* is structured into three sub-compartments R_1, R_2 and R_3 again mirroring immune-waning.
        i. Waning is modelled in the same way as for the respective *Vac* compartments. Likewise, additional vaccinations return R_2 and R_3 to R_1.
        ii. The probabilities of infection p_{IS} of compartments depend on both, the virus variant of the previous infection event and the actually infecting variant. Respective relations are estimated and discussed in Supplemental tables S7-S9, providing details on considered groups of virus variants and the dependency on the previous infecting variant.
        iii. In case of repeated infections, we only consider the immune memory of the last infection event, i.e. we do not consider immune memories of earlier infection events.

.

Table S1. Description of model compartments.

We describe the compartments of the model and their epidemiological meanings. We also provide brief justifications of the model structure.

**Com-part-ment**	**Sub-Compart-ments**	**Description**
S		Susceptible subjects
E		Latent stage of infection (infected but not infectious), transits to I_1 with rate r_3.
Vac	Vac_0	Vaccination state 0: Freshly vaccinated assuming the same epidemiologic properties as S due to a delay in vaccinations becoming effective.
	Vac_1	Vaccinated state 1 representing the highest immunization state after vaccination. Subjects still can get infected but with low probability. Infected subjects derived from Vac_1 are assumed to be immune against critical disease courses or death.
	Vac_2	Vaccinated state 2 representing waned immunization compared to Vac_1 assuming a waning rate r_w. Subjects in this compartment can get infected with a higher probability than Vac_1, but still, they do not develop critical disease courses or die due to infection. Subjects could return to Vac_1 after receiving a booster vaccination.
	Vac_3	Subjects in Vac_2 transit to Vac_3 with waning rate r_w. In Vac_3 they can either become infected or return to Vac_1 after booster vaccination. The probability of infection is higher than for Vac_2, infected subjects may become critical or die with lower probability than unvaccinated subjects.
I	I_1	Infected (and infectious) state 1, transits to I_2 with rate r_5.
	I_2	Infected (and infectious) state 2, can either die, i.e., transits to D with probability p_{death} and rate r_5 or transits to I_3 with probability $(1 - p_{death})$ and rate r_5. I_2 can become hospitalized (hospital ward), i.e. contribute to N_1 state in dependence on age, infecting virus variant and immune status. Subjects can also become critical contributing to the compartment of intensive care unit (ICU) admissions C. For simplification, in order to avoid modelling disease courses, we assume no efflux from I_2 towards C and N compartments, i.e. the latter compartments just count the number of hospital ward respectively ICU admissions. For the same reason, we do not consider transitions between the compartments N, C and D.
	I_3	Infected (and infectious) state 3, transits to I_4 with rate r_5.
	I_4	Infected (and infectious) state 4, transits to the compartment of recovered subjects R with rate r_5.
C	C_1	Critical disease state 1, not infectious, transits to C_2 with rate r_7. We like to remark that multiple critical compartments are required to model data of bed occupancies.
	C_2	Critical disease state 2, transits to C_3 with rate r_7.
	C_3	Critical disease state 3, transits to R with rate r_7.
H	H_1	Patients at hospital ward state 1, not infectious, transits to N_2 with rate r_9.
	H_2	Hospital ward state 2, transits to N_3 with rate r_9.
	H_3	Hospital ward state 3, transits to R with rate r_9.
R	R_1	Recovered state 1, can either become infected, or transits to R_2 with waning rate r_w. The probability of being infected is low. Infected subjects originating from this state neither develop critical states nor die from a new SARS-CoV-2 infection.
	R_2	Recovered state 2, can either become infected, transits to R_3 with waning rate r_w or return to R_1 through vaccination. The probability of being infected is higher than that of R_1. Infected subjects originating from this state neither develop critical states nor die from a new SARS-CoV-2 infection.

R_3	Recovered state 3, can either become infected or return to R_1 through vaccination. The probability of becoming infected is higher than that of R_2. Infected subjects originating from this state can become critical or die from a new SARS-CoV-2 infection but with lower probability than immune-naïve subjects.
D	Dead (absorbing state)

All parameters of the model are detailed in Tables S2-S4. To ensure comparability, parameter names have been retained from the previous version of our model. Consequently, gaps exist in the numbering of transition rates, as some rates have been omitted in the current version. While most parameters are estimated separately for each federal state, variability between states is penalized, as outlined in Supplement Material D. Parameters are estimated at different levels dependent on their nature. Parameters defining the residual infectivity of individuals infected with a new virus variant compared to the previous variant are estimated at the national level for Germany as a whole. In contrast, the initial dates and initial numbers of new virus variants are estimated individually for each federal state, reflecting regional differences in the introduction and spread of the variant.

Table S2. Basic epidemiologic parameters.

We present names, units and descriptions of all model parameters. We also provide parameter values derived from the literature. Transition rates can be interpreted as reciprocal transition times. Dependency of parameters on other covariates such as age or virus variant are mentioned. §: Further details on parameter settings and estimations are provided in Supplementary Material B, Supplementary Material E and Supplementary Material F.

**Parameter**	**Unit**	**Description**	**Determination**	**Dependencies**
influx	Subjects/ Day	Early initial influx into compartment *E* due to newly emerging virus variants, assumed for the first 3 weeks.	Estimated	
$rinflux^{age}$	-	Relative initial influx of infected subjects into age categories	Estimated for Germany, Proportional for federal states	Age-specific
r_1	Day⁻¹	Basic infection rate	Estimated	
m_c	-	Contact matrix (coefficients of relative contact frequencies between age groups)	Fixed, based on [1]	
$p_{IS}^{(age,vv,is)}$	-	Probability of infection, based on immune status *is*	Fixed	Variant-, age- and immune status-specific
r_3^{vv}	Day⁻¹	Transit rate for compartment *E* (1/latent time)	Fixed to 1/3 for pre-Omicron variants, fixed to 1/2 for omicron variants §, [2–6]	Variant-specific
r_5	Day⁻¹	Transit rate between infected sub-compartments	Fixed to 6/5 §	
r_6	Day⁻¹	Transit rate of becoming critical (ICU requirement)	Fixed to 1/5 §, [2,7–9]	
r_7	Day⁻¹	Transit rate between critical states (ICU)	Fixed to 3/17 §,[2,10–12]	
r_8	Day⁻¹	Transit rate to death of patients	Fixed to 1/8 §,[7,13,14]	
r_9	Day⁻¹	Transit rate of becoming hospitalized	Estimated	
r_v	Day⁻¹	Transit rate until vaccination is becoming effective	Set to 1/14 for age<80 years, 1/21 otherwise§	
r_w	Day⁻¹	Transit rate of immunity waning	Set to 1/73§, reflecting the order of magnitude of published waning dynamics [15–20].	
DRD_{mean}		Average delay in death reporting	Fixed to $\frac{1}{r_5}+\frac{1}{r_8}$, see Supplement Materials C, “Modelling of reported deaths”	

Table S3. Probabilities of becoming hospitalized, critical (ICU requirement) or dead

We present names, units and descriptions of the model parameters. We also provide the way of determination of parameters. Dependency of parameters on other covariates such as age or virus variant are mentioned. §: Further details on parameter settings and estimations are provided in Supplementary Material B, Supplementary Material E and Supplementary Material F.

Parameter	Unit	Description	Determination	Dependencies
$p_{death,WT}$	-	Mortality for WT infection	Estimated	Age-specific
rp_{death}^{Wan}		Reduction in mortality due to Vac_3, R_3 immune status	Set to 0.4	
rp_{death}^{vv}		Mortality factor for virus variant *vv*	Estimated	Age- and variant-specific
p_{death}	–	Mortality	Calculated by multiplying $p_{death,WT}$, rp_{death}^{vv} and rp_{death}^{Wan} if relevant, see (B3)	Variant-, age- and immune status-specific
$p_{crit,WT}$	-	Probability of becoming critical for WT	Estimated	Age-specific
rp_{crit}^{Wan}		Reduction in probability of becoming critical due to Vac_3, R_3 immune status	Set to 0.4	
rp_{crit}^{vv}		Probability factor of becoming critical for virus variant *vv*	Estimated	Age- and variant-specific
p_{crit}	–	Probability of becoming critical	Calculated by multiplying $p_{crit,WT}$, rp_{crit}^{vv} and rp_{crit}^{Wan} if relevant (B3)	Variant-, age- and immune status-specific
$p_{hosp,WT}$	-	Probability of becoming hospitalized for WT	Estimated	Age-specific
rp_{hosp}^{Wan}		Reduction in becoming hospitalized due to Vac_3, R_3 immune status	Set to 0.4	
rp_{hosp}^{vv}		Probability factor for becoming hospitalized for virus variant *vv*	Estimated	Age- and variant-specific
p_{hosp}	-	Probability of becoming hospitalized	Calculated by multiplying $p_{hosp,WT}$, rp_{hosp}^{vv} and rp_{hosp}^{Wan} if relevant (B3)	Variant-, age- and immune status-specific

Table S4. Parameters of residual infectivity and starting dates of new virus variants.

In this table, we present names, units and descriptions of the model parameters related to residual infectivity and starting dates of new virus variants. In parameter ini^{vv} *vv* considers virus variants ordered according to the respective times of appearance: 1=WT, 2=α, 3=δ, 4=BA1, 5=BA2, 6=BA5, 7 = BA.2.75/ BQ.1, 8 = XBB, 9 = BA.2.86 and 10 = KP.3. Virus variant grouping is described in **Table S8,** further details on parameter settings and estimations are provided throughout **Supplementary Material A and B.**

Parameter	Unit	Description	Determination	Dependencies
$Tini^{vv}$	-	Starting dates of new virus variants *vv*. Strictly monotone time sequence	Estimated	Variant-specific
ini^{vv}	Subjects	Starting (initial) number of infected subjects of virus variant *vv* at time $Tini_{vv}$		
$ri^{vv1+1,vv}$	-	Residual Infectivity of new variant *vv+1* relative to the preceding one *vv**		
$ri^{vv,WT}$		Residual infectivity of variant *vv* compared to WT	Calculated $\prod_{vvx=1}^{vv} ri^{vvx+1,vvx}$	

Contact matrix: For the pre-epidemic era, Mossong et al. [1] established an age-dependent contact matrix for Germany, which provides the average number of physical contacts that each member of an age group (row) has reported with members of the same or another age group (column). We used the pooled values of the age-group 60-75 of Mossong et al. for our age group 60-79 and the values for age-group 75+ for our age group 80+. We symmetrized this matrix by averaging it with its transpose and normalized it to resemble values previously estimated for the average contact situation during the epidemic. Resulting values are shown in Table S5. Contact matrix elements are multiplied by the dynamical infecting rate b_1 to calculate actual infection rates.

Table S5. Contact matrix.

The following baseline contact matrix was used in our modelling and is based on Mossong et al. [1]. True infection rates between age-groups are calculated by multiplication with the actual time dependent infection rate b_1.

Age categories	1-14 years	15-34 years	35-59 years	60-79 years	80+ years
1-14 years	0.566	0.207	0.247	0.0695	0.0378
15-34 years	0.207	0.822	0.42	0.107	0.0559
35-59 years	0.247	0.42	0.652	0.224	0.109
60-79 years	0.0695	0.107	0.224	0.319	0.08
80+ years	0.0378	0.0559	0.1088	0.08	0.0945

Table S6. Parameters used to define the input layer of our IO-NLDS.

Parameters are used to empirically describe changing NPIs, contact behavior, testing policies, vaccination intensities and other external factors relevant for the course of the epidemic in Germany. Related input functions constitute the input layer of our IO-NLDS model. Details can be found in Supplementary Material **C.**

**Parameter**	**Unit**	**Description**	**Source**	**Remarks**
N_{tr}	-	Number of time points of changes of contact intensities	Empirically defined	13 intensifications, 15 relaxations according to BIC
$b_{tr,j}$, $j = 1,\ldots, N_{tr}$	-	Residual infectivity of subjects in the time interval [tr, tr + 1]	Estimated	-
Tr_j, $j = 1,\ldots, N_{tr}$	Days	Time points of changes	Estimated or fixed	Strictly monotone sequence
Del_{tr}	Days	Delay of activation of NPIs	Fixed	2 days

Table S7 Estimated values of residual infectivity of new variant vv+1 relative to the preceding variant vv.

$ri^{\alpha,WT}$	$ri^{\delta,\alpha}$	$ri^{BA1,\delta}$	$ri^{BA1,BA2}$	$ri^{BA2,BA5}$	$ri^{BA5,BA2.75/BQ1}$	$ri^{BA2.75/BQ1,XBB}$	$ri^{XBB,BA2.86}$	$ri^{BA2.86,KP3}$
1.36	1.71	1.37	1.35	1.10	1.104	1.107	1.32	1.23

Section S8. Groups of SARS-CoV-2 variants considered in the model

Classification of SARS-CoV-2 virus variant and estimation of their previous frequencies in Germany were performed as follows. Initially, sequence data from Germany were obtained, including information on genetic lineages, sampling dates, and frequencies [21]. Variants were mapped to the ECDC's VirusVariants classification available at https://www.ecdc.europa.eu/sites/default/files/documents/PathogenVariant_public_mappings.csv" and https://www.ecdc.europa.eu/en/covid-19/variants-concern, which categorizes variants based on their sub-lineage definition and impact on immunity and transmissibility, also accounting for the corresponding WHO classification. In cases where a lineage was not included in the ECDC classification, it was labeled as "not in ECDC" and not further considered. For dates before December 10, 2020, the lineage model was set to "WT", as no significant variants were circulating. Between December 10, 2020, and October 20, 2021, lineages not classified as "Alpha", "Beta", "Gamma", or "Delta" were also assigned "WT". Omicron variants BA.1 and B.1.1.529 were combined into “BA.1", BA.4 and BA.5 were combined in a single BA.4+5 group, BQ.1 and BA.2.75 were combined into " BA.2.75/ BQ.1", and XBB variants including XBB.1.5 variants were combined into a "XBB*" group. To create a smoothed time series, cubic regression spline with a beta response distribution in the framework of generalized additive model implemented in the R package *mgcv* 1.9.1 were used.

Figure S1: Frequency distribution for Germany of considered groups of virus variant lineages showing **A)** ECDC-reported frequencies, and **B)** re-grouped smoothed and aggregated raw data as used in the model.

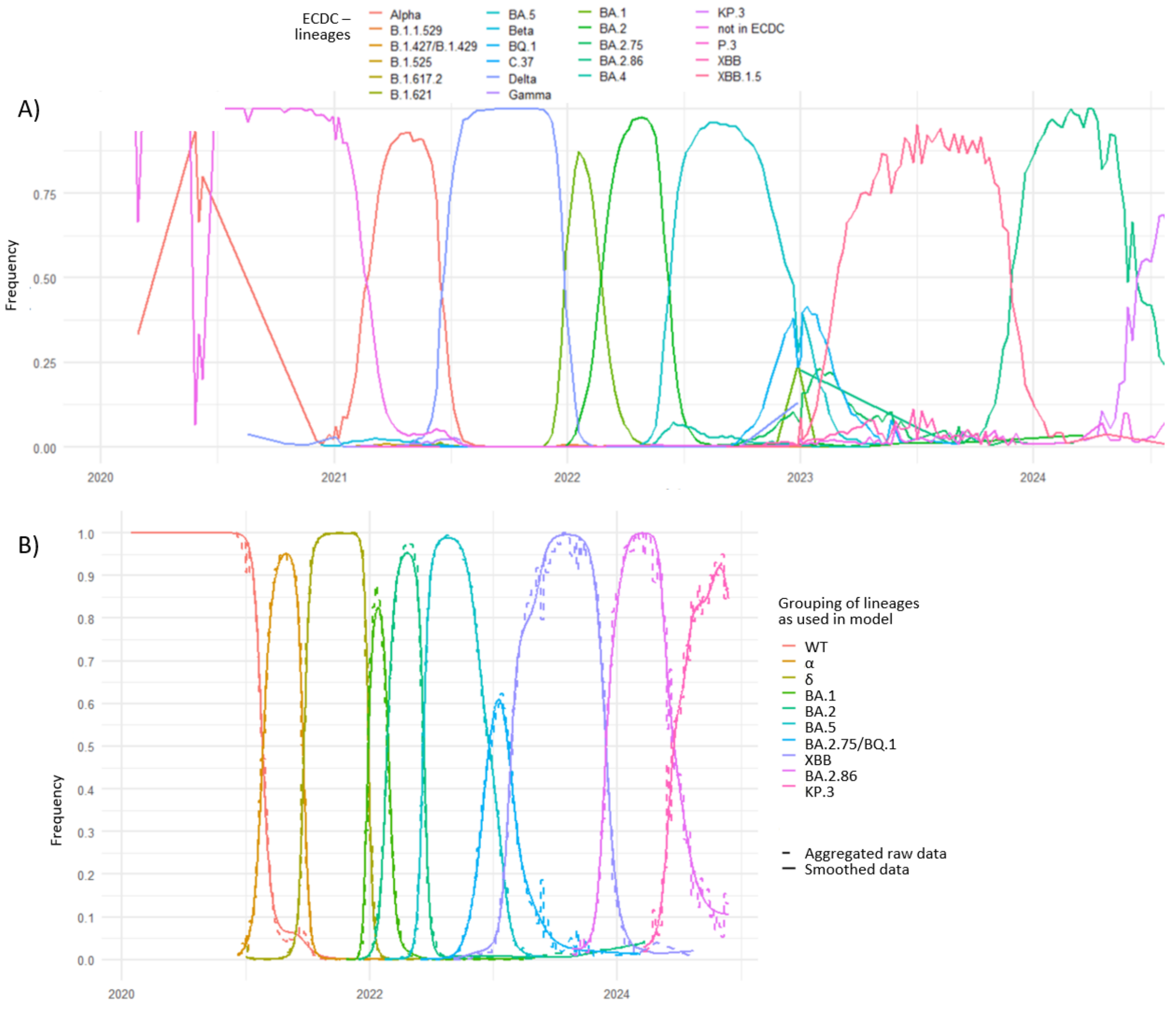

Table S9. Probability of infection p_{IS} for different constellations of immune stati and infecting variants.

In our model, probability of infection depends on immune status, previous infecting variant (for recovered subjects) and current infecting variants. Parameters for *S* are fitted. Other values are fixed according to estimations from the literature [15–20]. In particular, Altarawneh et al. describe omicron reinfection cases [18]; BA1 and BA2 infecting rates are studied in [19,20].

Immune status	Previous immuni-zation event	Infecting SARS-CoV-2 variant									
		WT	alpha	delta	BA1	BA2	BA4+5	BA.2.75/ BQ.1	XBB	BA.2.86	KP.3
Vac$_1$		0.1	0.1	0.15	0.25	0.25	0.5	0.6	0.6	0.6	0.5
Vac$_2$		0.1	0.1	0.15	0.25	0.25	0.5	0.7	0.7	0.7	0.5
Vac$_3$		0.25	0.25	0.35	0.5	0.5	0.5	0.75	0.75	0.75	0.5
R$_1$	WT	0.01	0.01	0.05	0.3	0.3	0.4	0.6	0.6	0.6	0.4
	Alpha	0.01	0.01	0.05	0.3	0.3	0.4	0.4	0.6	0.6	0.4
	Delta	0.01	0.01	0.01	0.3	0.3	0.5	0.4	0.4	0.5	0.4
	BA1	-	-	-	0.1	0.1	0.2	0.5	0.4	0.4	0.4
	BA2	-	-	-	0.15	0.1	0.2	0.2	0.5	0.4	0.4
	BA4+5	-	-	-	0.2	0.2	0.1	0.2	0.2	0.5	0.4
	BA.2.75/ BQ.1	-	-	-	-	0.2	0.2	0.1	0.2	0.2	0.2
	XBB	-	-	-	-	-	0.2	0.2	0.1	0.2	0.1
	BA.2.86	-	-	-	-	-	-	0.2	0.2	0.1	0.1
	KP.3	-	-	-	-	-	-	-	0.2	0.2	0.1
R$_2$	WT	0.05	0.05	0.05	0.4	0.4	0.5	0.7	0.7	0.7	0.5
	Alpha	0.05	0.05	0.05	0.4	0.4	0.5	0.5	0.7	0.7	0.5
	Delta	0.05	0.05	0.05	0.4	0.4	0.5	0.5	0.5	0.65	0.5
	BA1	-	-	-	0.15	0.2	0.3	0.5	0.5	0.5	0.5
	BA2	-	-	-	0.25	0.15	0.3	0.3	0.5	0.5	0.5
	BA4+5	-	-	-	0.3	0.3	0.15	0.3	0.3	0.5	0.5
	BA.2.75/ BQ.1	-	-	-	-	0.3	0.3	0.15	0.3	0.3	0.3
	XBB	-	-	-	-	-	0.3	0.3	0.15	0.3	0.2
	BA.2.86	-	-	-	-	-	-	0.3	0.3	0.15	0.15
	KP.3	-	-	-	-	-	-	-	0.3	0.3	0.15
R$_3$	WT	0.1	0.1	0.1	0.45	0.45	0.5	0.75	0.75	0.75	0.5
	Alpha	0.1	0.1	0.1	0.45	0.45	0.5	0.5	0.75	0.75	0.5
	Delta	0.1	0.1	0.1	0.45	0.45	0.5	0.5	0.7	0.75	0.5
	BA1	-	-	-	0.2	0.3	0.35	0.5	0.5	0.5	0.5
	BA2	-	-	-	0.4	0.2	0.35	0.35	0.5	0.5	0.5
	BA4+5				0.35	0.35	0.2	0.35	0.35	0.5	0.5
	BA.2.75/ BQ.1	-	-	-	-	0.35	0.2	0.2	0.35	0.35	0.35
	XBB	-	-	-	-	-	0.35	0.2	0.2	0.35	0.3
	BA.2.86	-	-	-	-	-	-	0.35	0.2	0.2	0.2
	KP.3	-	-	-	-	-	-	-	0.2	0.35	0.3

Supplementary Material B. Model equations

In this section, we present all equations of our model.

Susceptible compartments

The compartment of uninfected susceptible subjects (*S*) is age dependent, we distinguish different age groups by introducing respective sub-models (*SM*). We do not consider births, non-COVID-19 related deaths or transitions between age-compartments due to aging. This is justified by the relative stability of the considered age-groups during the pandemic (see **Supplementary Material O**). Consequently, there are only effluxes from the susceptible compartments due to (1) new infections (influx), (2) transition from S to E compartment due to infection and (3) additional effluxes due to vaccination (transition to vaccinated compartments):

$$\frac{\Delta S^{SM}}{\Delta t} = -influx \cdot rinflux^{age} - Efflux^{S} - rate_{vac,S}, \quad \text{(B1)}$$

Where $influx$ represents infections acquired outside of the regional entity (Germany, federal states) considered, e.g. during abroad stays. We assume, that this is relevant during the three weeks after 4th March, 2020. This effectively reduces the number of uninfected susceptible cases and introduces the virus. Parameter $rinflux^{age}$ describes the age-specific relative contribution to these virus introductions.

Efflux^{S} represents infections arising from the susceptible uninfected compartment and is defined later. “rate” represents vaccinations of susceptible uninfected subjects (see below).

Sub-model of infected compartments

Parameters for infection dynamics depend on age, infecting virus variant and immune status. Thus, we consider sub-models (*SM*) which correspond to respective parameter configurations (*age*, *vv*, *is*). These sub-models have the same structure (see below). We consider the compartments of exposed (*E*), infected (*I*), hospitalized (*N*), critically ill (*C*) and dead subjects (*D*) and frist order fluxes between these compartments. Some of these compartments are sub-divided into structurally similar sub-compartments to mimic time delays. Hospitalized cases, critical cases as well as deaths are all directly derived from I_2, the second sub-compartment of infected subjects, and only deaths are considered as hart efflux from the system for simplicity.

$$
\begin{aligned}
&\frac{\Delta E^{SM}}{\Delta t} = Infect_E^{SM} - r_3 \cdot E^{SM}\\
&\frac{\Delta I_1^{SM}}{\Delta t} = r_3 \cdot E^{SM} - r_5 \cdot I_1^{SM}\\
&\frac{\Delta I_2^{SM}}{\Delta t} = r_5 \cdot I_1^{SM} - r_5 \cdot (1 - p_{death}) \cdot I_2^{SM} - p_{death} \cdot r_8 \cdot I_2^{SM}\\
&\frac{\Delta I_3^{SM}}{\Delta t} = r_5 \cdot (1 - p_{death}) \cdot I_2^{SM} - r_5 \cdot I_3^{SM}\\
&\frac{\Delta I_4^{SM}}{\Delta t} = r_5 \cdot I_3^{SM} - r_5 \cdot I_4^{SM}\\
&\frac{\Delta C_1^{SM}}{\Delta t} = r_{crit} \cdot I_2^{SM} - r_7 \cdot C_1^{SM}\\
&\frac{\Delta C_2^{SM}}{\Delta t} = r_7 \cdot C_1^{SM} - r_7 \cdot C_2^{SM}\\
&\frac{\Delta C_3^{SM}}{\Delta t} = r_7 \cdot C_2^{SM} - r_7 \cdot C_3^{SM}\\
&\frac{\Delta R_{ICU}^{SM}}{\Delta t} = r_7 \cdot C_3^{SM}\\
&\frac{\Delta H_1^{SM}}{\Delta t} = r_{hosp} \cdot I_2^{SM} - r_9 \cdot H_1^{SM}\\
&\frac{\Delta H_2^{SM}}{\Delta t} = r_9 \cdot H_1^{SM} - r_9 \cdot H_2^{SM}\\
&\frac{\Delta H_3^{SM}}{\Delta t} = r_9 \cdot H_2^{SM} - r_9 \cdot H_3^{SM}\\
&\frac{\Delta R_{Hosp}^{SM}}{\Delta t} = r_9 \cdot H_3^{SM}\\
&\frac{\Delta D^{SM}}{\Delta t} = p_{death} \cdot r_5 \cdot I_2^{SM}
\end{aligned}
\quad , \qquad \text{(B2)}
$$

The parameters r_{hosp} r_{crit} and p_{death} are calculated according to the following distinction of cases, depending on the infecting virus variant *vv* and the immune-status, with immune status naïve and *Vac₀* having similar properties regarding susceptibility to severe disease courses:

$$
r_{hosp} = \begin{cases} r_{hosp,WT}, & vv\ WT\ , immune\ status\ naive\ or\ Vac_0 \\ r_{hosp,WT} \cdot rr_{hosp,vv} \cdot rr_{hosp,Vac}, & Vac_{1,2}, R_{1,2}\ compartments, vv \\ r_{hosp,WT} \cdot rr_{hosp,vv} \cdot rr_{hosp,Wan}, & Vac_3, R_3\ compartments, vv \end{cases}
$$

$$
p_{death} = \begin{cases} p_{death,WT}, & vv\ WT\ , immune\ status\ naive\ or\ Vac_0 \\ p_{death,WT} \cdot rp_{death,vv} \cdot rp_{death,Vac}, & Vac_{1,2}, R_{1,2}\ compartments, vv \\ p_{death,WT} \cdot rp_{death,vv} \cdot rp_{death,Wan}, & Vac_3, R_3\ compartments, vv \end{cases} \quad ,
$$

$$
r_{crit} = \begin{cases} r_{crit,WT}, & vv\ WT\ , immune\ status\ naive\ or\ Vac_0 \\ r_{crit,WT} \cdot rr_{crit,vv} \cdot rr_{crit,Vac}, & Vac_{1,2}, R_{1,2}\ compartments, vv \\ r_{crit,WT} \cdot rr_{crit,vv} \cdot rr_{crit,Wan}, & Vac_3, R_3\ compartments\ , vv \end{cases}
$$

(B3)

Parameter r_3 depends on the immune-status (see **Supplemental Table S2**). Parameter r_7 is age-dependent. R_{Hosp}^{SM} and R_{ICU}^{SM} are only required to model total bed-occupancies, i.e. they represent no real fluxes of subjects.

$Infect_E^{SM}$ is the sum of infection influxes from all infecting compartments of a given virus variant:

$$
Infect_E^{SM} \equiv Infect_E^{(age,vv,is)} = r_1 \cdot \sum_{Z \in IS^{-1}(is)} \left(b_1^{age}(t) \cdot ri_{vv,WT} \cdot p_{is}(age, is, vv) \cdot \sum_{i=1}^{4} \sum_{isx} \sum_{agex=1}^{5} mat^{agex,age} \cdot I_i^{(agex,vv,isx)} \cdot \frac{Z^{(age,is)}}{Sc_{init}^{age}} \right), \qquad \text{(B4)}
$$

where Z is the sum of infectible compartments, contributing to a certain immune-status *is* (see **Figure 2** and **Table 1** of the main document). Reverse mapping IS^{-1} assigns to each immune status a set of potentially infectible compartments whose effluxes contribute to the respective immune status (see Table 1 of the main document). In detail,

$$Z^{(age,is)} = \begin{cases} S^{age} + Vac_0^{age}, if \quad is = Naive \\ Vac_1^{age} + Vac_2^{age} + \left(\sum_{vvx} R_1^{(agex,vvx,is)} + R_2^{(agex,vvx,is)}\right), if \quad is = RVac_{1,2}\,, \\ Vac_3^{age} + \sum_{vvx} R_3^{(agex,vxv,is)}\,, if \quad is = RVac_3 \end{cases} \quad \text{(B5)}$$

Matrix *mat* represents the contact matrix of our five age-groups. Subscript "*x*" corresponds to the variant which resulted in the most recent infection event of a subject, see Table S5.

The infection rate is calculated as a product of the following terms:

- A basic state-specific infection rate r_1 of the WT variant
- a dynamical infection rate $b_1^{age}(t)$, depending on age and external factors contributing to contacts such as changing NPI or social behaviour.
- ri_{WT}^{vv}, infectivity of the current variant *vv* relative to WT, *vv* is element of the set {Alpha, Delta, BA1, BA2, BA5, BA.2.75 with BQ.1, XBB, BA.2.86 and KP.3}.
- $\{mat_c^{agex,age}\}$ is the contact matrix of infecting subjects of age-group "age" with infected sub-groups of age "age$_x$". Five age groups are considered, see Table S5.
- Probability of infection of an infectible subject p_{is} (see Tables S7 and S8), which depends on age, *vv* and immune status: $p_{IS}^{(age,vv,is)}$.

Let us denote by $Efflux^Z$ the efflux from an infectible compartment Z to compartment E, then, the considerations above yield:

$$Efflux^Z = r_1 \cdot b_1^{age}(t) \cdot \sum_{i=1}^{4} \sum_{isx} \sum_{agex=1}^{5} \sum_{vvx} \left(ri^{vvx,WT} \cdot p_{IS}^{(agex,vvx,isx)} \cdot m_c^{agex,age} \cdot I_i^{(agex,vvx,isx)} \cdot \frac{Z}{Sc_{init}^{age}} \right), \quad \text{(B6)}$$

Recovered compartments

The recovered compartment is devided into three sub-compartments to mimic immune-waning.

$$\begin{aligned} \frac{\Delta R_1^{SM}}{\Delta t} &= r_5 \cdot I_4^{SM} - Efflux^{R_1^{SM}} + rate_{boost,R2} + rate_{boost,R3} - r_w \cdot R_1^{SM} \\ \frac{\Delta R_2^{SM}}{\Delta t} &= r_w \cdot R_1^{SM} - r_w \cdot R_2^{SM} - rate_{boost,R2} - Efflux^{R_2^{SM}} \\ \frac{\Delta R_3^{SM}}{\Delta t} &= r_w \cdot R_2^{SM} - rate_{boost,R3} - Efflux^{R_3^{SM}} \end{aligned} \quad , \quad \text{(B7)}$$

$rate_{boost,Y}$ is the daily boosting rate of recovered subjects in sub-compartment Y∈{R2, R3}. The daily overall boosting rate $rate_{boost}$ is available from RKI (https://github.com/robert-koch-institut/COVID-19-Impfungen_in_Deutschland). We assume, that $rate_{boost,Y}$ is proportional to the fraction of Y among all compartments eligible for booster vaccinations Z∈{ R2, R3, Vac2, Vac3} at the respective day *t*:

$$rate_{boost,Y}(t) = rate_{boost}(t) \cdot \frac{Y(t)}{\sum Z(t)}. \quad \text{(B8)}$$

The parameter r_w describes the waning of the immune protection.

Vaccinated compartments:

In analogy to the recovered compartments, the dynamics of vaccinated compartments are described as follows, again considering divisions into sub-compartments to mimic waning:

$$\begin{aligned}
\frac{\Delta Vac_0^{age}}{\Delta t} &= rate_{vac,s} - r_v \cdot Vac_0^{age} - Efflux^{Vac_0^{age}} \\
\frac{\Delta Vac_1^{age}}{\Delta t} &= r_v \cdot Vac_0^{age} - r_w \cdot Vac_1^{age} - Efflux^{Vac_1^{age}} + rate_{boost,Vac2} + rate_{boost,Vac3} \\
\frac{\Delta Vac_2^{age}}{\Delta t} &= r_w \cdot Vac_1^{age} - r_w \cdot Vac_2^{age} - Efflux^{Vac_2^{age}} - rate_{boost,Vac2} \\
\frac{\Delta Vac_2^{age}}{\Delta t} &= r_w \cdot Vac_2^{age} - r_w \cdot Vac_3^{age} - Efflux^{Vac_3^{age}} - rate_{boost,Vac3}
\end{aligned} \quad . \qquad (B10)$$

$rate_{vac,S}$ is a daily vaccination rate of *S*. In complete analogy to the boostering rate, the daily rate of first vaccinations $rate_{vac}$ is available from the German Robert-Koch institute (https://github.com/robert-koch-institut/COVID-19-Impfungen_in_Deutschland). We again assume that the $rate_{vac,Sc^{age}}$ is proportional to the fraction of respective age compartment of *S*:

$$rate_{vac,S^{age}}(t) = rate_{vac}(t) \cdot \frac{S^{age}(t)}{\sum_{age_x} S^{age_x}}. \qquad (B11)$$

The parameters r_v and r_w describe different waning parameters. We assume that r_v is relatively large compared to r_w since the protection against infection is known to be waning more rapidly compared to the protection against severe disease.

Supplementary Material C. Input layer

The input layer of our IO-NLDS is designed to describe external effects on the epidemic dynamics such as non-pharmaceutical interventions (NPI), vaccination campaigns, entry of new variants, changes in behavior, seasonal effects, larger outbreaks or factors affecting our data models such as changes in testing policy. These impacts are abbreviated as NPI/contact behavior in the following. Since these factors act differently on the epidemic dynamics or the data models, we typically model these effects phenomenologically by assuming time-dependent parameters such as time-dependent infection rates.

We assume that new variants *vv* appear instantaneously at certain dates $Tini^{vv}$ by assuming a certain percentage of subjects in *E* and the four sub-compartments of *I* as infected with the new variant, i.e. these subjects receive the new variant label. These new cases are proportionally distributed over these compartments:

$$\begin{aligned}
E^{(age,vvn,is)}(Tini_{vvn}) &= ini^{vv} \cdot E^{(age,vv,is)} / Tot_{inf}^{vv}(Tini^{vv}) \qquad &(C1) \\
I_i^{(age,vv,is)} &= ini^{vv} \cdot I_i^{(age,vv,is)} / Tot_{inf}^{vv}(Tini^{vv}) \qquad &, i = 1, \dots, 4
\end{aligned}$$

Where ini^{vvn} is the number of introduced infections by the new variant *vvn*, and:
$Tot_{inf}^{vv}(T) = \sum_{isx} \sum_{agex=1}^{5} \left(E^{(agex,vv,isx)}(T) + \sum_{i=1}^{4} I_i^{(agex,vv,isx)}(T) \right)$ is the total number of subjects infected with the previous variant *vv*. Time points of introduction of new variants $Tini^{vv}$ are estimated specific for the federal states and Germany.

<u>*Residual infectivity $b_1(t)$:*</u> Similarly to our previous model [22], we introduce the residual infectivity function $b_1(t)$, which changes according to NPI/contact behavior modifications. This is modelled by a linear increase (in case of relaxation) or decrease (in case of tightening of NPI) within a fixed time interval Del_{tr} of two days. Otherwise, $b_1(t)$ is constant. We denote $\{T_{tr,s}\}_{s=1}^{N_{tr}}$ as the time points with changes in non-pharmaceutical interventions with N_{tr} representing the total number of time points with assumed changes. We collected dates of changing non-pharmaceutical intervention measures for

Germany based on government decisions as well as events with impact on epidemiological dynamics such as holidays and outbreaks. Thus, $b_1(t)$ is a step function defined as follows:

$$b_1(t) = \begin{cases} b_{tr,s-1}, & t \in [T_{tr,s-1} + Del_{tr}, T_{tr,s}] \\ \frac{b_{tr,s-1}}{Del_{tr}} \cdot (Del_{tr} - t + T_{tr,s}) + \frac{b_{tr,s}}{Del_{tr}} \cdot (t - T_{tr,s}), & t \in [T_{tr,s}, T_{tr,s} + Del_{tr}] \\ b_{tr,s}, & t \in [T_{tr,s} + Del_{tr}, T_{tr,s+1}] \end{cases} , \quad \text{(C2)}$$

The time point t = 0 of our model corresponds to March 3rd, 2020. We assume that $b_1(t)$ is state- and age-specific. The time points $\{T_{tr,s}\}_{s=1}^{N_{tr}}$ are state-specific, while the number of time points N_{tr} is assumed constant across states to avoid over-fitting.

Supplementary Material D. Output Layer

We here describe, how the state parameters of the hidden SECIR model are linked to observational data via the output layer of our IO-NLDS. These links are based on our developed data models also addressing data issues such as delayed or incomplete reporting.

Modelling of daily reported infection numbers I_{rep}: It needs to be considered that reported infection numbers do not reflect actual infection numbers because reports are both, delayed and incomplete. To account for these issues, we related the efflux from our E compartments to the number of reported infections by multiplying with the reporting fraction rep_{fr} based on the dark figure estimates and by considering a reporting delay function D_{ln}:

$$\begin{aligned} I_{rep}^{SM}(T) &= \sum_{t=0}^{T} rep_{fr} \cdot r_3 \cdot I_1^{SM}(t) \cdot D_{ln}(T-t,\mu,\sigma) \\ \mathrm{DF} &= \frac{non-reported\ cases}{reported\ cases} \\ rep_{fr} &= \frac{reporteded\ cases}{reporteded\ cases + non-reporteded\ cases} = \frac{1}{1+DF} \end{aligned} \quad . \quad \text{(D1)}$$

The delay function $D_{ln}(T-t,\mu,\sigma)$ is a log-normal density with parameters μ and σ, i.e. we assume a delay between registered and reported cases by introducing an empirical distribution of the reporting delay. This distribution is determined based on data provided by the Robert-Koch-Institute (RKI) from the period April 27th, 2020 to November 13th, 2020. The parameters of this distribution are derived by minimizing the Kullback–Leibler divergence between the parametric representation and the empirical distribution. Results are displayed in **Figure D1**.

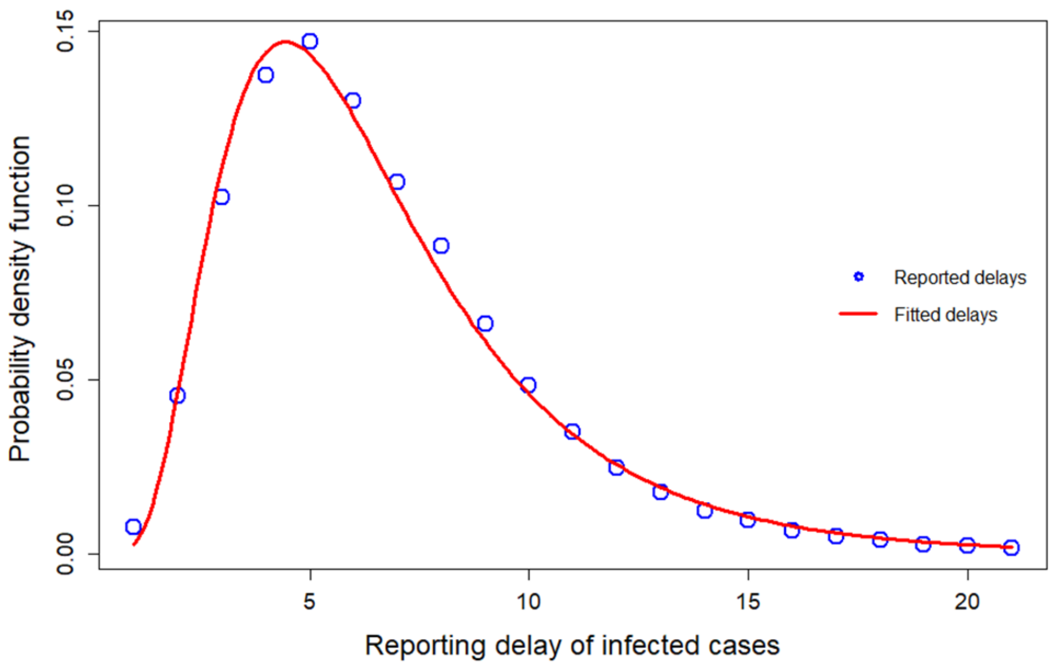

Figure D1. Approximation of the reporting delay by a log-normal distribution: We present the log-normal distribution best fitting the empirical distribution of reporting delays. Estimated parameters of the log-normal distribution are: μ = 1.77 days, σ = 0.531 days.

Available reported daily data I_{rep} are age-specific. Therefore, we sum equation (D1) across virus variants to match with the reported data, with I_M denoting the modelled infection numbers:

$$I_{rep}^{age}(T) = \sum_{allelex} \sum_{ux} \left(I_M^{(age,vvx,isx)}(T) \right). \qquad \text{(D2)}$$

Modelling of reported deaths: The RKI provides dates of positive testing of later deceased patients as “dates of deaths”. The average time lag between positive testing and death is $\frac{1}{r_5} + \frac{1}{r_8}$, because branching to death is possible after the second infected compartment I_2 in our model. Taking into account equation (D1), we approximate this data for every age category *age* at time point *T* by $I_{rep\_D}^{SM}\left(T - \frac{1}{r_5} - \frac{1}{r_8}\right)$ where $I_{rep\_D}^{SM}$ describes the number of infections from the respective submodel that are later reported to have died. Since the death reporting is coupled to the reporting of positive testing, we assume the same delay function D_{ln} as for the reported infection numbers. We assume no dark figure of death reports:

$$D_{rep}^{SM}(T) = I_{rep\_D}^{SM}\left(T - \frac{1}{r_5} - \frac{1}{r_8}\right) = \sum_{t=0}^{T-\frac{1}{r_5}-\frac{1}{r_8}} r_3 \cdot I_1^{SM}(t) \cdot D_{ln}\left(T - \frac{1}{r_5} - \frac{1}{r_8} - t, \mu, \sigma\right)$$
$$D_{rep}^{age}(T) = \sum_{vvx} \sum_{isx} \left(D_{rep}^{(age,vvx,isx)}(T) \right). \qquad \text{(D3)}$$

Dage_rep is compared with reported deaths of this age-group.

Modelling hospitalized cases: In analogy to the model of reported deaths, we assume:

$$H_M^{SM}(T) = I_{D,M}^{SM}\left(T - \frac{1}{r_5} - \frac{1}{r_9}\right) = I_{D,M}^{(age,vv,is)}\left(T - \frac{1}{r_5} - \frac{1}{r_9}\right) = \sum_{t=0}^{T-\frac{1}{r_5}-\frac{1}{r_9}} r_3 \cdot I_1^{SM}(t) \cdot D_{ln}\left(T - \frac{1}{r_5} - \frac{1}{r_9} - t, \mu, \sigma\right)$$
$$H_M^{age}(T) = \sum_{vvx} \sum_{isx} \left(H_M^{(age,vvx,isx)}(T) \right). \qquad \text{(D4)}$$

Hospitalized cases include ICU cases and cases on regular wards.

<u>*Modelling critical cases (ICU):*</u> We assume that correct and complete dates of ICU admission were available from the German Intensive Care Association (DIVI) at a daily scale, i.e. we directly linked these data to our compartment *C*.

<u>*Modelling variant frequencies:*</u> Daily relative variant frequencies of Germany are available online (https://www.rki.de/DE/Content/InfAZ/N/Neuartiges_Coronavirus/Daten/VOC_VOI_Tabelle.html, accessed 10th March, 2023). Since this data is based on the daily variant cases, the respective output layer is:

$$R^{vv}(T) = \frac{\sum_{isx}\sum_{agex=1}^{5}\left(I_M^{(agex,vv,isx)}(T)\right)}{\sum_{isx}\sum_{agex=1}^{5}\sum_{vvx}\left(I_M^{(agex,vvx,isx)}(T)\right)}. \quad (D5)$$

Supplementary Material E. Parameter Estimation methods

Free parameters of the model are determined by minimizing the negative log-likelihood function of observed data considering parameter priors and constrains, similarly to our previous works [22,23]. As explained in the main text, the prior constraining was checked for the fitting comparison and abandoned at the final estimation however. Thus, the likelihood can be written as follows:

$$nLL = nLL^{pri} + nLL^{resid} + Constr. \quad (E1)$$

The terms nLL_i^{pri} and nLL_i^{resid} correspond to prior/variance constraints of parameters and to the residual errors of the data as explained below in detail. The term *Constr* is a penalty term to keep values in eligible ranges or orders.

<u>*Parameter distributions and transformations:*</u> Most of the parameters are confined to certain ranges. During estimation (with possible prior constraints), we transform these parameters to the space of real numbers. We assume that these transformed values are normally distributed. To ensure this, parameters confined to a finite interval *(a,b)* are transformed by the *logit*-function. Parameters with positive values are transformed by a log-normal transformation. Thus,

$$\varphi_s \equiv h_s(\psi_s) = \begin{cases} e^{\psi_s}, & for\ parameters > 0 \\ a + (b-a)\cdot\frac{e^{\psi_s}}{1+e^{\psi_s}}, & for\ parameters\ within\ [a,b] \end{cases}, \quad s = 1, \dots, N_{par}, \quad (E2)$$

where φ_s is the *s*-th parameter and ψ_s is the respective transformed parameter and N_{par} is the total number of parameters to be estimated.
The negative likelihood contribution of the priors nLL^{pri} is defined as follows:

$$nLL^{pri} = \sum_{s=1}^{N_{par}}\left(\delta_s\cdot\frac{\left(\psi_s-\psi_s^{pri}\right)^2}{\omega_{pri,s}^2} + (1-\delta_s)\cdot\frac{\left(\psi_s-\bar{\psi}_s\right)^2}{\omega_{pri,s}^2}\right), \quad (E3)$$

where δ_s equals 1, if a prior is assumed for the *s*-th parameter and 0 otherwise. The prior information is represented by the best guess for ψ_s^{pri} and an uncertainty expressed as standard deviation of possible values $\omega_{pri,s}$. We assume that parameter estimates are random variables normally distributed around their respective prior values. Thus,

$$\psi_s \sim N(\psi_s^{pri}, \omega_{pri,s}) = N\left(h_s(\varphi_s^{pri})^{-1}, \omega_{pri,s}\right). \quad (E4)$$

Best values are provided at Table S2. Most of the parameters are constrained by their variance between states, except the parameters estimated for Germany, only. Residual infectivity of new variant *vv+1* relative to the preceding one *vv* are shown in **Table S7.** Correspondingly, $\bar{\psi}_s$ denotes an average value of estimates of ψ_s for 17 entities (16 federal states and Germany as a whole).

Uncertainties $\omega_{pri,k}$ are set to a value of two for these parameters, resulting in a relatively small constrain. An exception are the time points of assumed NPI changes Tr,j or time points of entry of new variants $Tini_{vv}$. For these parameters we set $\omega_{pri,k}$ = 6 days. These heuristic settings for $\omega_{pri,k}$ are based on an empirical tradeoff between avoidance of overfitting including implausible parameter values and good data fitting properties.

Penalizations: The following penalizations are applied:

1. Times of non-pharmaceutical interventions need to be well-spaced and monotonic:

$$Pen_T(s) = \begin{cases} \frac{1}{\left(0.1+0.05\cdot(T_{tr,s}-T_{tr,s-1})\right)^8}, & T_{tr,s} > T_{tr,s-1} \\ 10^8, & T_{tr,s} \le T_{tr,s-1} \end{cases}, \quad s = 2, \dots, N_{tr}. \quad \text{(E5)}$$

Figure E1 shows $Pen_T(s)$ for different $T_{tr,s} - T_{tr,s-1}$. The penalty is very large when this time difference is shorter than a week, effectively enforcing that changes in b_1 values at short time scales are avoided.

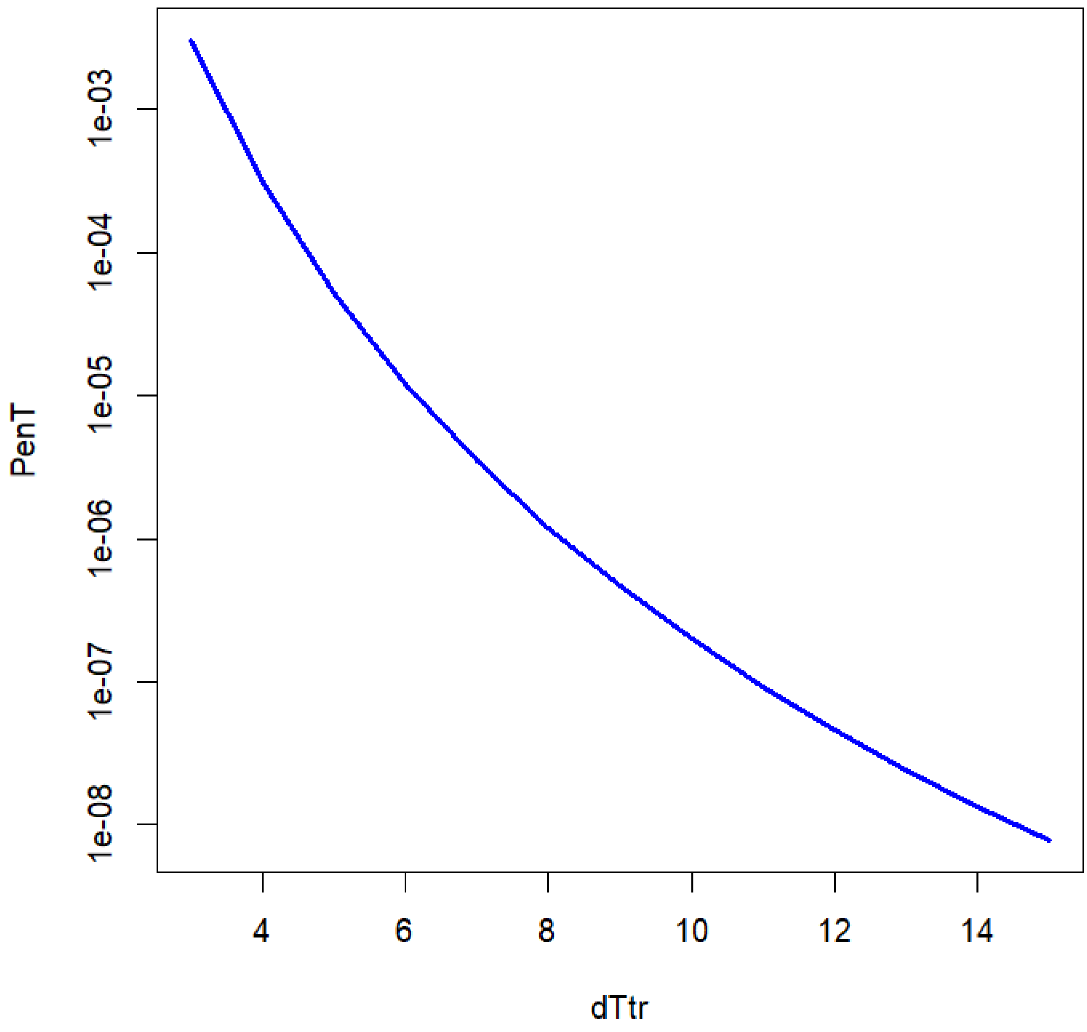

2. Conservation law: The sum of all model compartments is assumed to be constant and equal to *S(0)*. However, in case of very fast vaccination/boostering schedules, our difference equation approximation could theoretically result in negative compartment sizes. Since negative values were set to zero, this could result in violations of the conservation law. In order to avoid this behaviour, we impose the following penalty on the difference between the sum of compartment sizes at each time point T and the initial population, using factor 0.01 in order to have only little effect on the fitting of the reported data:

$$Pen_{Const}(T) = 0.01 \cdot \left(\sum_{agex=1}^{5}\left(Sc^{agex}(T) + \sum_{i=0}^{3} Vac_i^{agex}(T) + \sum_{isx}\left(E^{(agex,allex,ux)}(T) + \sum_{i=1}^{4} I_i^{(agex,allex,ux)}(T) + \sum_{i=1}^{3} R_i^{(agex,allex,ux)}(T) + D^{(agex,allex,ux)}(T)\right)\right) - \sum_{agex=1}^{5} Sc^{agex}(0)\right)^2. \quad \text{(E7)}$$

3. Penalization of parameter variability between federal states: In order to reduce overfitting of b_1, we penalized the between state variability of estimated time points of jumps. We set the weight of this constraint to 1/6 days, corresponding to a standard deviation of six days. We determined eight fixed change points (motivated by changes in NPIs) and 37 empirically identified change points. As in our previous model [22] we used Bayesian Information Criterion for optimal choice of the number of these change points.

$$Pen_{Var} = \sum_{j=1}^{N_{tr}} \sum_{agex=1}^{5}\left(Var_{States}(b_{tr,j}) + \frac{Var_{States}(Tr_j)}{36}\right), \quad \text{(E8)}$$

where $Var_{States}(x)$ is the variability of parameter x between federal states. In summary, the total penalty term reads as

$$Constr = \sum_{s=1}^{N_{tr}} Pen_T(s) + \sum_{T=0}^{T^{max}} Pen_{Const}(T) + Pen_{Var}. \quad \text{(E9)}$$

Residual errors of observed vs. predicted data: We fit data for daily reported cases, cumulative reported cases, deaths, cumulative deaths, regular ward and ICU occupation and variant frequency as explained in sub-section "output layer" and the methods section of the main text. The respective terms of the negative log-likelihood nll_i^{resid} correspond to the residual errors of these data. Thus,

$$nLL^{resid} = \sum_{Y\in\{I,D,C,N\}}\left(we_{Y,d} \cdot \sum_t \frac{\left(Y_{M,d}^{age}(t,\psi)^{tr_Y} - Y_{D,d}^{age}(t)^{tr_Y}\right)^2}{a_{Y,d}^2} + we_{Y,c} \cdot \sum_t \frac{\left(Y_{M,c}^{age}(t,\psi)^{tr_Y} - Y_{D,c}^{age}(t)^{tr_Y}\right)^2}{a_{Y,c}^2}\right) + we_{R_d^{vv}} \cdot \sum_t \sum_{vvx}\left(R_{M,d}^{vv}(t,\psi) - R_D^{vv}(t)(t)\right)^2 \cdot \quad \text{(E10)}$$

where *Y* represents the output layers. Subscript *M* denotes modelled quantities (see Fig. 1 in the main text), while *D* corresponds to the daily reported data. Subscript *d* means daily, while *c* means cumulative counts. The reported data is smoothed with a sliding window of seven days, as mentioned in Materials and Methods section of the main text. Empirically, we set weights $we_{Y,c}$ of the cumulative terms to 0.2 of those corresponding to the daily data, i.e. larger emphasize is placed on fitting daily data. The cumulative terms were introduced to avoid long-term biases, which would occur if fitting daily data only. Cumulative data for regular ward and ICU occupation were not fitted because only actual occupation numbers are of relevance, i.e., $we_{ICU,c} = we_{Hosp,c} = 0$. The weight $we_{R_d^{vv}}$ is chosen empirically to be 20,000 in order to make this term relevant for parameter estimations. This is required since the values of R^{vv} are always in between 0 and 1, i.e. they are relatively small compared to the other terms of equation (E10). All other weights $we_{Y,d}$ were set to 1. The parameter tr_Y corresponds to the power transformation used to compare model and data. In all model version, it is set to 0.5. It constitutes a trade-off between fitting precision of large and small numbers.

Likelihood optimization: We used a variant of the Hooke-Jeeves method [24] for likelihood maximization. This is a zero-order algorithm, which does not require calculation of derivatives of the fitness function to be optimized, which would be computationally expensive in our situation. In brief,

the method relies on iterated updates of the actual fitness function values and its arguments by comparisons with fitness values in the neighbourhood of the argument separately for all coordinates. Perturbation sizes at each dimension are adapted in dependence on the result of the previous step, i.e. a perturbation in the *s*-th dimension becomes larger if a better fitness value was found for this dimension in the previous step. Otherwise, it is reduced in the next step, provided that it does not drop below a specified lower limit. Algorithm stops if the last four steps did not provide a relative improvement of the fitness function of more than a specified tolerance parameter δ_{tol}. Residual errors $a_{x,d}$ and $a_{x,c}$ are estimated iteratively as well: After each iteration *K* of the Hook-Jeeves algorithms, they are updated as approximations of residual errors of the corresponding entities *Y*:

$$a_{Y,d,K} = \sqrt{\sum_{T=T_0}^{T_{max}} \frac{\left(Y_{M,d,K-1}^{age}(t,\psi_{K-1})^{tr_Y} - Y_{D,d}^{age}(t)^{tr_Y}\right)^2}{T_{max}-T_0}},$$
$$a_{Y,c,K} = \sqrt{\sum_{T=T_0}^{T_{max}} \frac{\left(Y_{M,c,K-1}^{age}(t,\psi_{K-1})^{tr_Y} - Y_{D,c}^{age}(t)^{tr_Y}\right)^2}{T_{max}-T_0}} \quad \text{(E11)}$$

Where the third subscript *K* respectively or *K-1* corresponds to the respective iteration step of the Hook-Jeeves algorithm.

Supplementary Material F. Justification of fixed parameter values from the literature

We here provide justifications of fixed parameter values. Details of parameter definitions and fitting can be found in Supplementary Material and E, respectively.

<u>*Fixed dates for updates of infectivity functions:*</u> We used a set of defined dates of possible changes in infectivity functions due to known changes in NPIs, outbreaks or other events. For the sake of parsimony, we still test, whether changes in infectivity at these time points are necessary, i.e. would significantly improve model fits. Likewise, we assume additional time points of changing infectivity to account e.g. for changing behavior of the population in response of the epidemiologic situation. These additional time steps are introduced if resulting in a significant improvement of model fit (BIC for the first 25 time points, beginning with the 26th time point, in order to optimize computational efficiency, time points at which residual error becomes larger than 150% of previous values). Assumed fixed time points of NPI change are derived from following information: The first three fixed time-points, tr_1 (March 10th, 2020), tr_2 (March 15th, 2020), and tr_3 (March 22nd, 2020) reflect German governmental interventions including regulation of the size of public events, travel restrictions, and contact restriction. Fixed time-points tr_6 (April 30th, 2020), tr_7 (May 7th, 2020), and tr_8 (May 21st, 2020) reflect German governmental interventions related to the step-wise relaxation of NPIs, in particular regarding leisure sports, contacts, and schools. Time point tr_{17} (November 2nd, 2020) reflects governmental NPIs in response to the German second wave, including restrictions of public life and social contacts, also referred to as "soft lockdown". Time point tr_{21} (December 16th, 2020) reflects tightening governmental NPIs in response to the continued increase of incidences in the framework of the German second wave, also referred as "hard lockdown", strongly limiting public and private contacts including school closures. Time point tr_{28} (February 23rd, 2021) reflects release of many governmental NPIs in response to the decline of the German second wave. Thereafter, we did not assume fixed dates but completely relied on our method of assuming new time steps based on improved model fit as described above.

<u>*Transition rate for compartment E (latent time) r_3:*</u> The transition rate r_3 for the compartment of exposed subjects is the inverse of the latent time, i.e. the time being infected but not yet infectious. We set this parameter to three days in accordance with previous reports [2]. For Omicron variants, we considered a shorter time reduced by one day as reported [25]. Further justification of this parameter is discussed in the following when considering the rate r_{4b}.

<u>*Transition rate of infected sub-compartments r_5:*</u> The transition rate r_5 of the four infected sub-compartments towards recovery are the inverse of the time being infectious equally distributed over the sub-compartments. Accordingly, we set r5 to 5/6 for each of the four infection compartments, which corresponds to an overall transit time of 3.3 days. [26].

<u>*Transition rate for critical state sub-compartments r_7:*</u> The transition rate r_7 of the three sub-compartments of the critical state is a third of the inverse of the average time survivors are treated at the intensive care unit (ICU). Accordingly, the value of r_7 was set to 3/17 per day. This value is informed by previous reports focusing on data of 35 to 79-year-old patients, the most frequent population at ICU [2,10–12].
<u>*Time of vaccination becoming effective:*</u> For individuals under the age of 80, we assumed that vaccination would take 14 days to become effective [27]. For older subjects, we assume 21 days in order to account for the effects of aging of the immune system [28] and the observation that antibodies increase more slowly in the elderly than in the young [29,30].
<u>*Waning of the immune protection:*</u> The rate of waning of immunity was assumed to happen in time steps. For each of them, we assume a duration of 2-3 months on average corresponding to a rate of approximately 1/73 per day according to characteristic waning patterns published elsewhere [15]. Parameters for the variant, vaccination and waning time-dependent levels of immunity follow those of previous reports [16] with adaptions required for fitting.

Supplementary Material G. Estimation of unreported Cases (Dark Figure) until November 15, 2022

We estimated the number of unreported cases per reported test-positive case (termed "Dark Figure" or DF, where DF=1 indicates one unreported case per reported case) using two distinct approaches for two different time periods, either before Nov 15, 2022, or after Nov 15, 2022.

For the period before November 15, 2022, we derived the DF from test positivity rates reported by the Robert-Koch-Institute [31]. Test positivity data was available weekly, either by federal state (for all ages combined, **Figure G1**) or by age group (for Germany only). To estimate age-specific test positivity by federal state, we applied the logit-scale differences between age groups observed at the national level to each state's overall positivity rate. We scaled these rates on the logit scale by centering them at 1.0 and multiplying them by a scale factor of 5.3. The latter was defined as three standard deviations of test positivity in all regions on the logit scale. This calculation yielded a dark figure estimate (**Figure G2**). The scaling and centering factors resulted from a calibration using multiple independent seropositivity studies [32] [33] [34], regarding SARS-CoV-2 infection-specific antibodies (Nucleocapsid-antibodies; **Figure G3**), including studies of blood donors and population-based surveys, which provided external validation of our estimates. This procedure resulted in an estimate of the time-, age-, and federal state-specific dark figures (**Figure G4**).

Figure G1-4. Estimation of dark figures from test positivity rates up to Nov 15, 2022. (G1) Weekly test positivity rates by federal state (thin lines) and Germany overall (thick green line) from 2020 to 2022. Y-axis shows test positivity on a logit scale with percentage labels. **(G2)** Dark number estimates from positivity rates after scaling and centering on the logit scale. **(G3)** Calibration of the scaling approach using independent seroprevalence studies measuring nucleocapsid antibodies (purple points/short lines) compared to the cumulative estimated infections incl. dark figure (colored lines) **(G4)**. Final dark figure estimates by federal state and age group over time. Values above 0 indicate underreporting of cases.

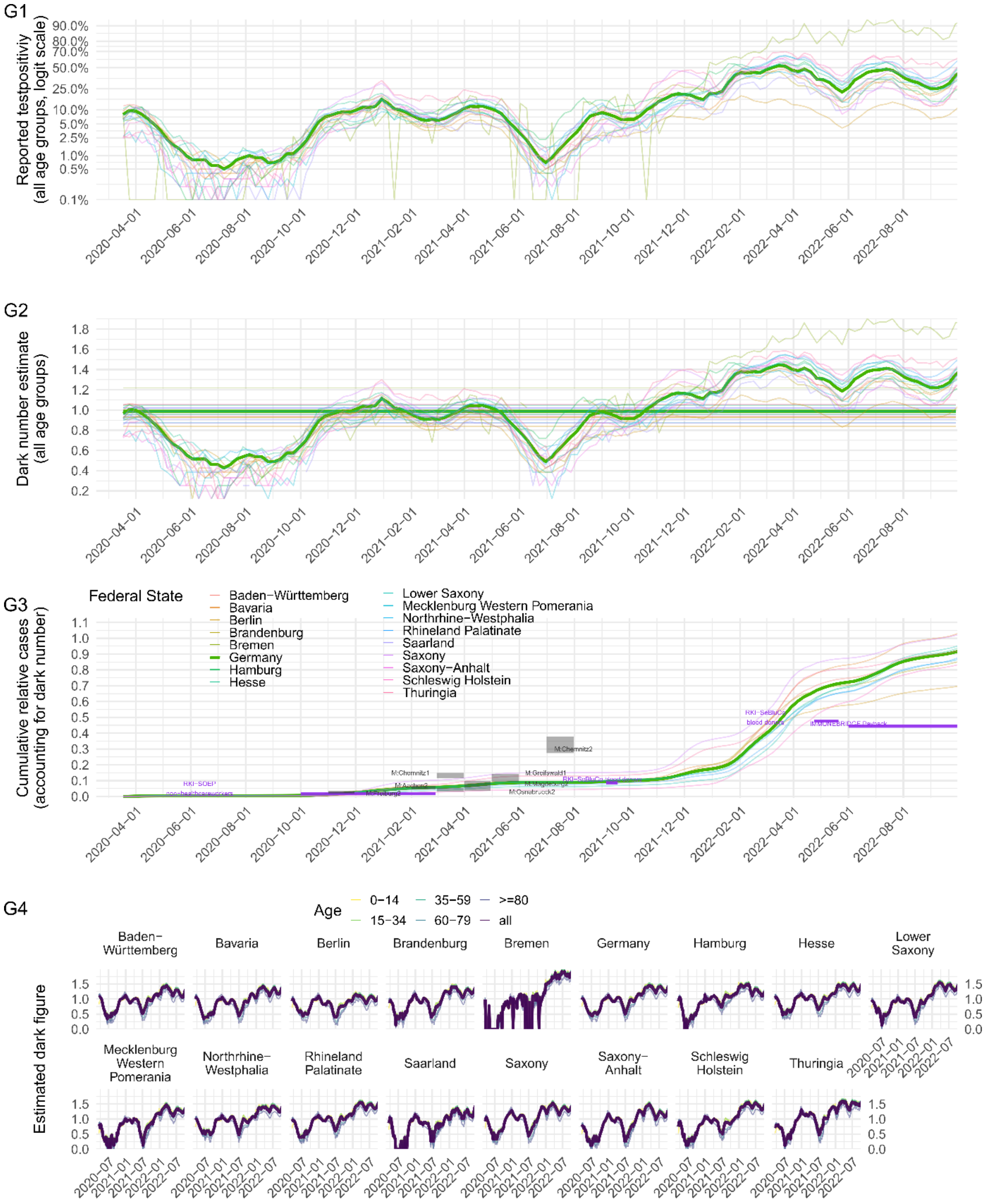

For the period after November 15, 2022, we utilized data from the SentiSurv study in Rhineland-Palatinate (https://www.unimedizin-mainz.de/sentisurv/), which provided systematic testing of a representative population sample starting at 2023-01-08 and provided resulting data on a website (https://www.unimedizin-mainz.de/SentiSurv-RLP/dashboard/index.html). We identified distinct phases in the pandemic response and testing patterns (in the non-age-stratified data):

Phase 1 (2022-11-16 to 2023-04-30) established the initial relationship between SentiSurv-detected and officially reported cases. *Phase 2* (2023-06-01 to 2023-08-15) captured a stabilized summer level with a dark ratio of approximately 75. Subsequent phases were defined to account for systematic changes in testing and reporting patterns: *Phase* 3 (2023-08-16 to 2023-08-31) as a transition period,

Phase 4 (2023-09-01 to 2024-02-14) covering the autumn/winter period, *Phase 5* (2024-02-14 to 2024-03-31) as another transition period, and *Phase 6* (2024-04-01 onwards) representing the 2024 spring/summer period **(G5)**.

Age-specific dark ratio information (SentiSurv/RKI incidence) was only available between October 2022 and March 2022, and the mean age-specific dark ratio of this period was considered representative of the entire period with SentiSurv data (2022-11-16 to 2024-03-34). Therefore, we multiplied the unstratified dark ratio time series by the age-specific means to obtain an estimate of the age-specific dark ratio time series. In doing so, we used the mapping of age categories between SentiSurv and RKI as shown in **(G6)**, with a mean dark figure ratio of the 0-14 year age group as 1.5 times that of the 15-44 year age group, as no data were available for persons under 25 years in SentiSurv. The resulting estimates for Germany are shown in **(G7)**. For individuals in the RKI age group 80+, we adjusted the estimated underreporting factor to 50% of the initial value, which significantly improved parameter estimates, particularly for the time-dependent infection rate (b_1). This adjustment is supported by the characteristics of the SentiSurv sample: participants in the oldest SentiSurv age group were still younger than the corresponding oldest RKI age group 80+ and, as voluntary participants, were likely to be more socially active than the population average for their age group.

Figure G5-7. Estimation of dark figures from test positivity rates after Nov 15, 2022. Panel **G5** shows the reported 7-day incidence per 100,000 inhabitants from SentiSurv and RKI data for Rhineland-Palatinate and Germany, alongside the calculated and phase-wise linear dark figure estimates. Panel **G6** displays age-stratified comparisons between SentiSurv and RKI incidences, showing both absolute values (top) and ratios (bottom) for matched age groups. Panel **G7** presents the estimated dark figures by age group in Germany, demonstrating varying levels of underreporting across different age cohorts. In the model, 50% of the dark figure of the 80+ age group was used.

G5

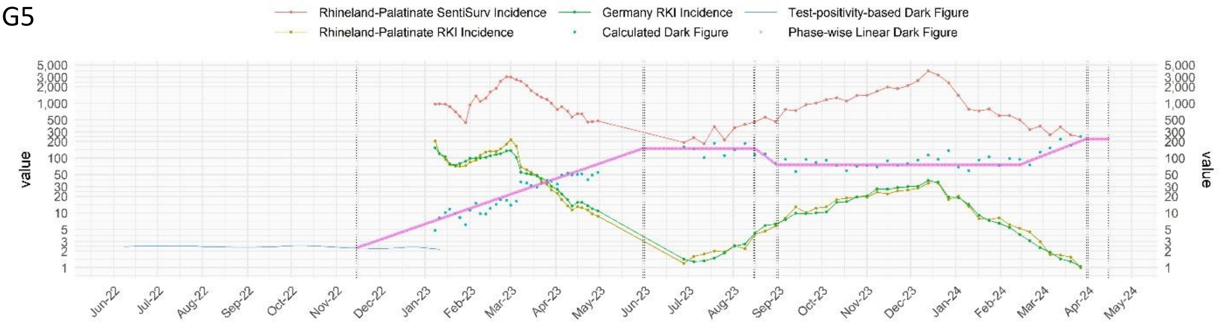

G6

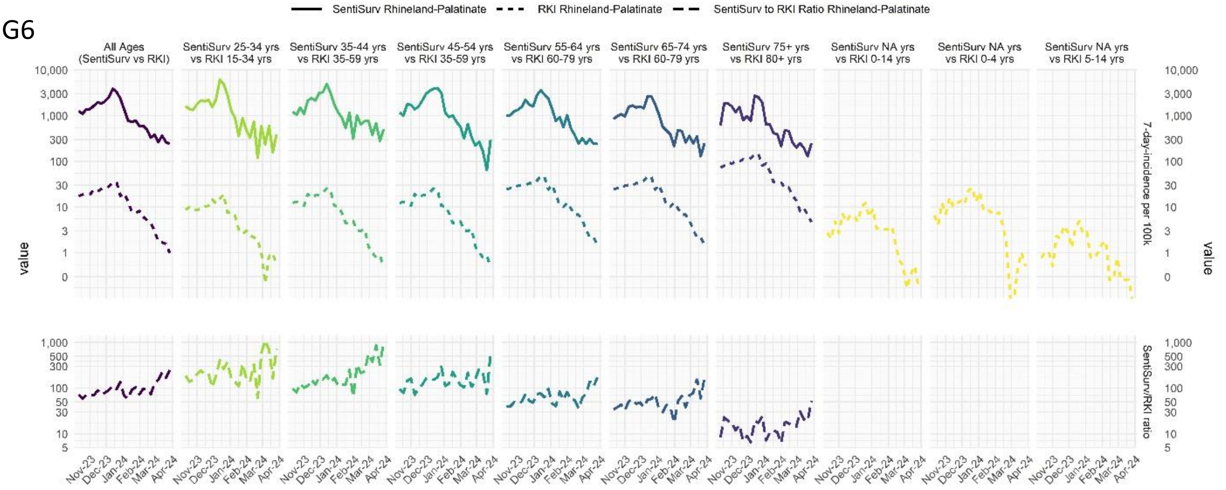

G7

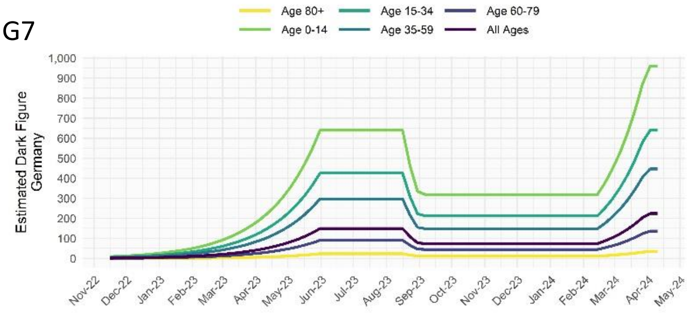

Supplementary Material H. Agreement of model and data of case numbers by age group per federal state

We present the agreement of model and data for case numbers by age group, shown on logarithmic scale: Bottom: Relative frequency of SARS-CoV-2 variants. Vertical dashed lines indicate start of new variant (group).

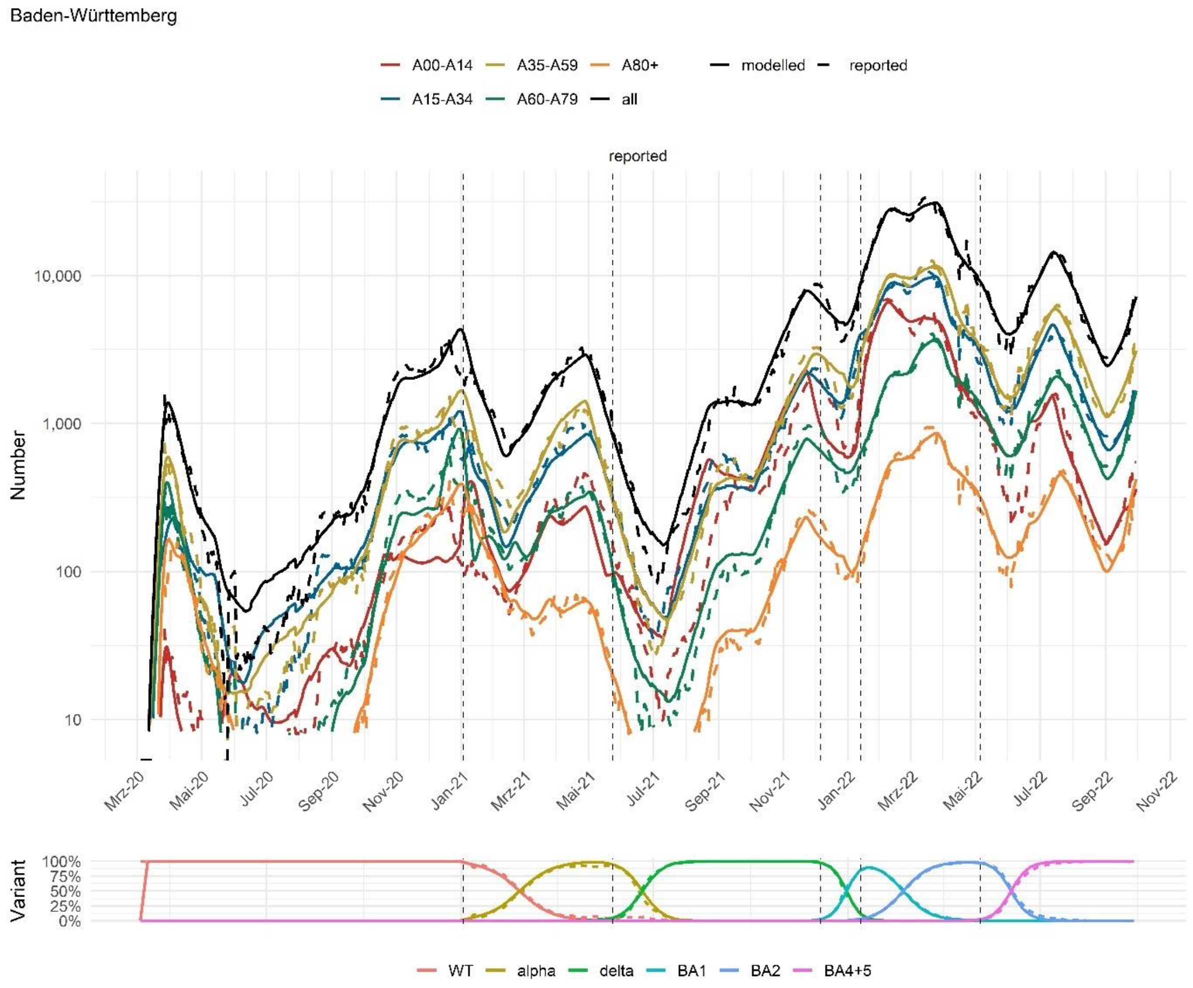

Bavaria

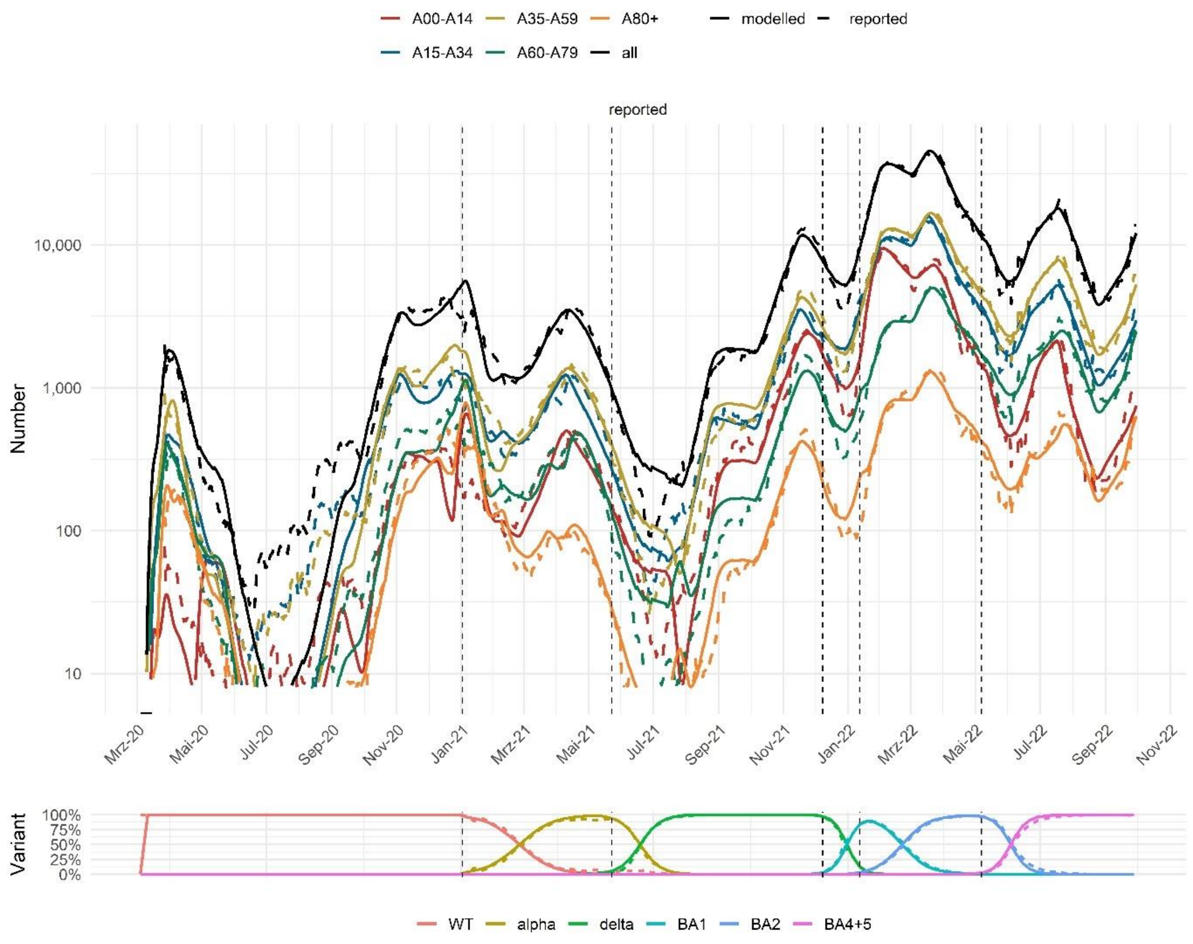
A00-A14
A35-A59
A80+
modelled
reported
A15-A34
A60-A79
all
reported
Number
10,000
1,000
100
10
Mrz-20
Mai-20
Jul-20
Sep-20
Nov-20
Jan-21
Mrz-21
Mai-21
Jul-21
Sep-21
Nov-21
Jan-22
Mrz-22
Mai-22
Jul-22
Sep-22
Nov-22
Variant
100%
75%
50%
25%
0%
WT
alpha
delta
BA1
BA2
BA4+5

Berlin

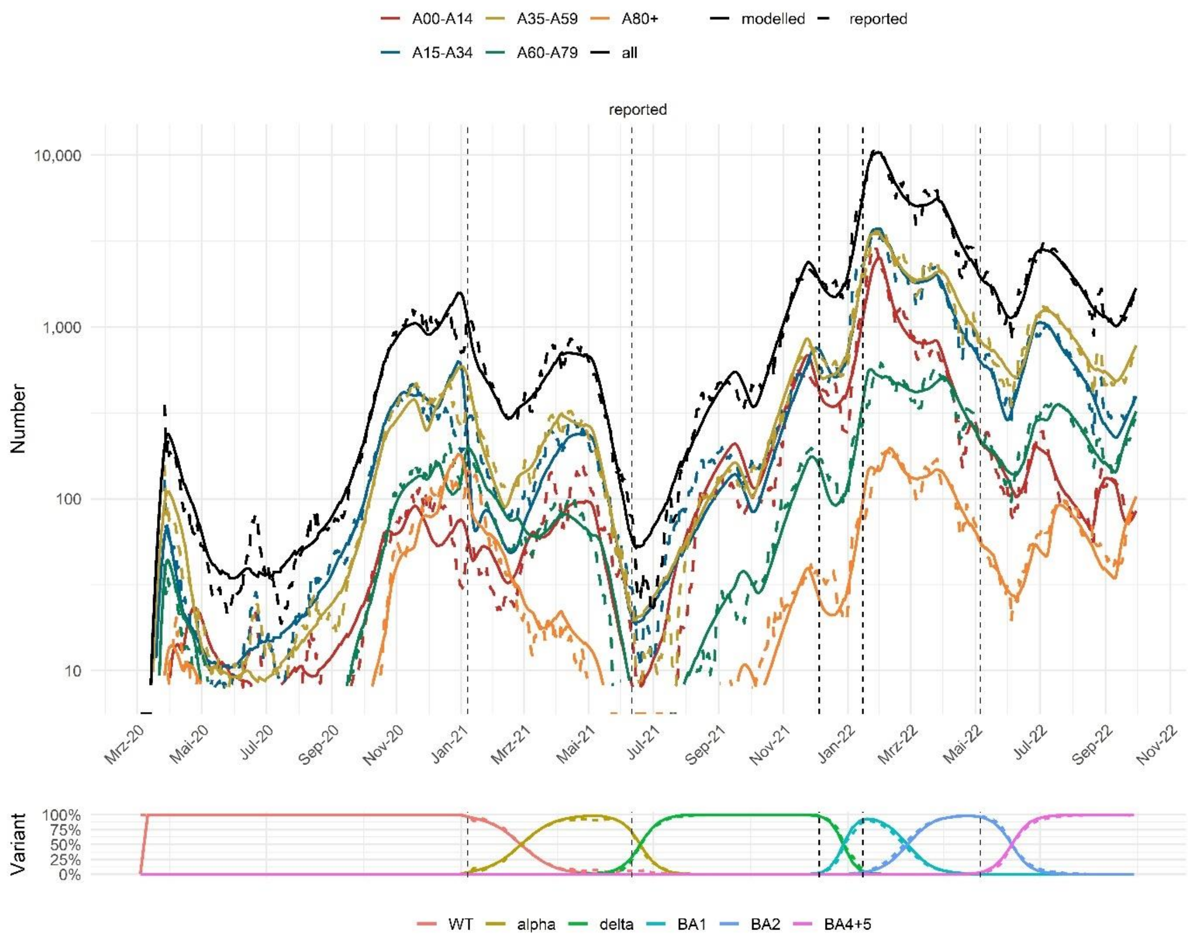
A00-A14
A35-A59
A80+
modelled
reported
A15-A34
A60-A79
all
reported
10,000
1,000
100
10
Number
Mrz-20
Mai-20
Jul-20
Sep-20
Nov-20
Jan-21
Mrz-21
Mai-21
Jul-21
Sep-21
Nov-21
Jan-22
Mrz-22
Mai-22
Jul-22
Sep-22
Nov-22
Variant
100%
75%
50%
25%
0%
WT
alpha
delta
BA1
BA2
BA4+5

Brandenburg

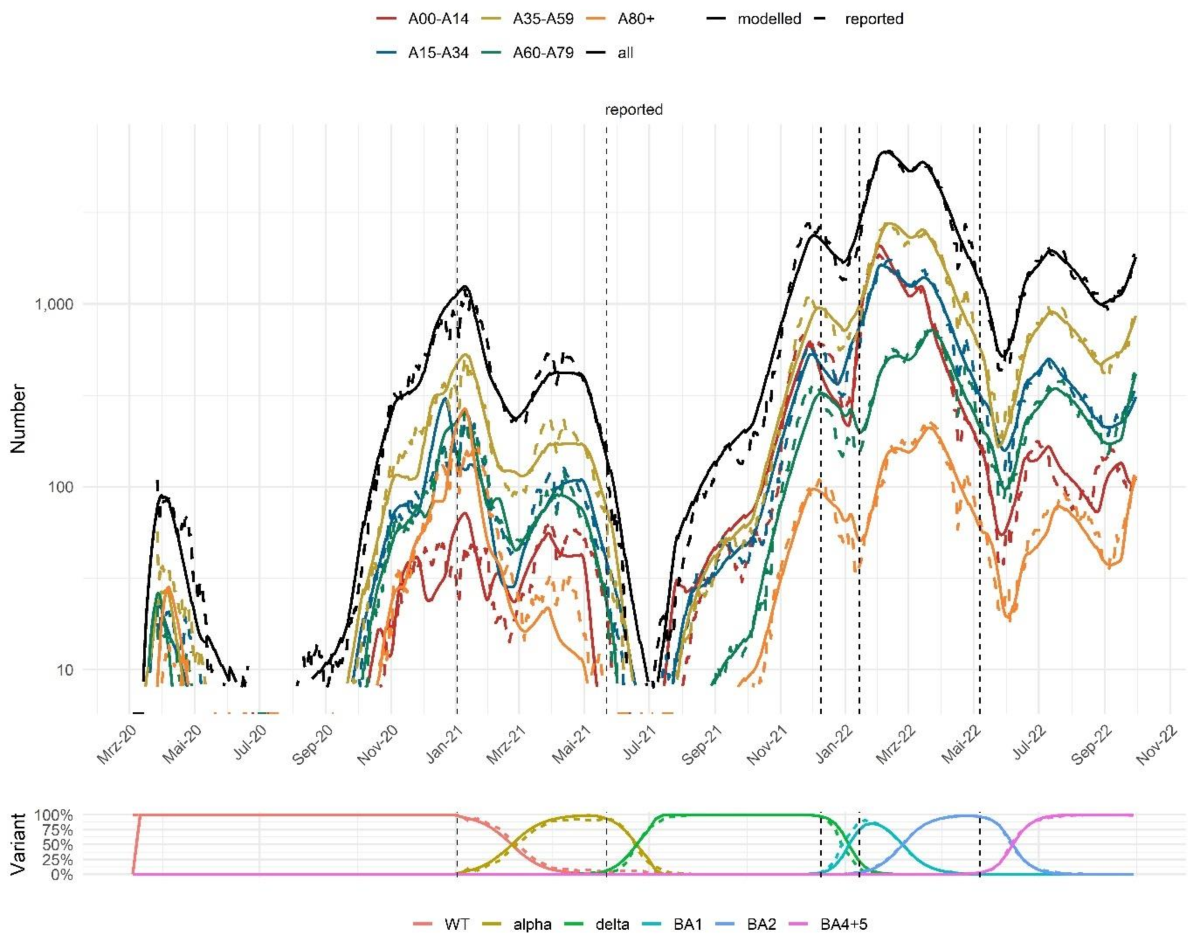

Bremen

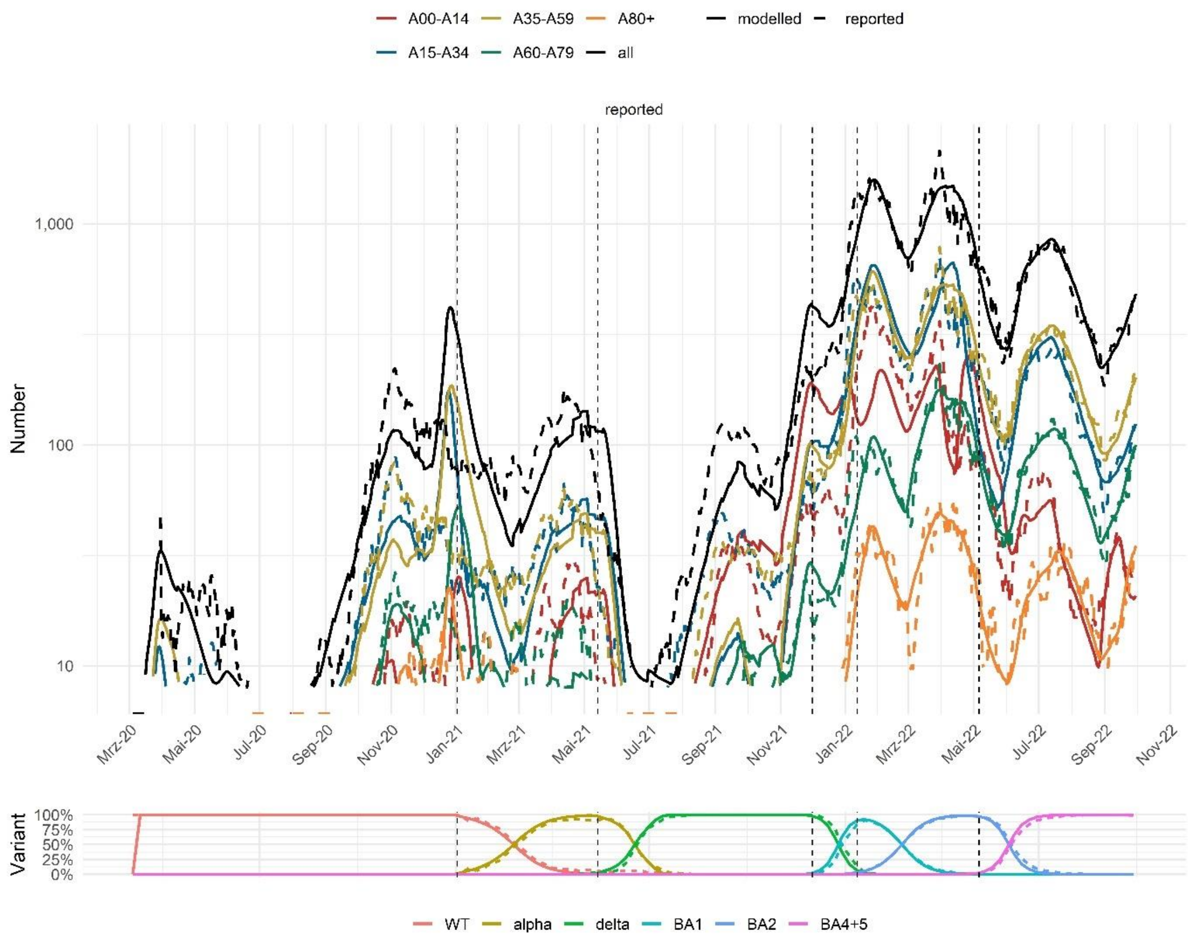
A00-A14
A35-A59
A80+
modelled
reported
A15-A34
A60-A79
all
reported
1,000
100
10
Number
Mrz-20
Mai-20
Jul-20
Sep-20
Nov-20
Jan-21
Mrz-21
Mai-21
Jul-21
Sep-21
Nov-21
Jan-22
Mrz-22
Mai-22
Jul-22
Sep-22
Nov-22
Variant
100%
75%
50%
25%
0%
WT
alpha
delta
BA1
BA2
BA4+5

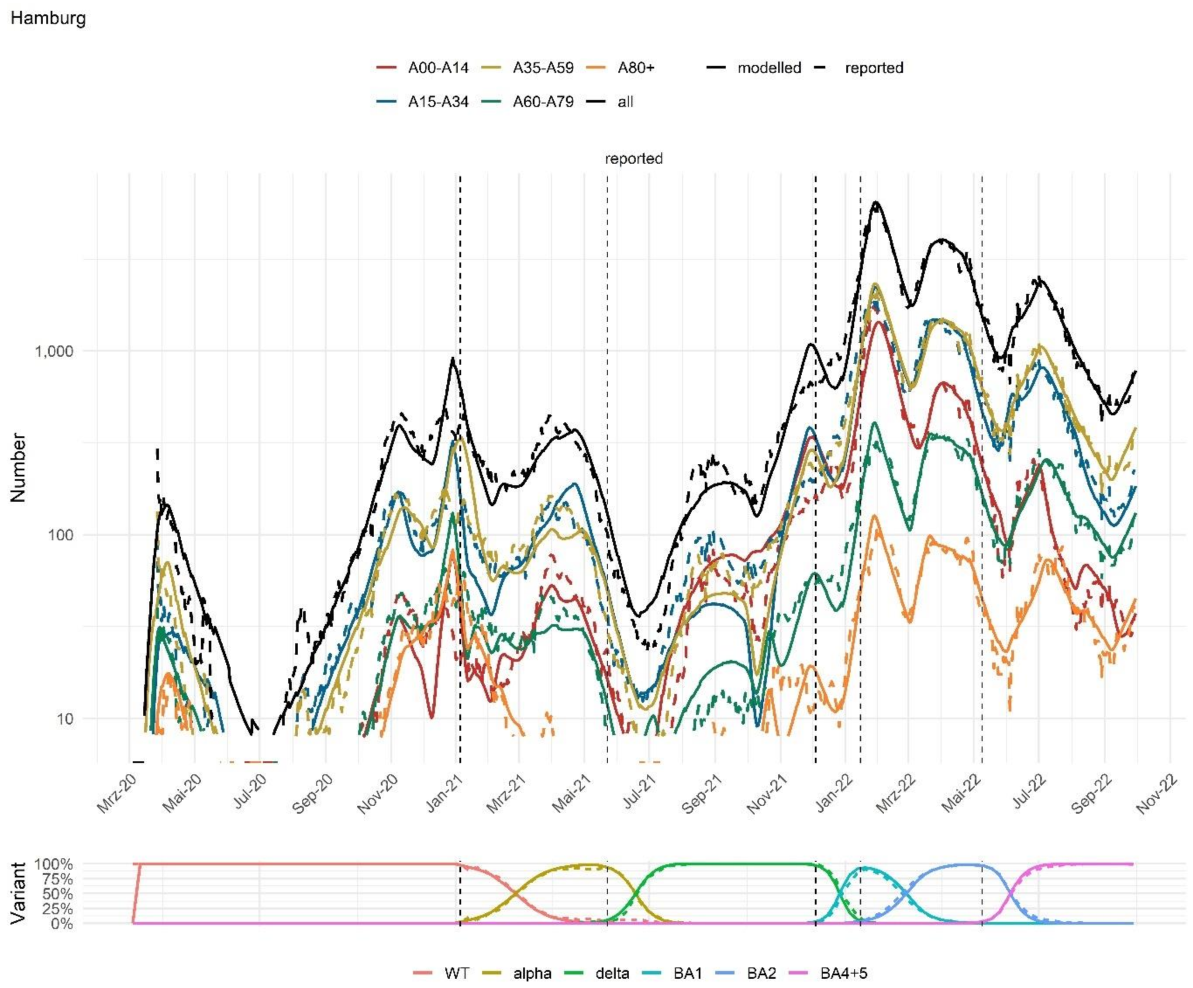
Hamburg
A00-A14
A35-A59
A80+
modelled
reported
A15-A34
A60-A79
all
reported
1,000
100
10
Number
Mrz-20
Mai-20
Jul-20
Sep-20
Nov-20
Jan-21
Mrz-21
Mai-21
Jul-21
Sep-21
Nov-21
Jan-22
Mrz-22
Mai-22
Jul-22
Sep-22
Nov-22
Variant
100%
75%
50%
25%
0%
WT
alpha
delta
BA1
BA2
BA4+5

Hesse

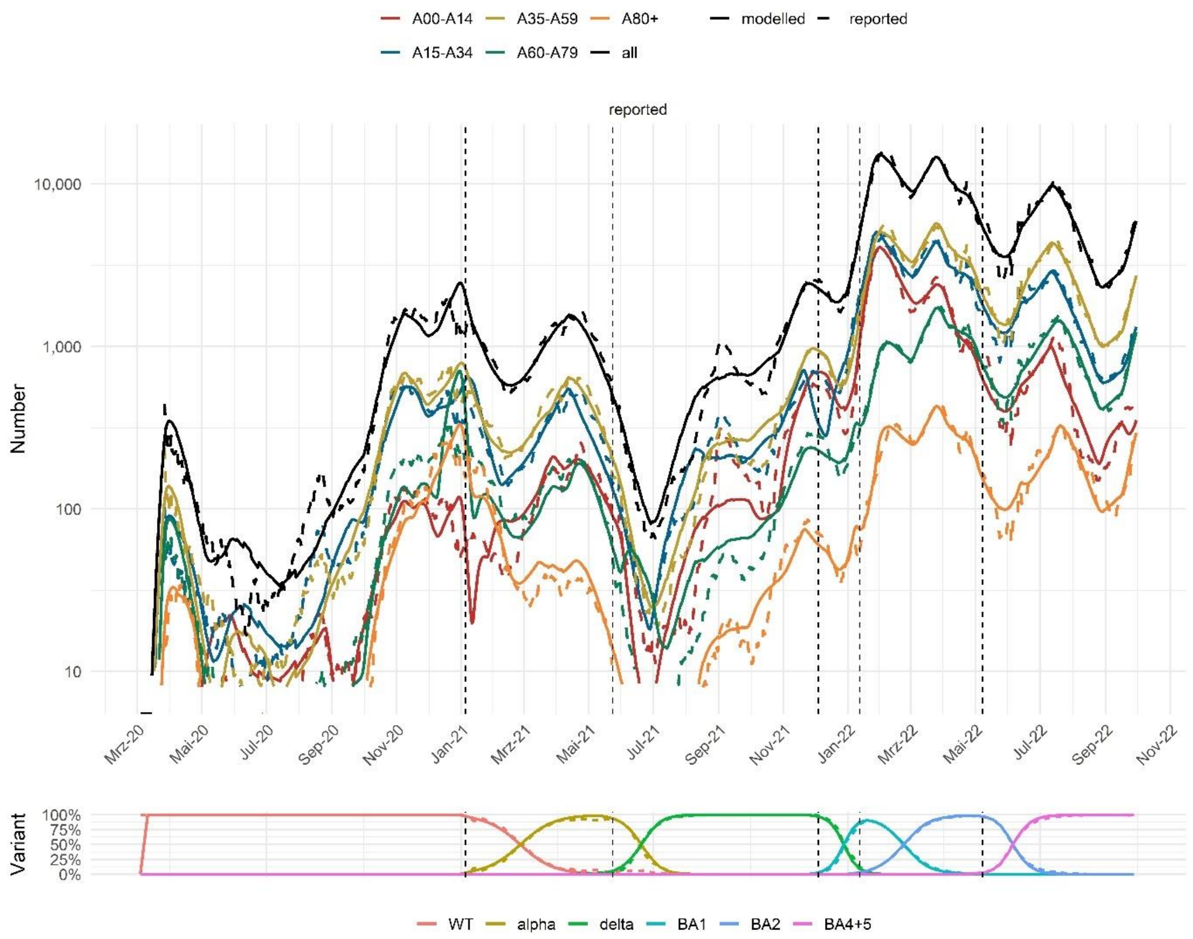
A00-A14
A35-A59
A80+
modelled
reported
A15-A34
A60-A79
all
reported
10,000
1,000
100
10
Number
Mrz-20
Mai-20
Jul-20
Sep-20
Nov-20
Jan-21
Mrz-21
Mai-21
Jul-21
Sep-21
Nov-21
Jan-22
Mrz-22
Mai-22
Jul-22
Sep-22
Nov-22
Variant
100%
75%
50%
25%
0%
WT
alpha
delta
BA1
BA2
BA4+5

Lower Saxony

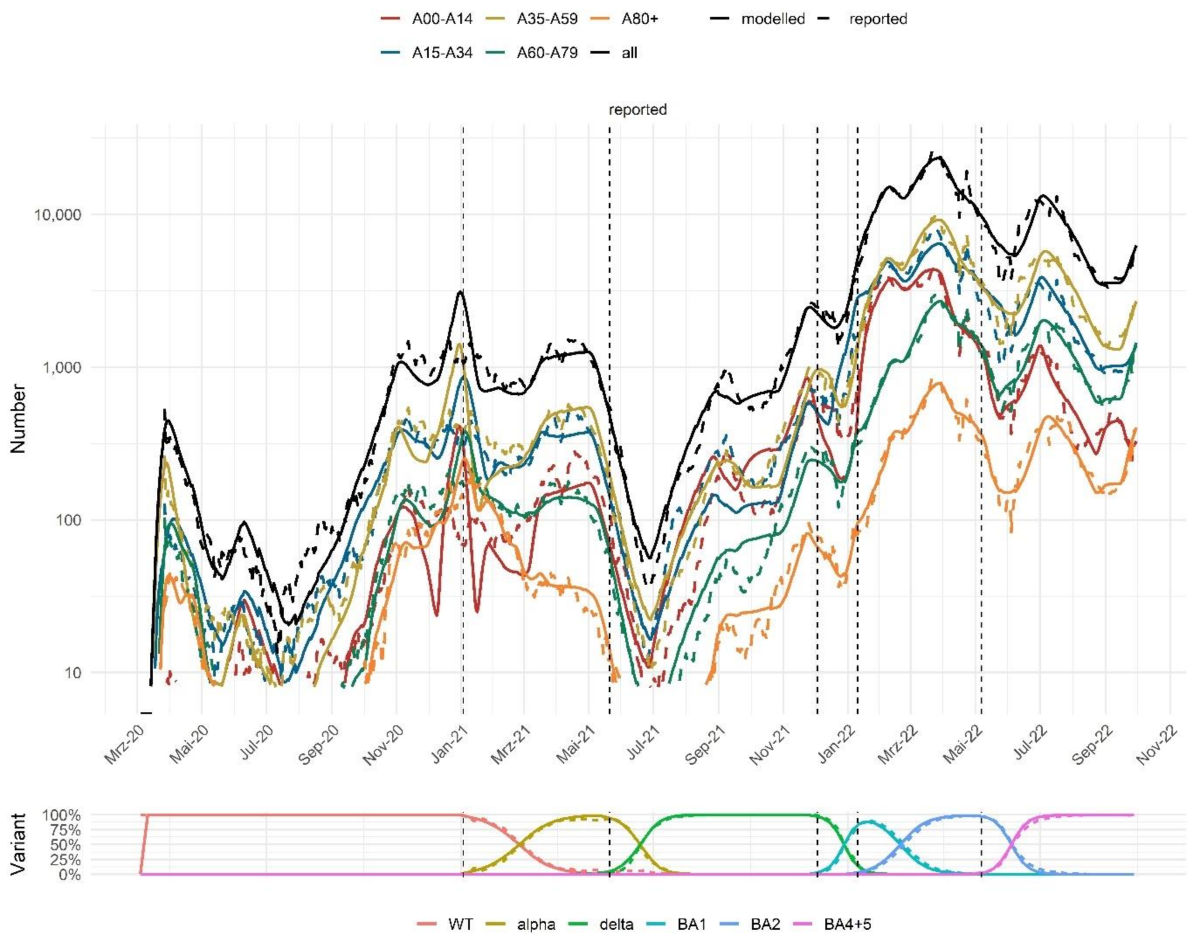

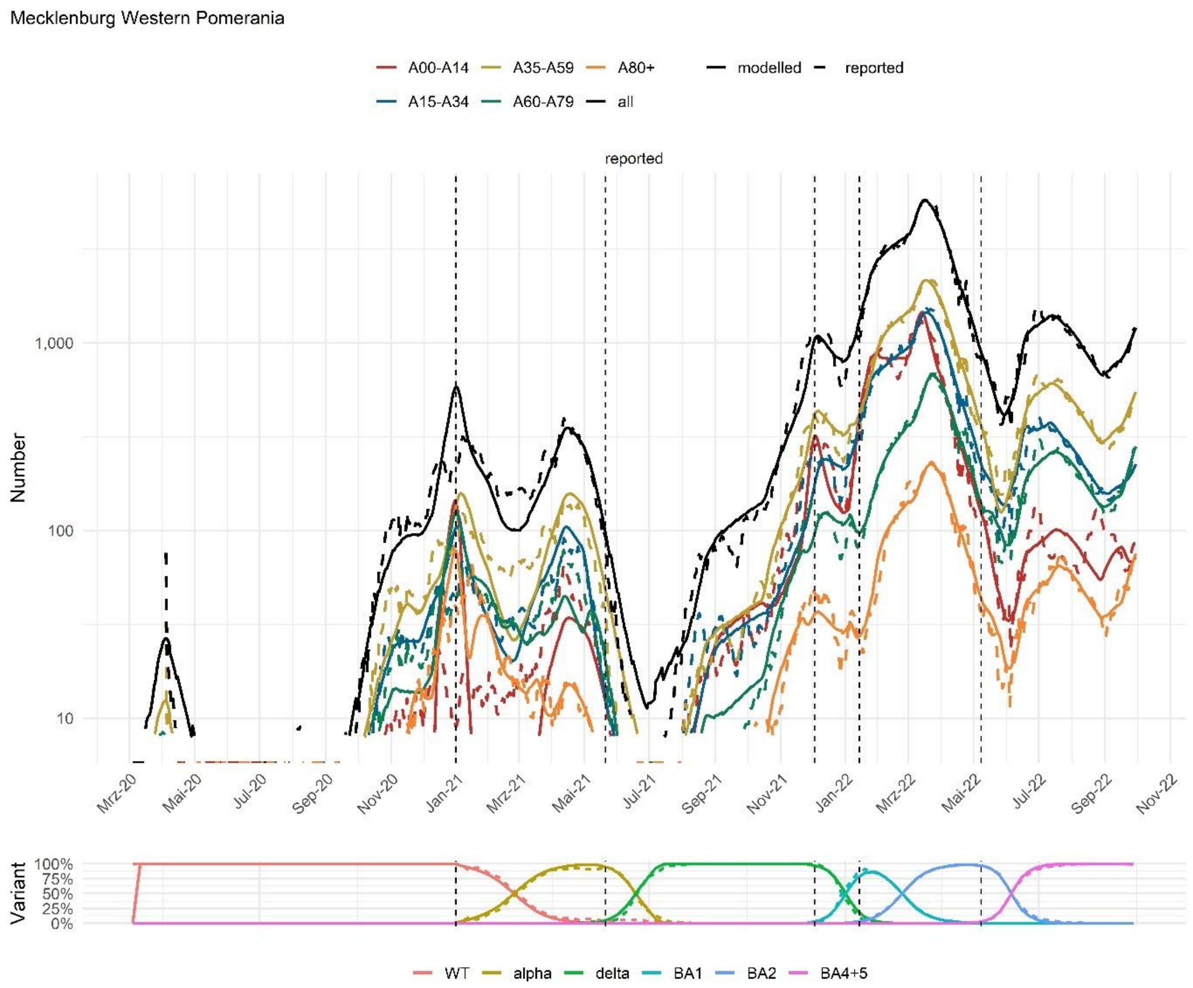
Mecklenburg Western Pomerania
A00-A14
A35-A59
A80+
modelled
reported
A15-A34
A60-A79
all
reported
Number
1,000
100
10
Mrz-20
Mai-20
Jul-20
Sep-20
Nov-20
Jan-21
Mrz-21
Mai-21
Jul-21
Sep-21
Nov-21
Jan-22
Mrz-22
Mai-22
Jul-22
Sep-22
Nov-22
Variant
100%
75%
50%
25%
0%
WT
alpha
delta
BA1
BA2
BA4+5

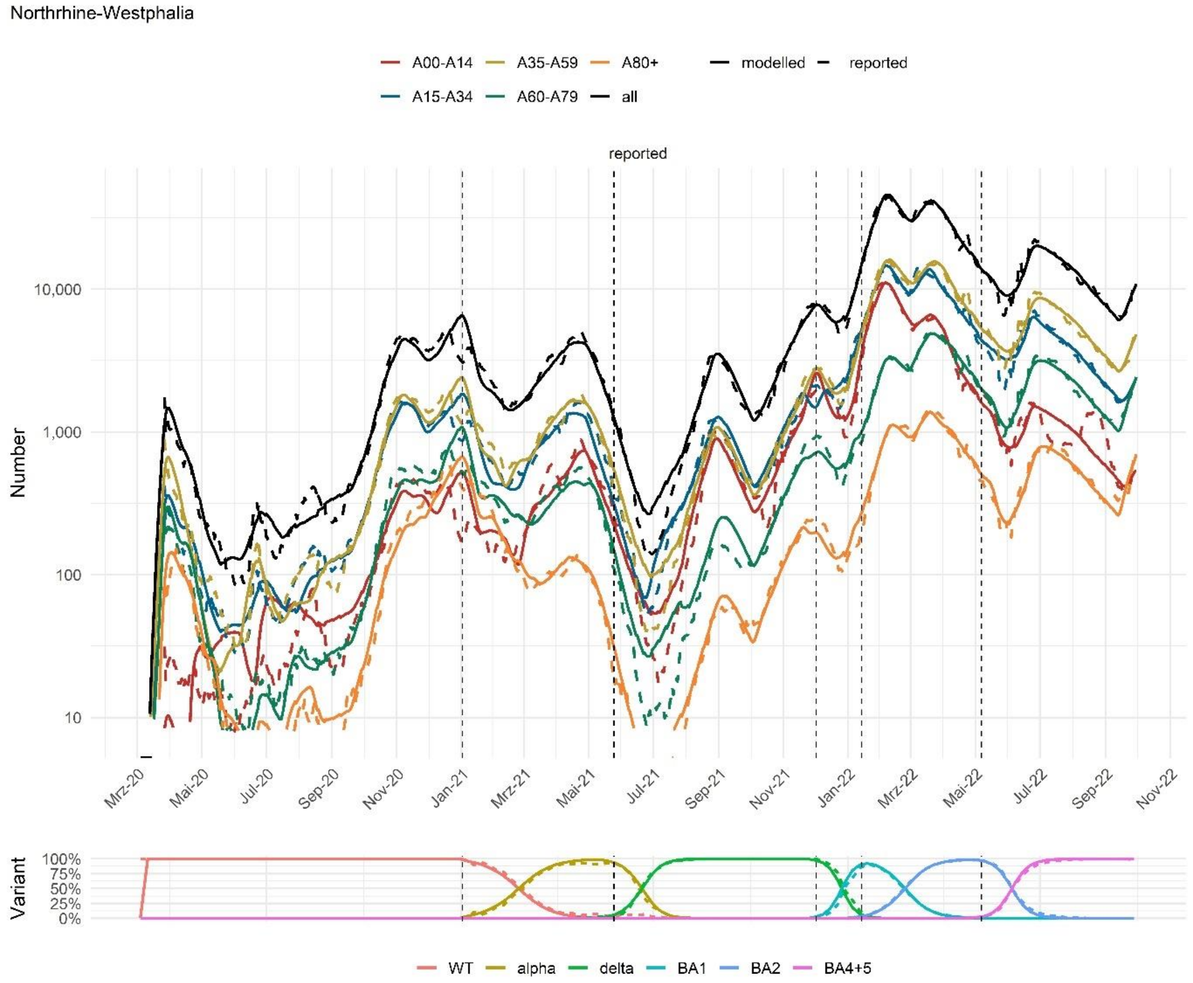
Northrhine-Westphalia
A00-A14
A35-A59
A80+
modelled
reported
A15-A34
A60-A79
all
reported
Number
10,000
1,000
100
10
Mrz-20
Mai-20
Jul-20
Sep-20
Nov-20
Jan-21
Mrz-21
Mai-21
Jul-21
Sep-21
Nov-21
Jan-22
Mrz-22
Mai-22
Jul-22
Sep-22
Nov-22
Variant
100%
75%
50%
25%
0%
WT
alpha
delta
BA1
BA2
BA4+5

Rhineland Palatinate

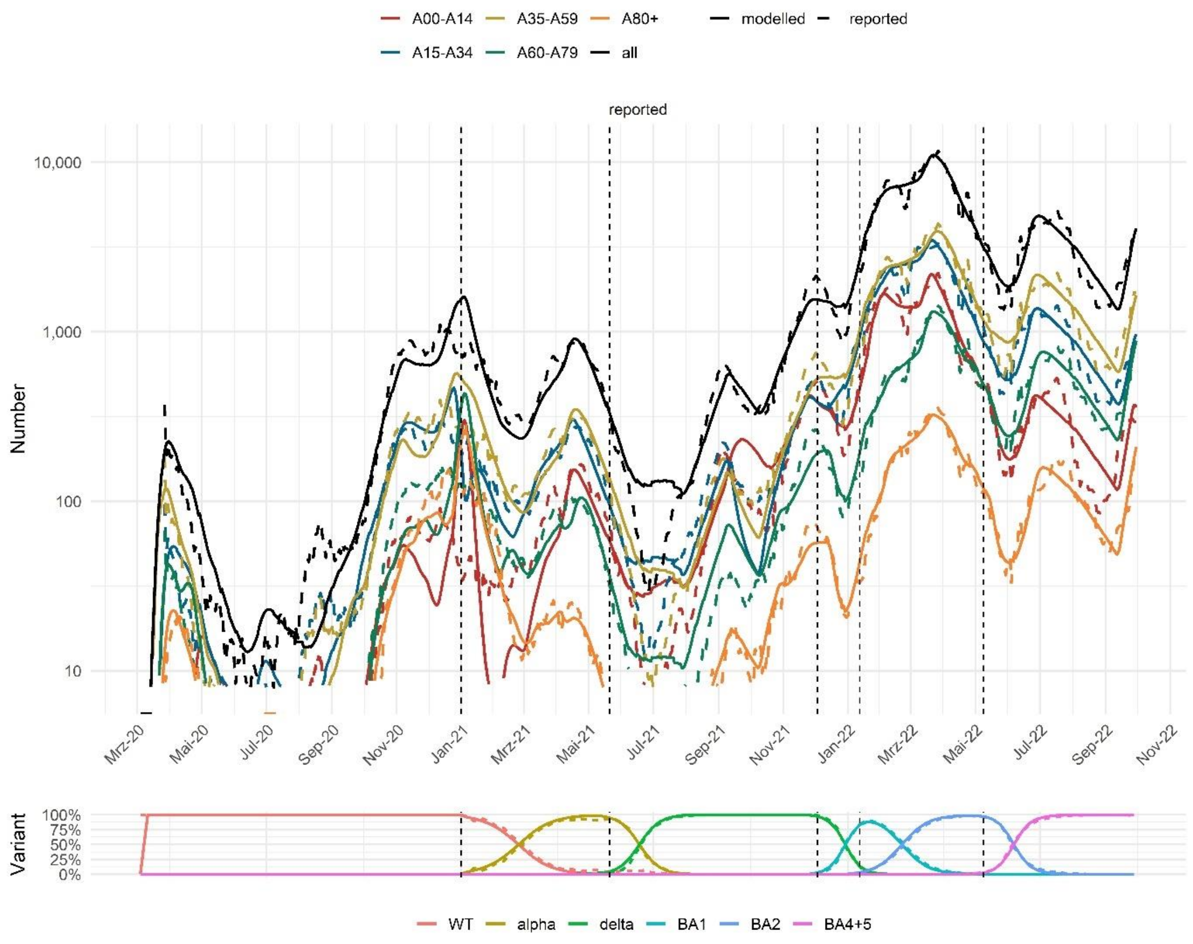

Saarland

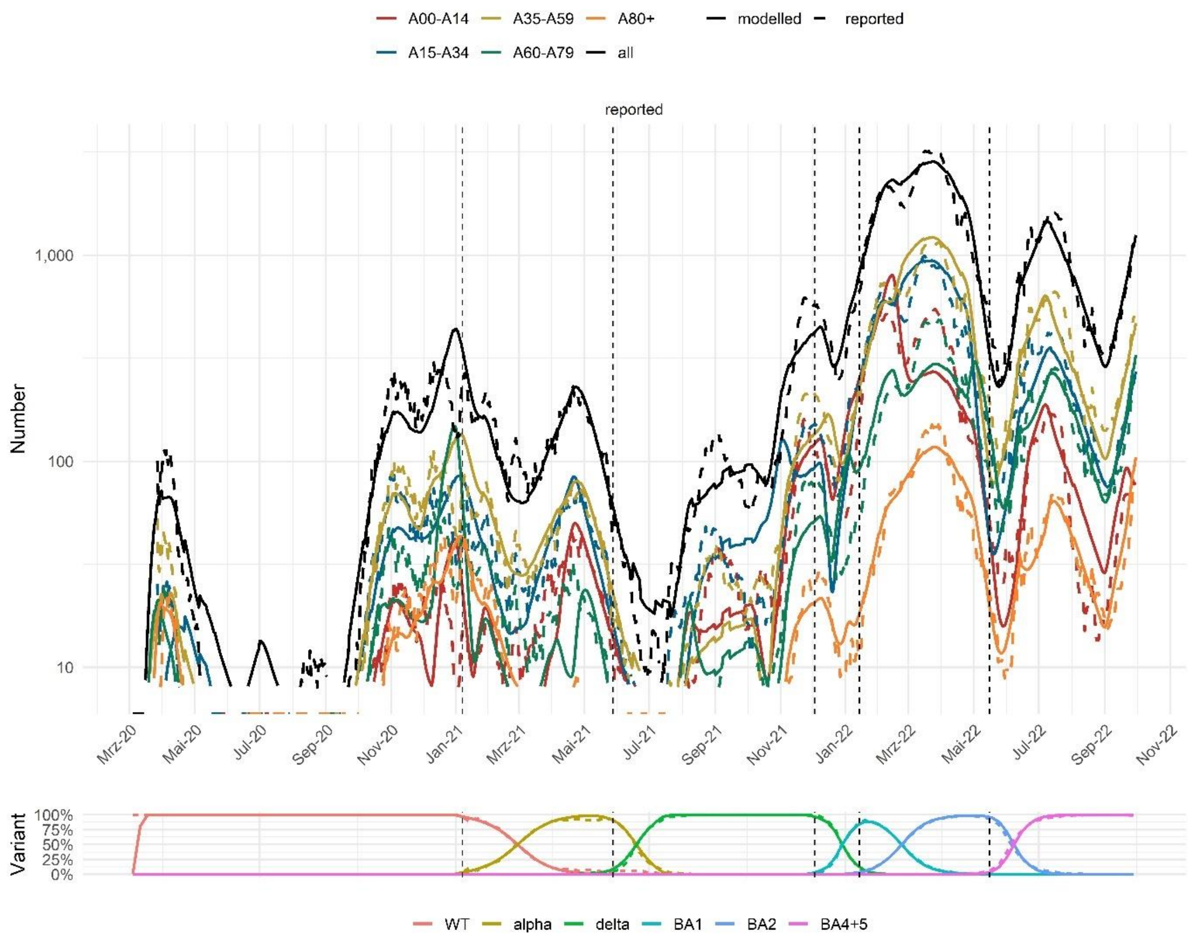
A00-A14
A35-A59
A80+
modelled
reported
A15-A34
A60-A79
all
reported
Number
1,000
100
10
Mrz-20
Mai-20
Jul-20
Sep-20
Nov-20
Jan-21
Mrz-21
Mai-21
Jul-21
Sep-21
Nov-21
Jan-22
Mrz-22
Mai-22
Jul-22
Sep-22
Nov-22
Variant
100%
75%
50%
25%
0%
WT
alpha
delta
BA1
BA2
BA4+5

Saxony

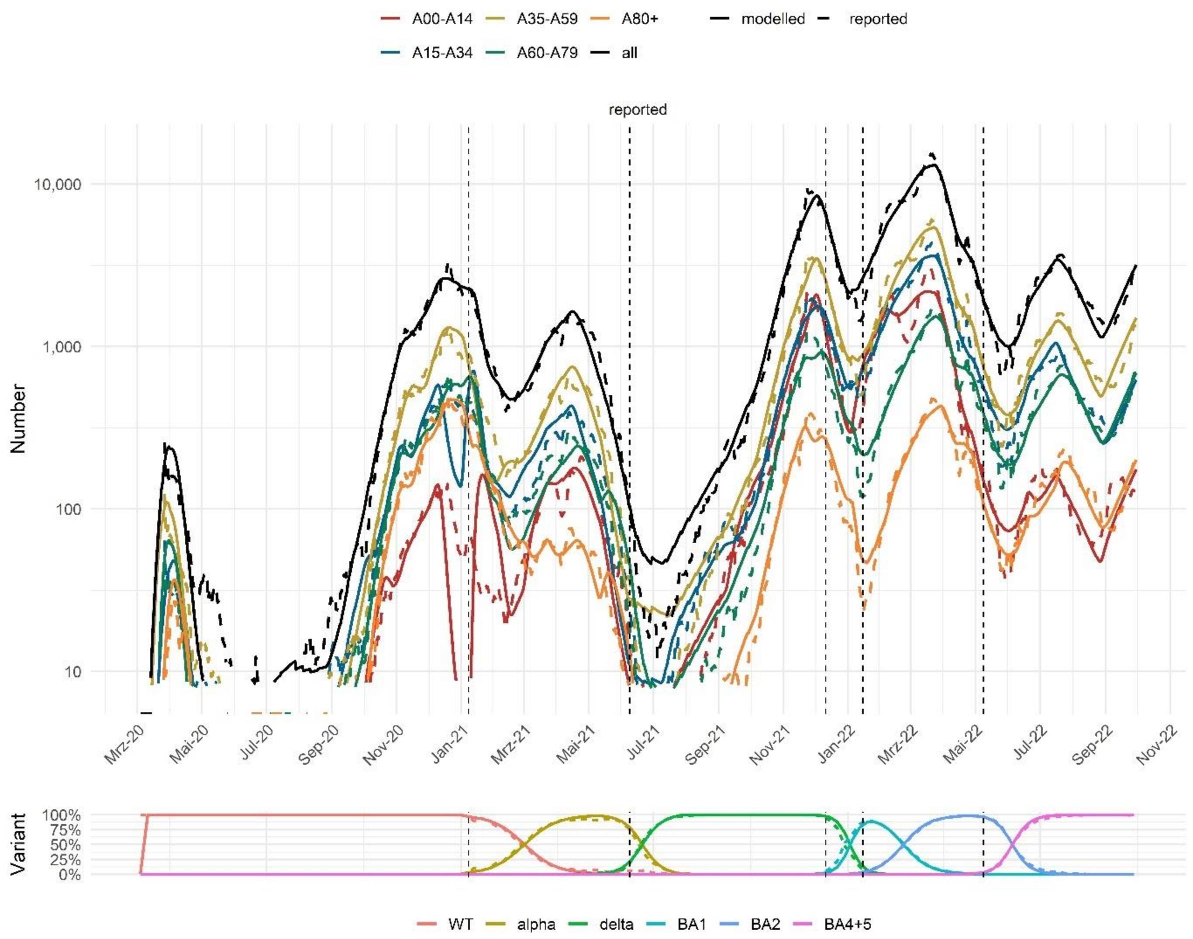
A00-A14
A35-A59
A80+
modelled
reported
A15-A34
A60-A79
all
reported
10,000
1,000
100
10
Number
Mrz-20
Mai-20
Jul-20
Sep-20
Nov-20
Jan-21
Mrz-21
Mai-21
Jul-21
Sep-21
Nov-21
Jan-22
Mrz-22
Mai-22
Jul-22
Sep-22
Nov-22
Variant
100%
75%
50%
25%
0%
WT
alpha
delta
BA1
BA2
BA4+5

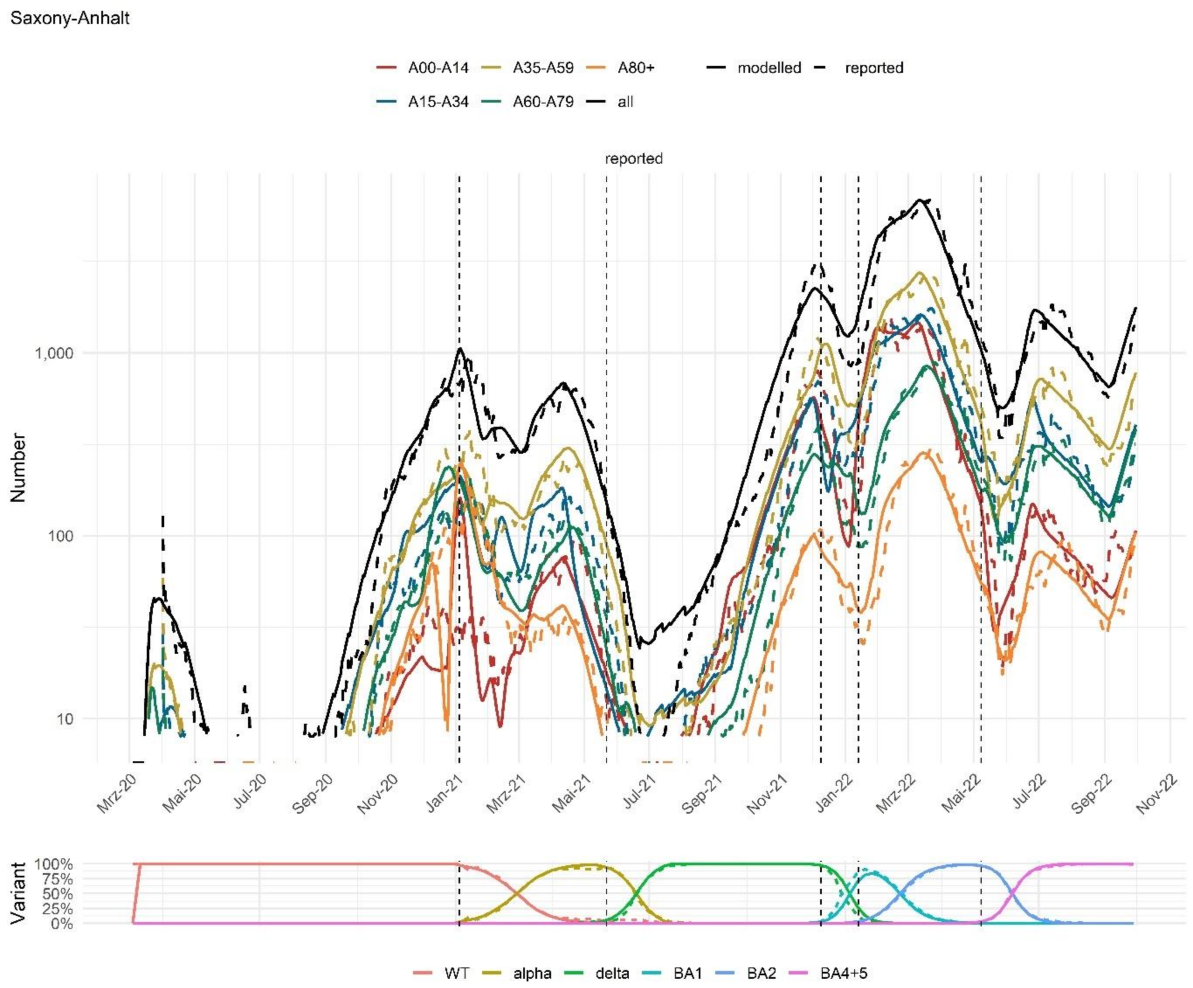
Saxony-Anhalt
A00-A14
A35-A59
A80+
A15-A34
A60-A79
all
modelled
reported
reported
Number
1,000
100
10
Mrz-20
Mai-20
Jul-20
Sep-20
Nov-20
Jan-21
Mrz-21
Mai-21
Jul-21
Sep-21
Nov-21
Jan-22
Mrz-22
Mai-22
Jul-22
Sep-22
Nov-22
Variant
100%
75%
50%
25%
0%
WT
alpha
delta
BA1
BA2
BA4+5

Schleswig Holstein

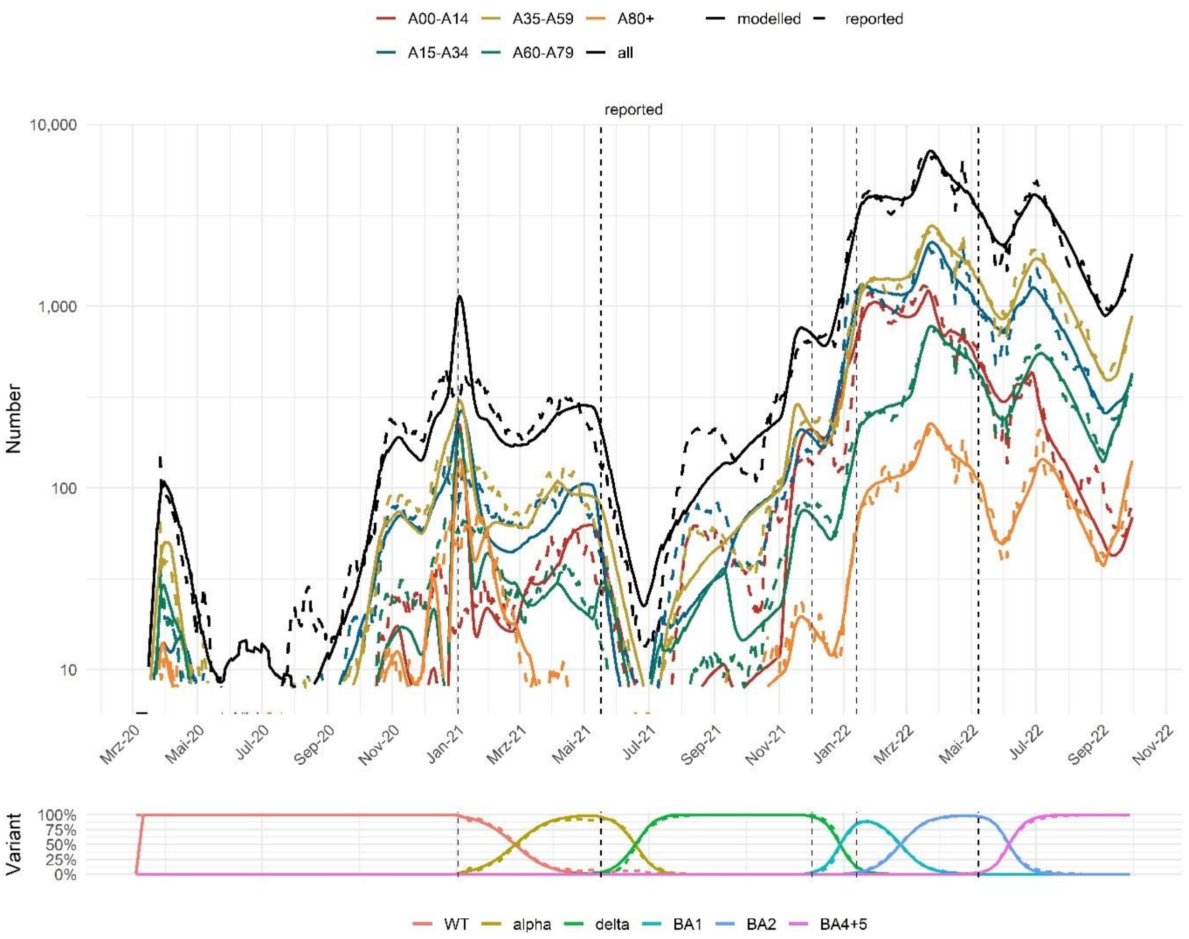

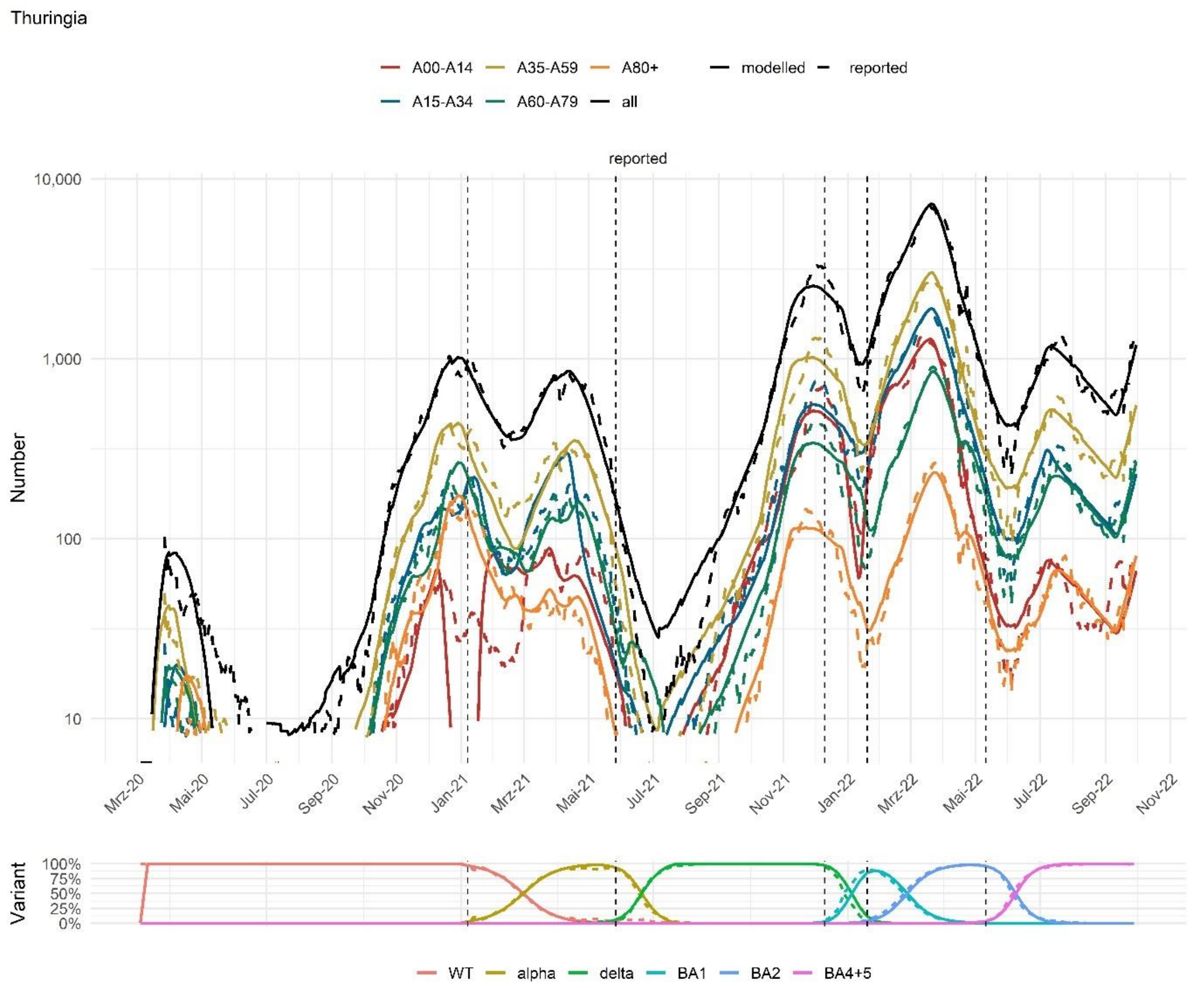
Thuringia
A00-A14
A35-A59
A80+
modelled
reported
A15-A34
A60-A79
all
reported
10,000
1,000
100
10
Number
Mrz-20
Mai-20
Jul-20
Sep-20
Nov-20
Jan-21
Mrz-21
Mai-21
Jul-21
Sep-21
Nov-21
Jan-22
Mrz-22
Mai-22
Jul-22
Sep-22
Nov-22
Variant
100%
75%
50%
25%
0%
WT
alpha
delta
BA1
BA2
BA4+5

Supplementary Material I. Agreement of model and data of severe disease courses per federal state

We present the agreement of model and data for severe disease courses per federal state and age group. While we present daily data of hospitalized and ICU cases, we present cumulative numbers of deaths.

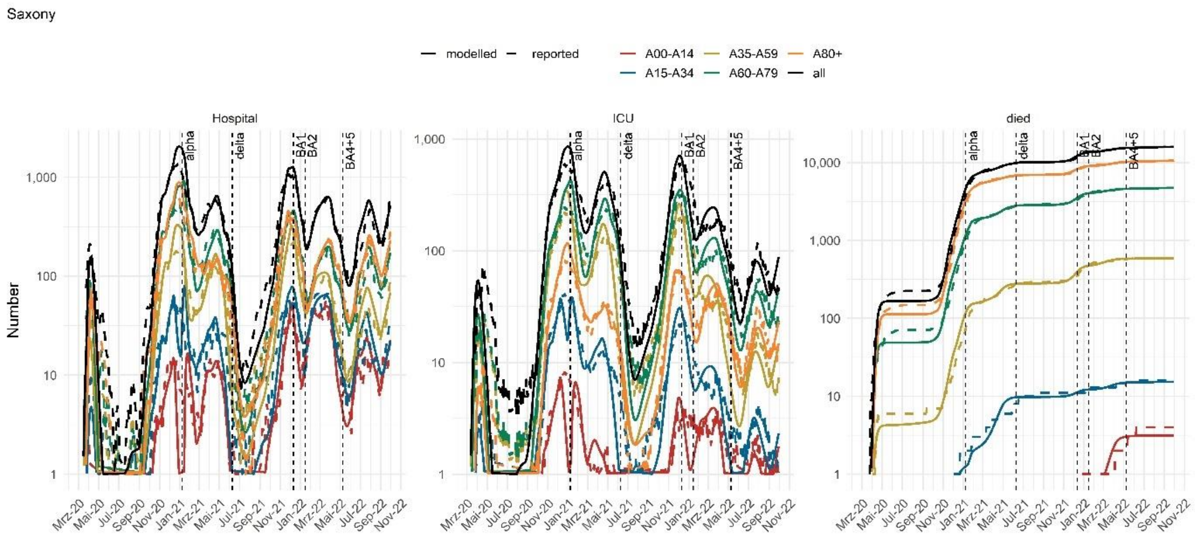

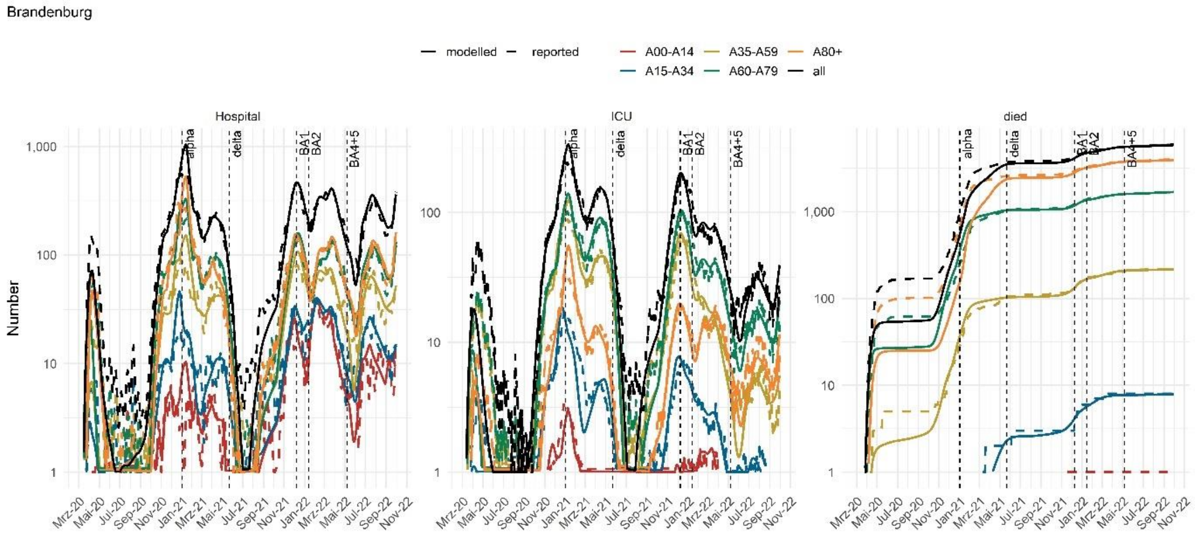

Lower Saxony

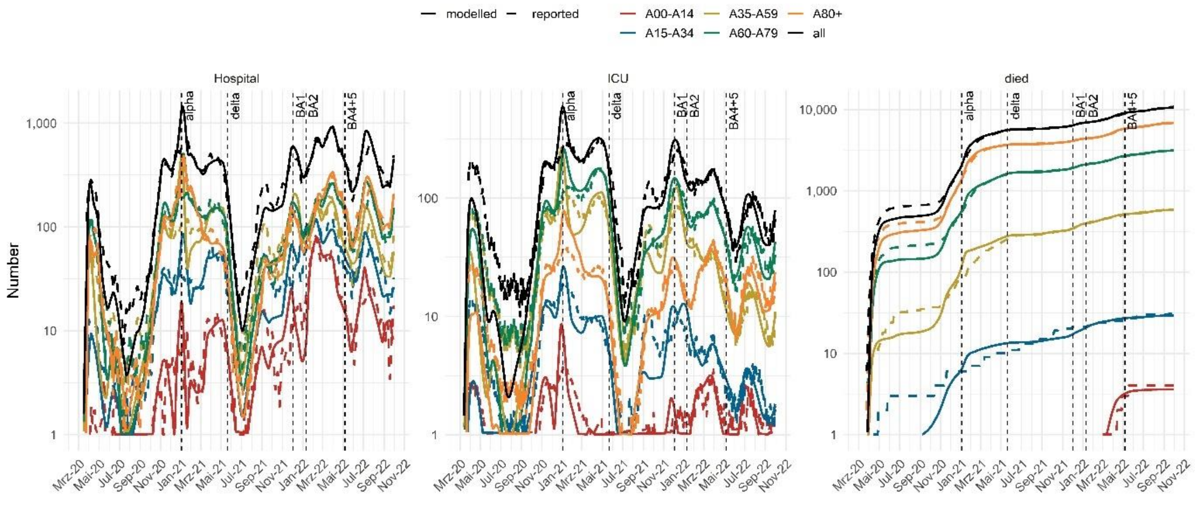

Saxony-Anhalt

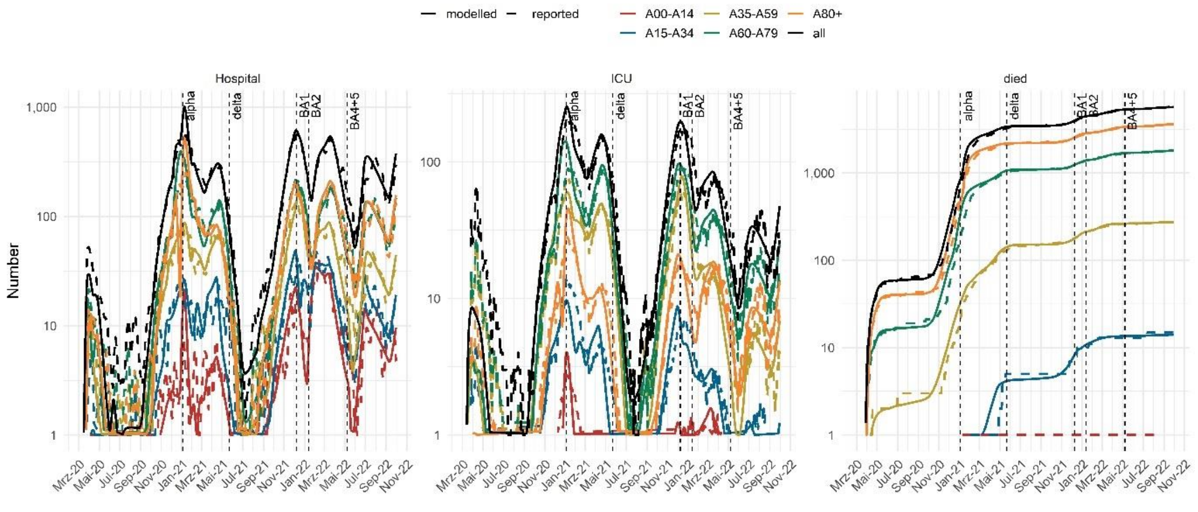

Thuringia

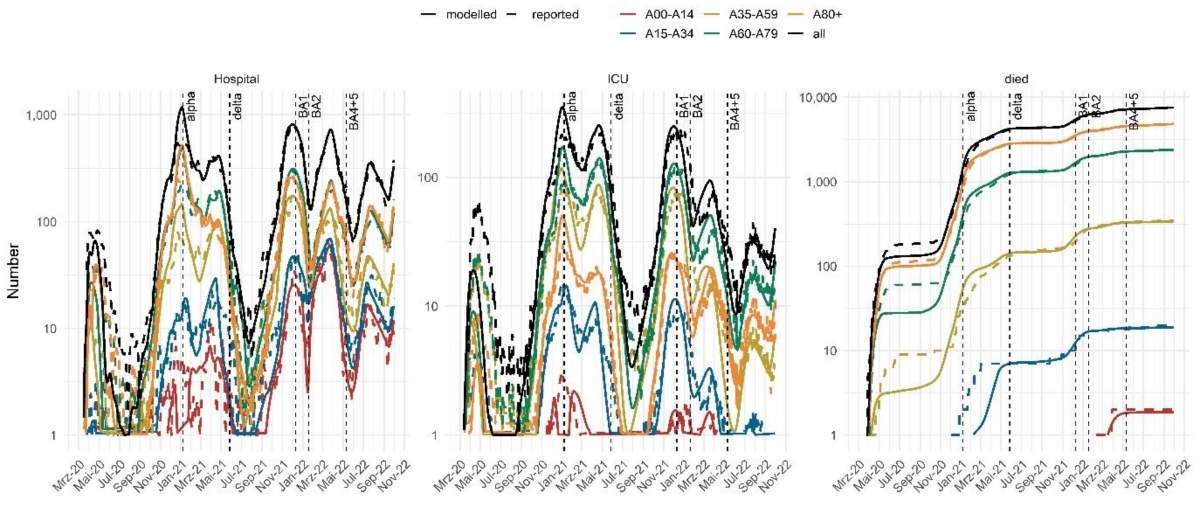

Baden-Württemberg

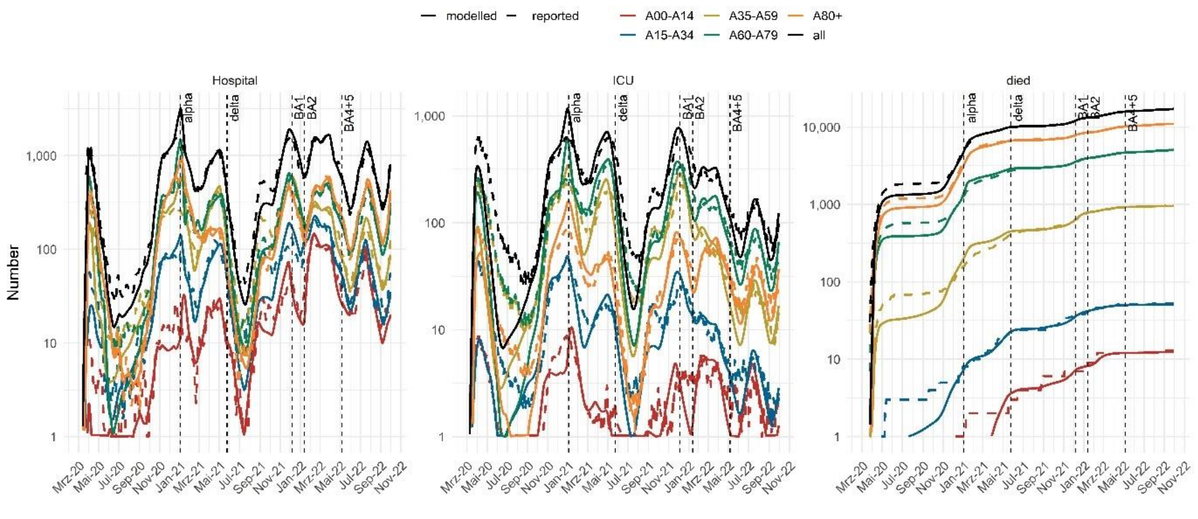

Bavaria

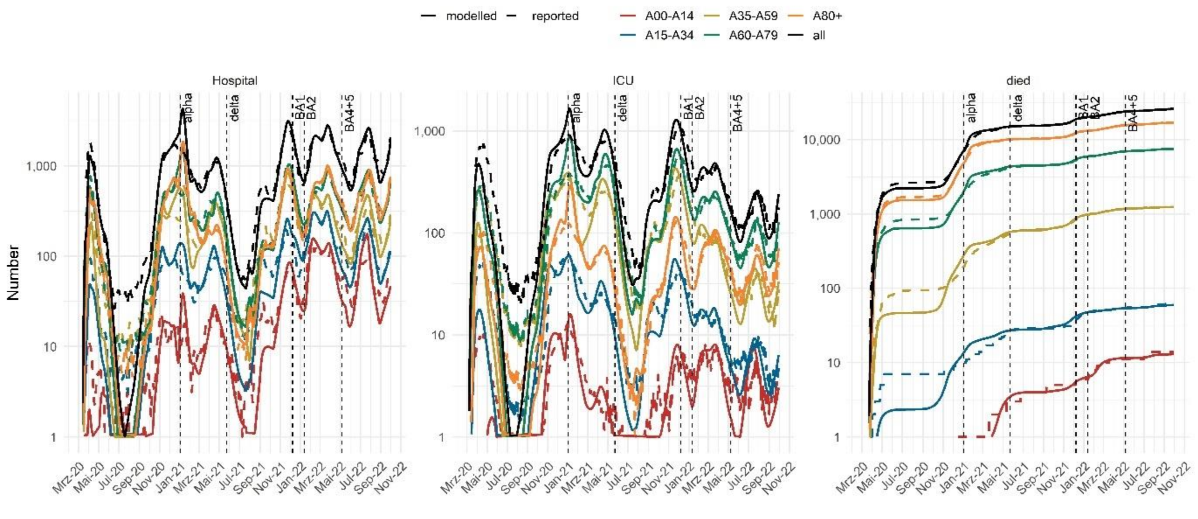

Rhineland Palatinate

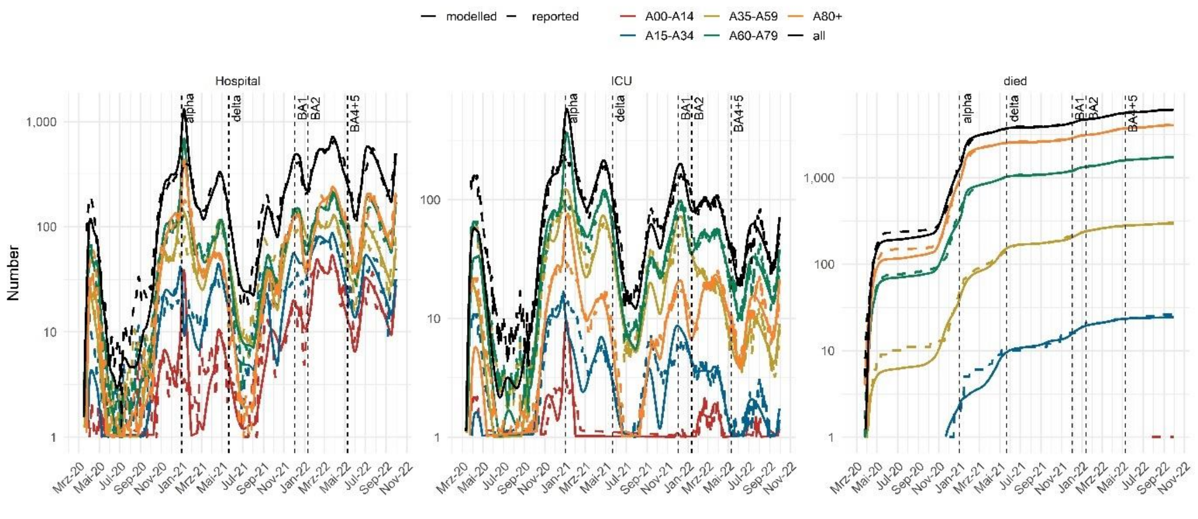

Hamburg

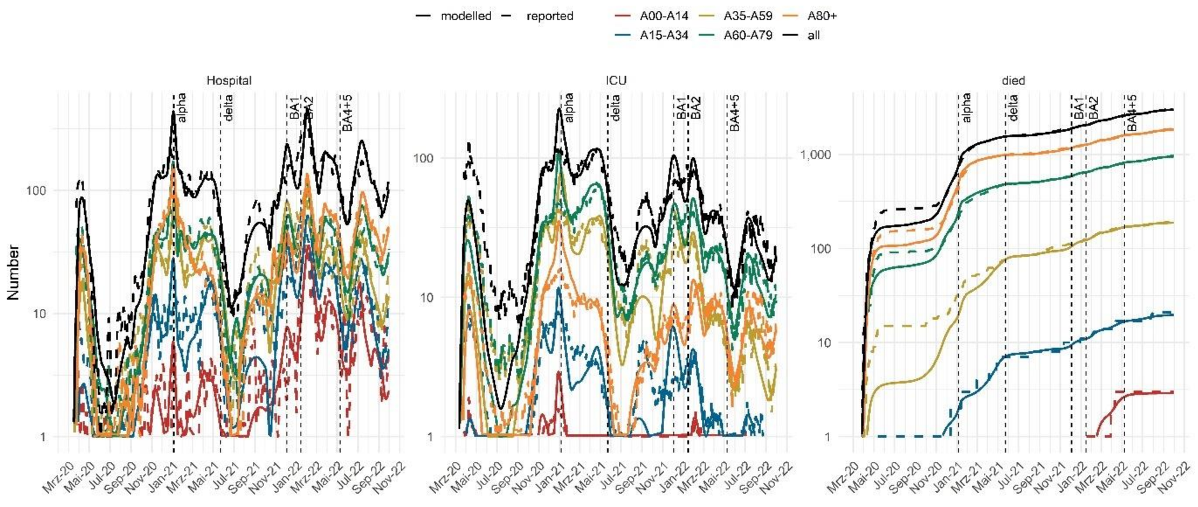

Bremen

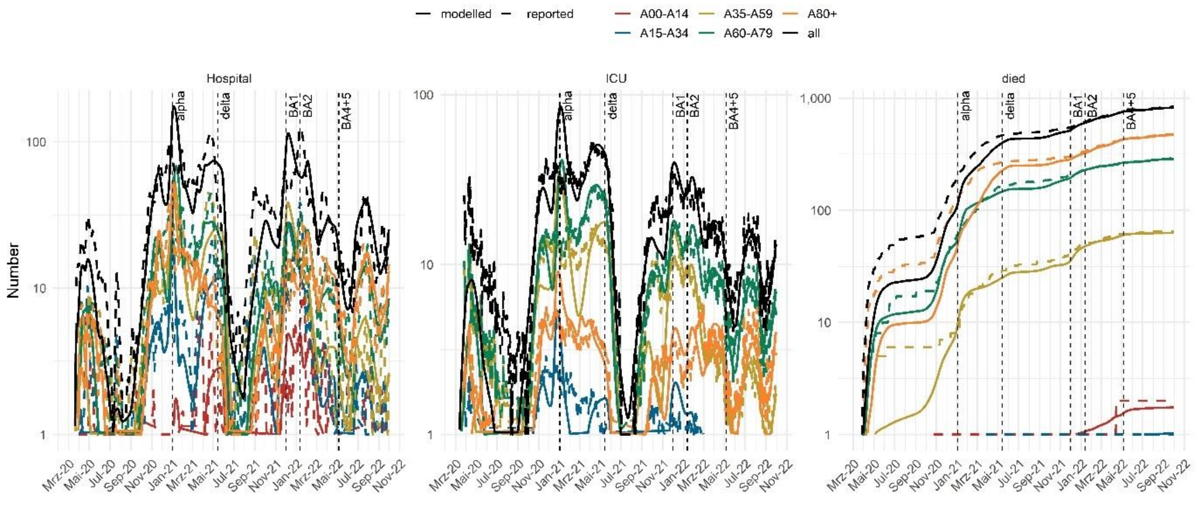

Berlin

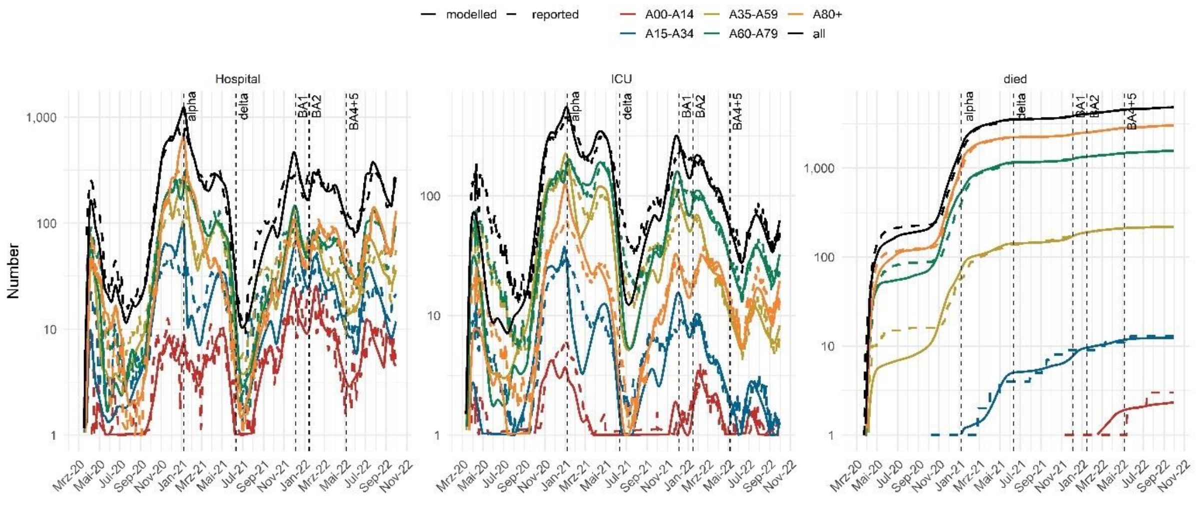

Saarland

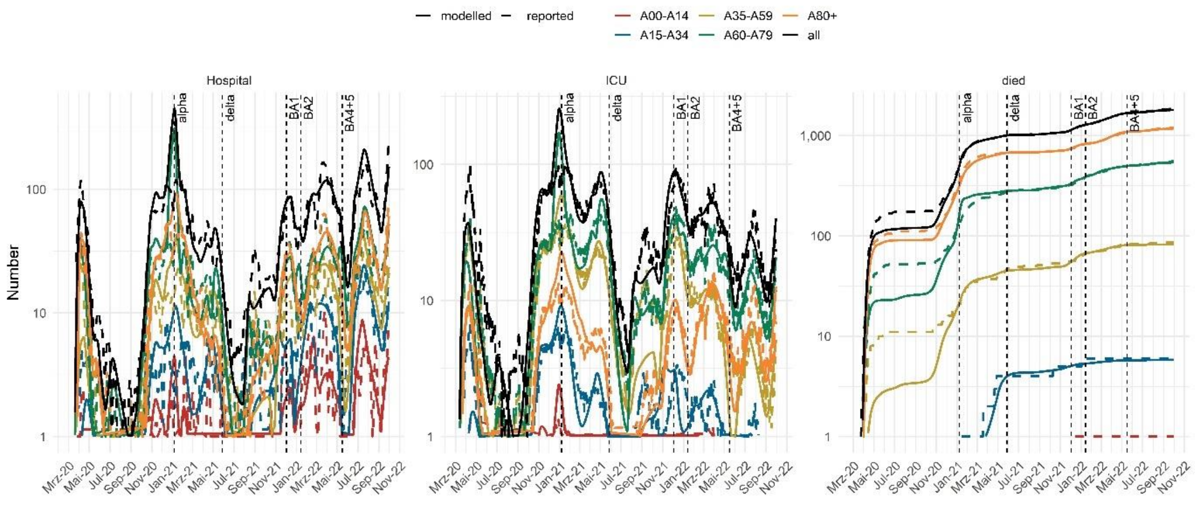

Mecklenburg Western Pomerania

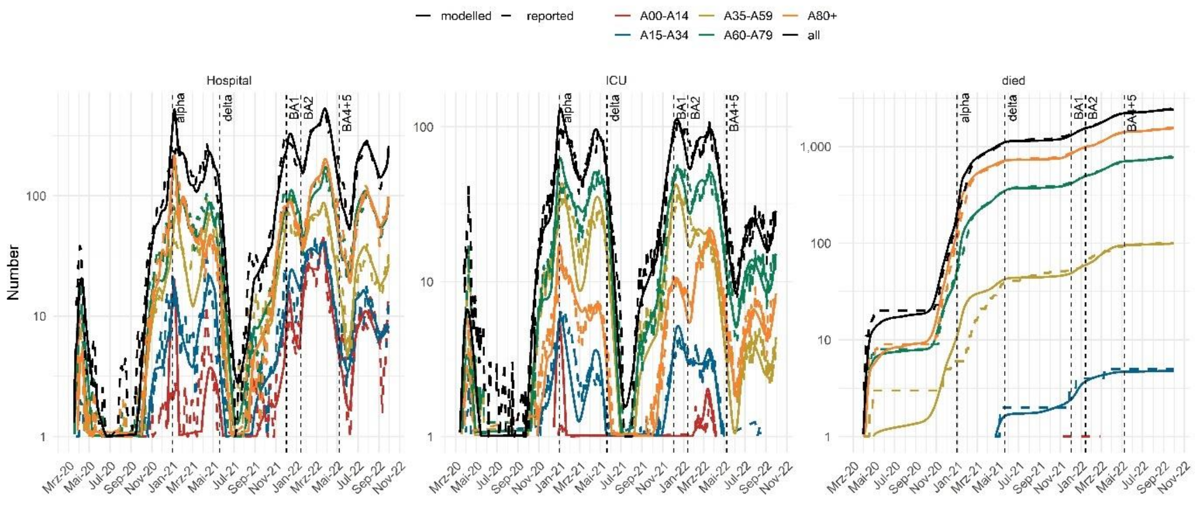

Schleswig Holstein

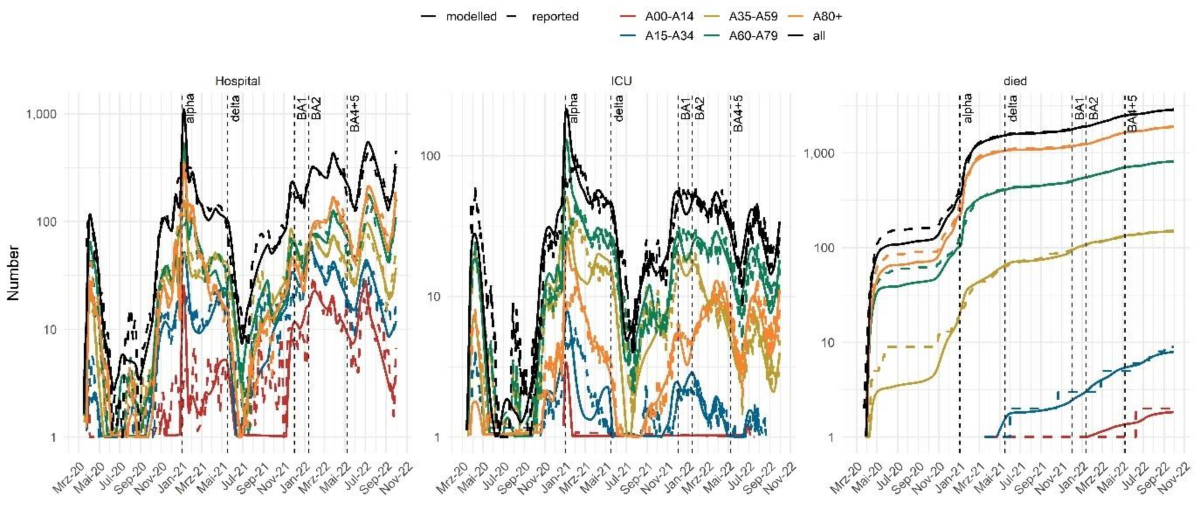

Northrhine-Westphalia

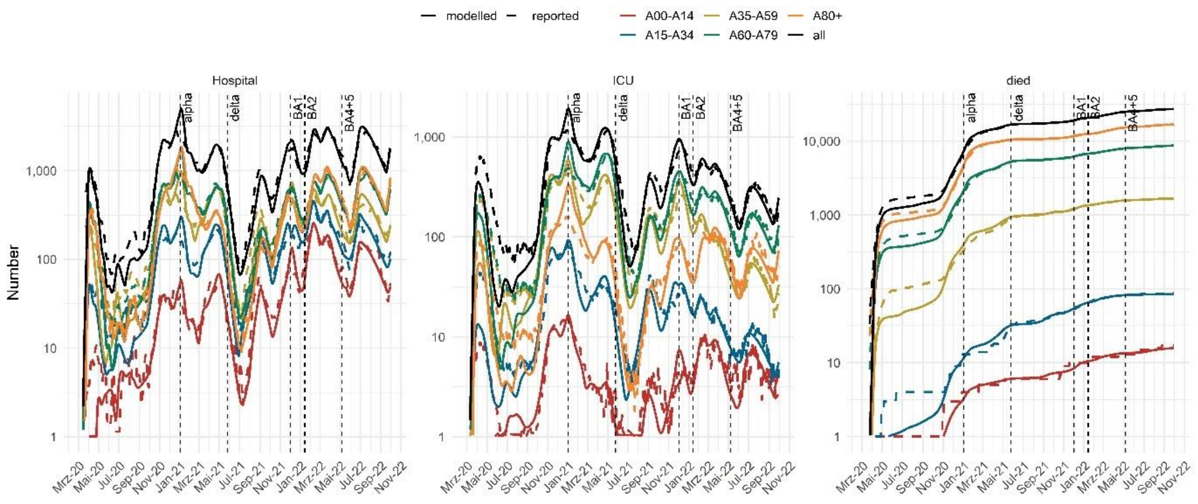

Hesse

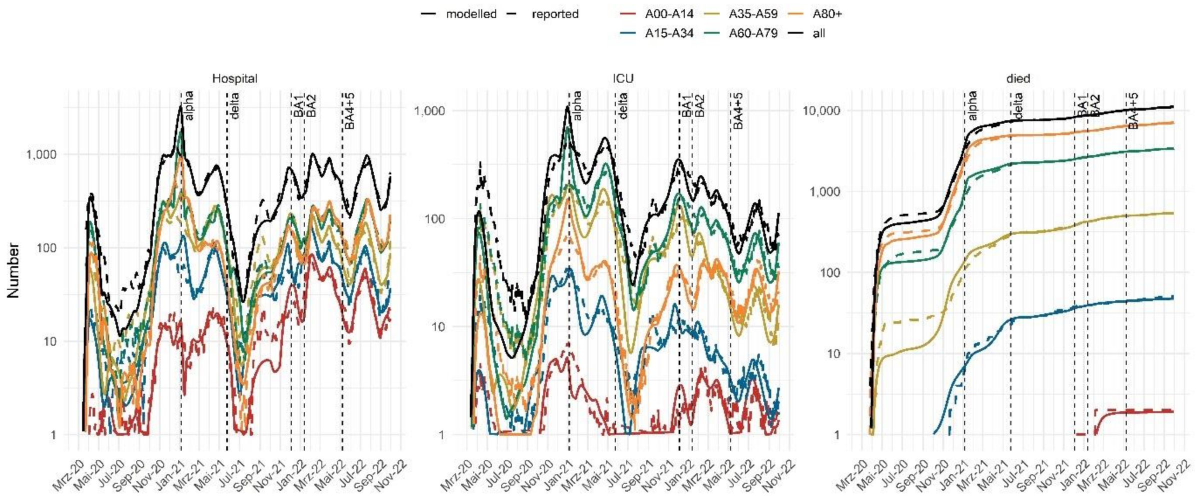

Supplementary Material J. Age-group specific dynamics of infectivity for Germany

Figures J1-J5 show the distributions of the parameter b_1 across federal states and compared to Germany. The distribution is derived based on the federal state-specific b_1 values.

Figure J1. Distribution of the dynamical parameter b_1 for the age group 0-14 years across the federal states between March 4th, 2020 and September 30th, 2022 and compared to the estimates obtain for Germany. *sd* corresponds to the standard deviation.

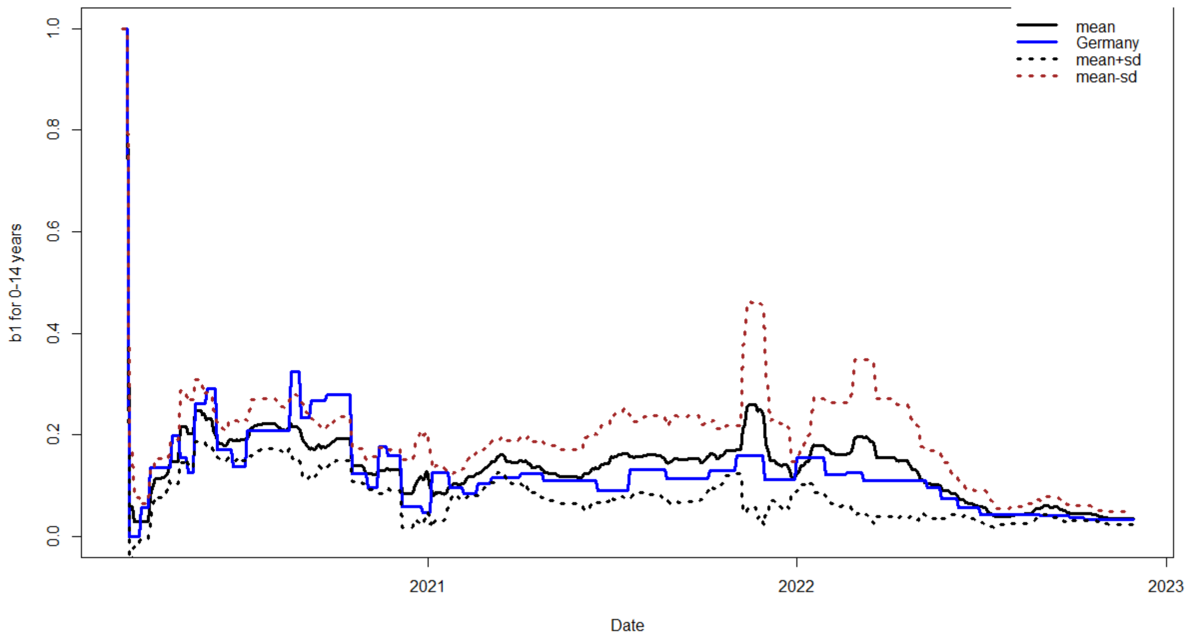

Figure J2. Distribution of the dynamical parameter b_1 for the age group 15-34 years across the federal states between March 4th, 2020 and September 30th, 2022 and compared to the estimates obtain for Germany. *sd* corresponds to the standard deviation.

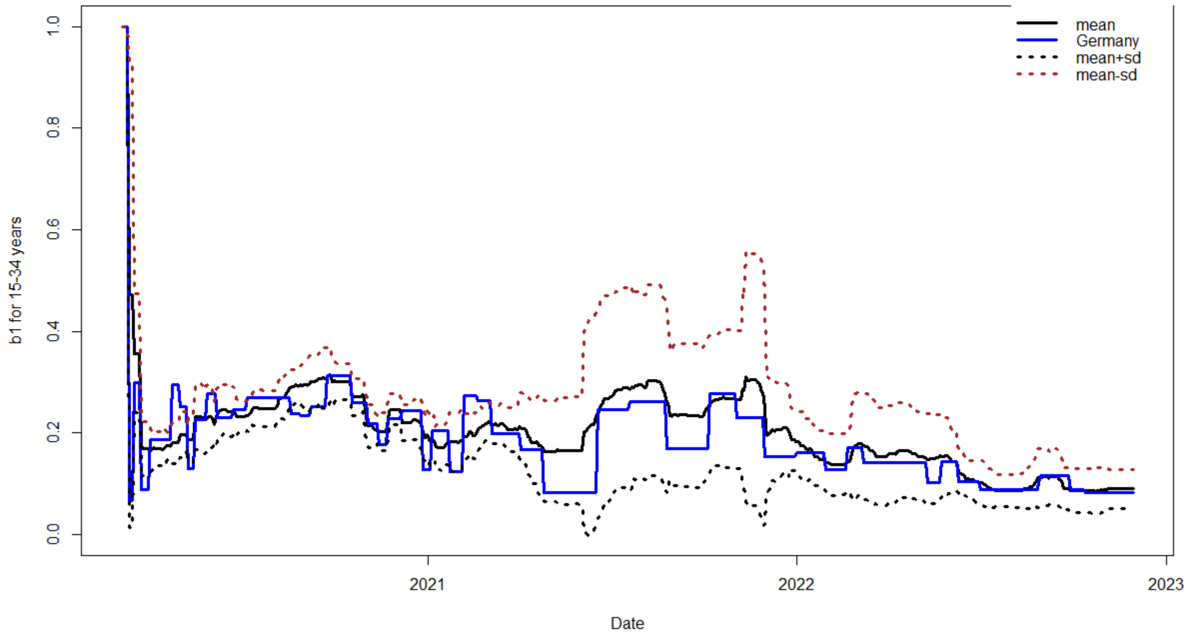

Figure J3. Distribution of the dynamical parameter b_1 for the age group 35-59 years across the federal states between March 4th, 2020 and September 30th, 2022 and compared to the estimates obtain for Germany. *sd* corresponds to the standard deviation.

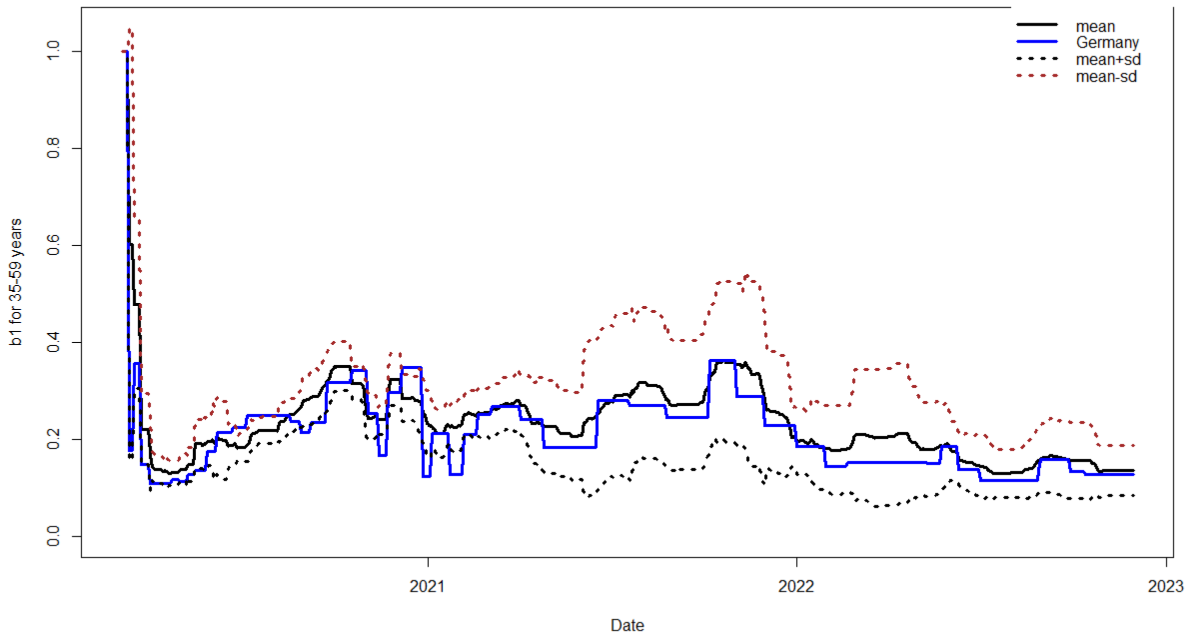

Figure J4. Distribution of the dynamical parameter b_1 for the age group 60-79 years across the federal states between March 4th, 2020 and September 30th, 2022 and compared to the estimates obtain for Germany. *sd* corresponds to the standard deviation.

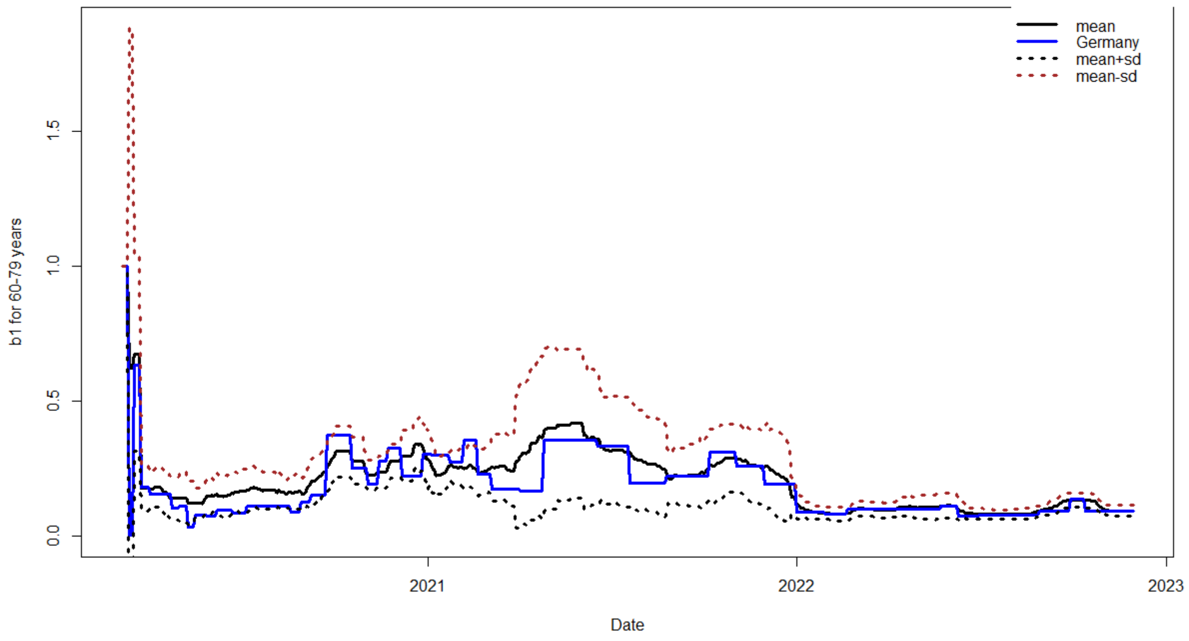

Figure J5. Distribution of the dynamical parameter b_1 for the age group 80+ years across the federal states between March 4th, 2020 and September 30th, 2022 and compared to the estimates obtain for Germany. *sd* corresponds to the standard deviation.

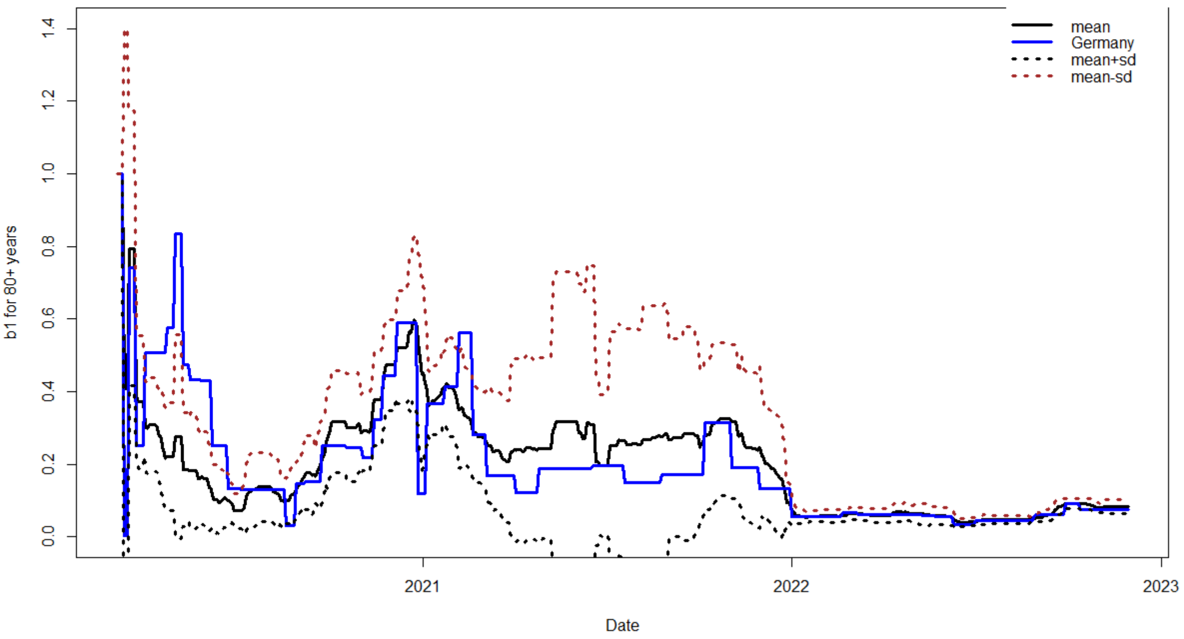
mean
Germany
mean+sd
mean-sd
b1 for 80+ years
0.0
0.2
0.4
0.6
0.8
1.0
1.2
1.4
2021
2022
2023
Date

Supplementary Material K. Age-stratified hospitalization transition rates across German federal states for different SARS-CoV-2 variants.

The figure shows the transition rate from infected (I_2) to hospitalized (H) cases across different age groups (A00-A14 to A80+) and German federal states. Data are shown for six viral variants: Wildtype, Alpha, Delta, BA1, BA2, and BA4+5. Each colored bar represents a different state, with rates plotted on a logarithmic scale. The x-axis represents the combined rates from infected compartment (I_2) to hospitalization (H) for patients who were immunologically naive prior to infection with each variant.

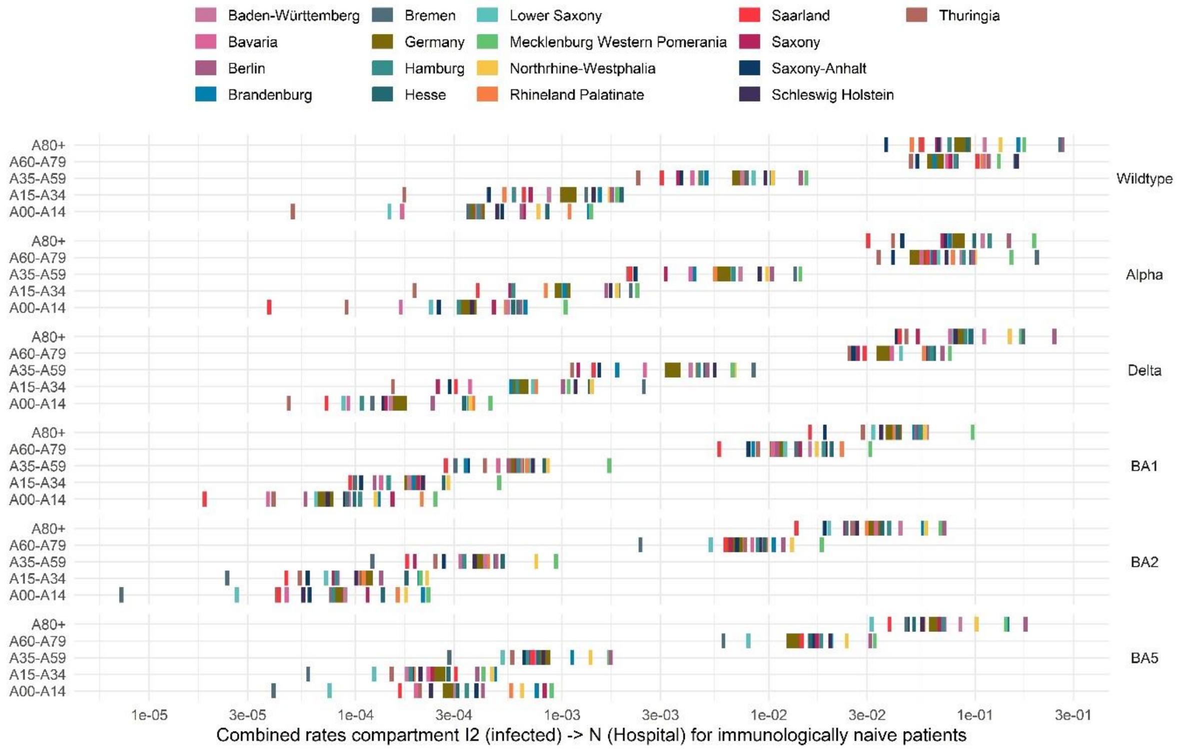

Supplementary Material L. Age-stratified ICU admission transition rates across German federal states for different SARS-CoV-2 variants.

The figure shows the transition rate from infected (I_2) to ICU (C) cases across different age groups (A00-A14 to A80+) and German federal states. Data are shown for six viral variants: Wildtype, Alpha, Delta, BA1, BA2, and BA4+5. Each colored bar represents a different state, with rates plotted on a logarithmic scale. The x-axis represents the combined rates from infected compartment (I_2) to ICU admission (C) for patients who were immunologically naive prior to infection with each variant.

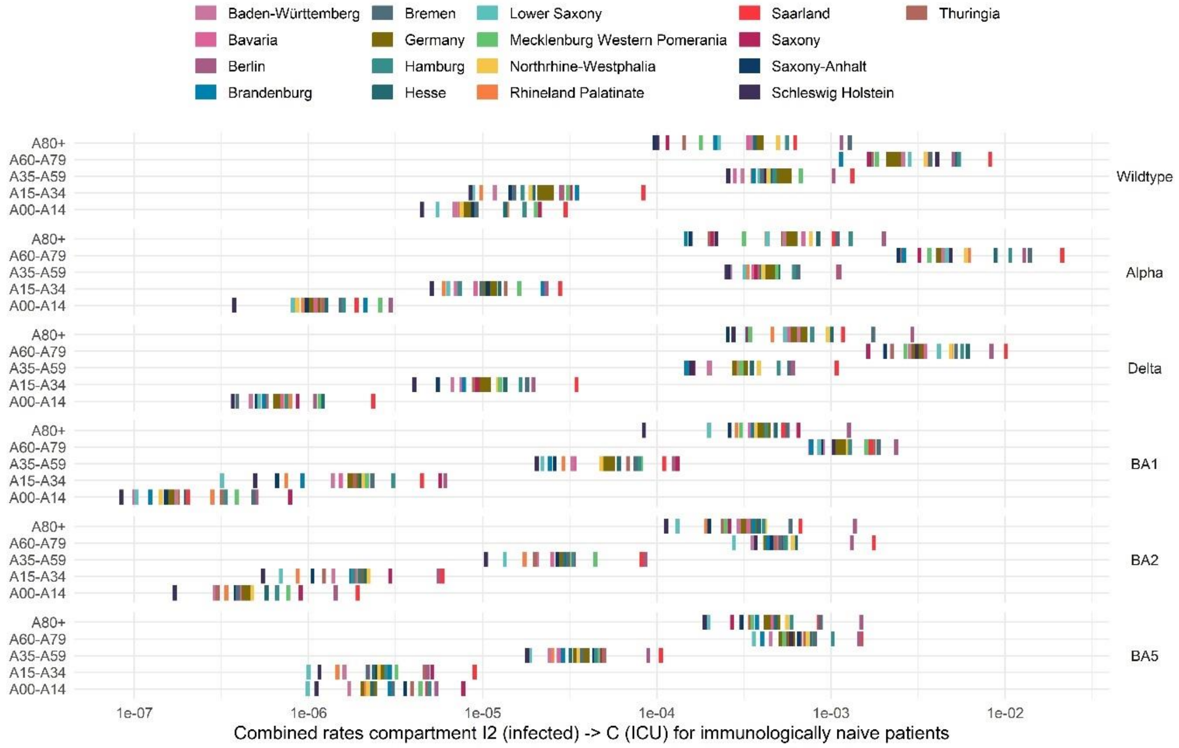

Supplementary Material M. Prediction of the dynamics of immune states for the federal states

We here present the dynamics of immune-states during the course of the pandemic, irrespective of age. We also present corresponding dynamics of vaccination and booster campaigns.

The figures show modeled temporal changes in the immune status of the population for all 16 German states. In the respective top panel, we provide the distribution of immune states in the population: susceptible and unvaccinated (S/Vac_0), infected (D), vaccinated (Vac_1) or recovered from infection (R_1), reduced immunity due to waning (Vac_2 and R_2), and further reduced immunity after further waning (Vac_3 and R_3). The middle panel tracks reported vaccination progress, comparing primary immunization (typically first two doses, solid line) with one or more booster doses (dashed line). The bottom panel shows cumulative modeled infections. Data span from July 2020 to July 2022, with vertical dashed lines indicating the emergence of new variants.

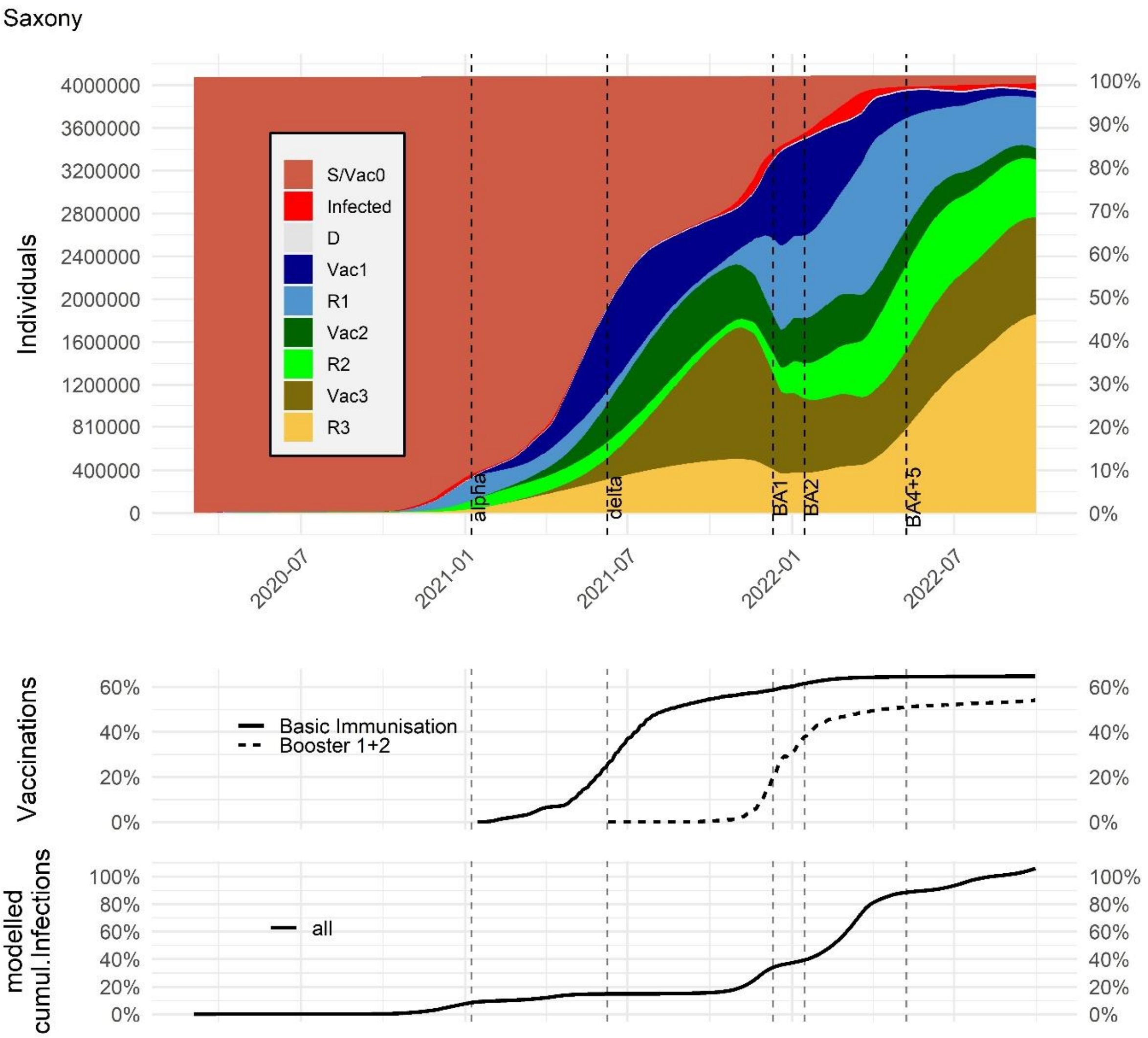

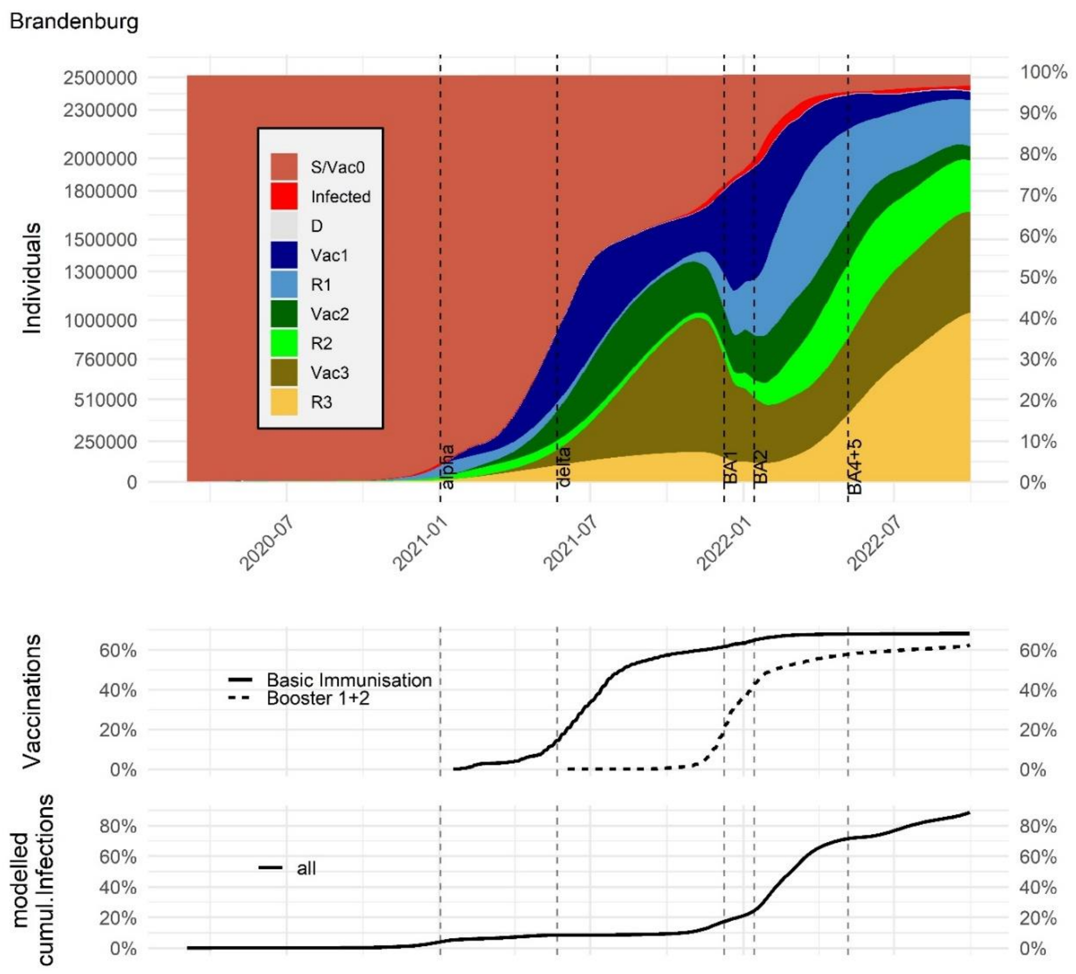
Brandenburg
Individuals
2500000
2300000
2000000
1800000
1500000
1300000
1000000
760000
510000
250000
0
100%
90%
80%
70%
60%
50%
40%
30%
20%
10%
0%
S/Vac0
Infected
D
Vac1
R1
Vac2
R2
Vac3
R3
alpha
delta
BA1
BA2
BA4+5
2020-07
2021-01
2021-07
2022-01
2022-07
Vaccinations
60%
40%
20%
0%
Basic Immunisation
Booster 1+2
modelled cumul.Infections
80%
60%
40%
20%
0%
all

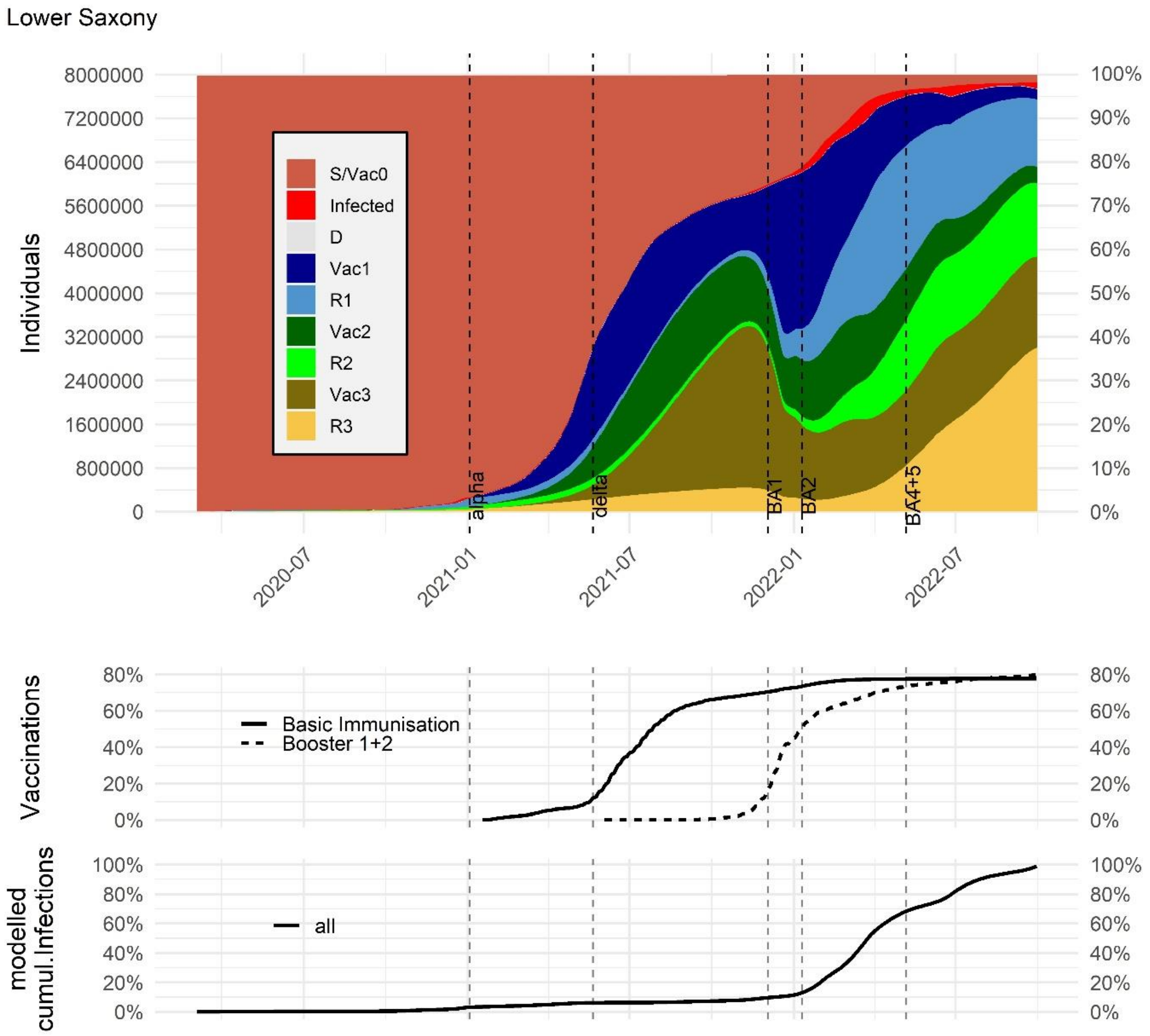
Lower Saxony
Individuals
8000000
7200000
6400000
5600000
4800000
4000000
3200000
2400000
1600000
800000
0
100%
90%
80%
70%
60%
50%
40%
30%
20%
10%
0%
S/Vac0
Infected
D
Vac1
R1
Vac2
R2
Vac3
R3
alpha
delta
BA1
BA2
BA4+5
2020-07
2021-01
2021-07
2022-01
2022-07
Vaccinations
Basic Immunisation
Booster 1+2
modelled cumul.Infections
all

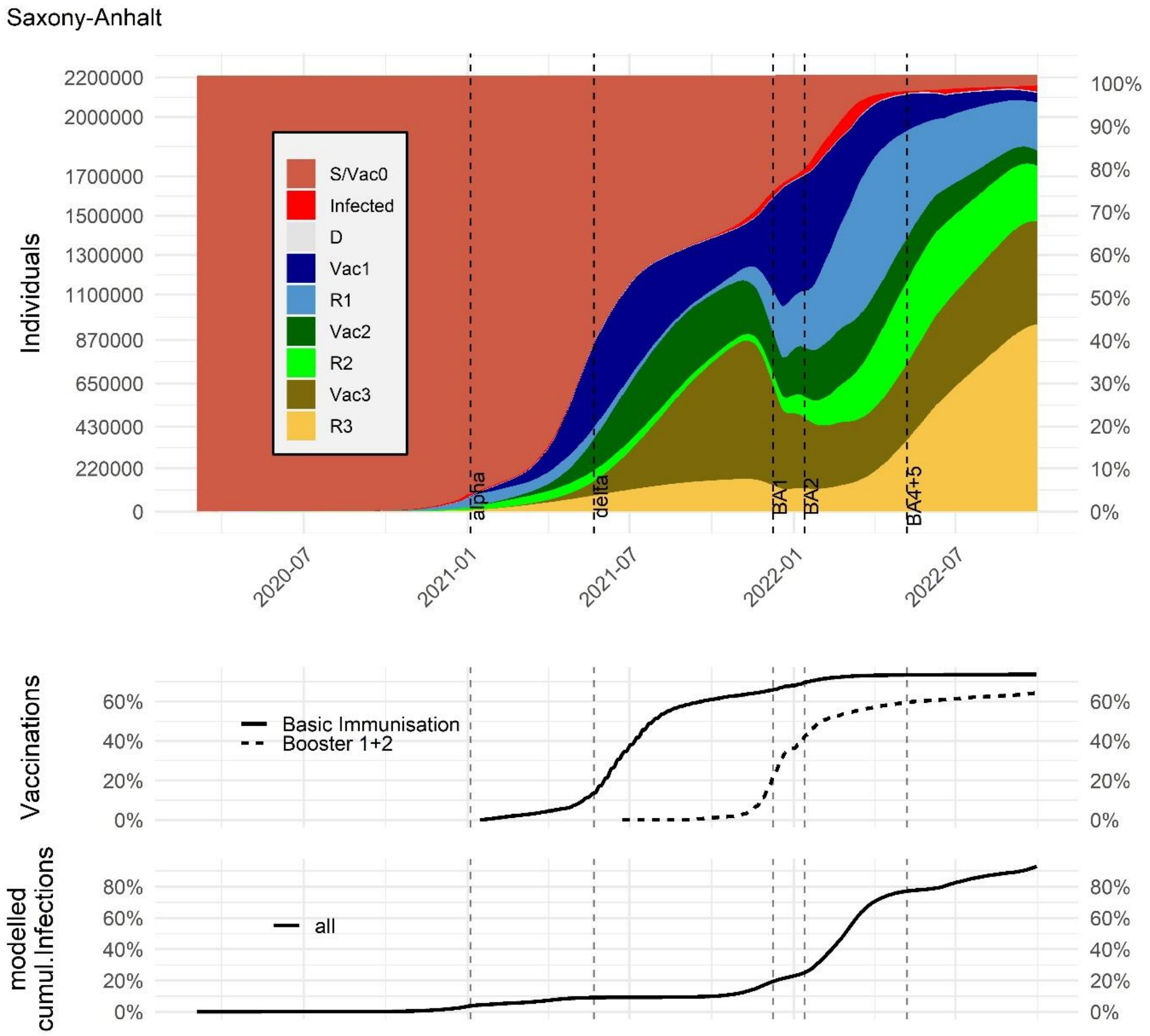
Saxony-Anhalt
Individuals
2200000
2000000
1700000
1500000
1300000
1100000
870000
650000
430000
220000
0
100%
90%
80%
70%
60%
50%
40%
30%
20%
10%
0%
S/Vac0
Infected
D
Vac1
R1
Vac2
R2
Vac3
R3
alpha
delta
BA1
BA2
BA4+5
2020-07
2021-01
2021-07
2022-01
2022-07
Vaccinations
Basic Immunisation
Booster 1+2
60%
40%
20%
0%
modelled cumul.Infections
all
80%
60%
40%
20%
0%

Thuringia

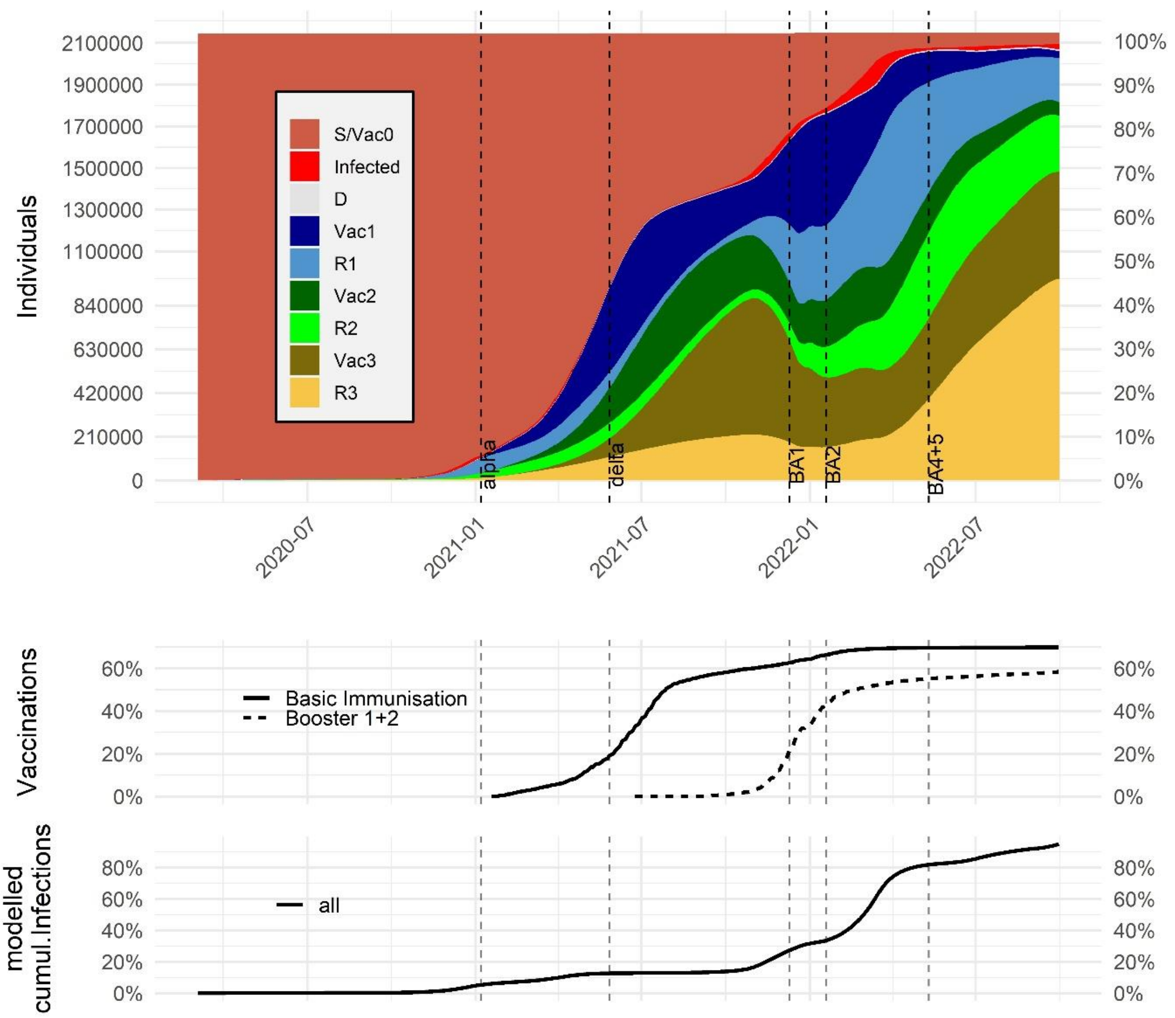
S/Vac0
Infected
D
Vac1
R1
Vac2
R2
Vac3
R3
Individuals
2100000
1900000
1700000
1500000
1300000
1100000
840000
630000
420000
210000
0
100%
90%
80%
70%
60%
50%
40%
30%
20%
10%
0%
alpha
delta
BA1
BA2
BA4+5
2020-07
2021-01
2021-07
2022-01
2022-07
Vaccinations
Basic Immunisation
Booster 1+2
60%
40%
20%
0%
modelled cumul.Infections
all
80%
60%
40%
20%
0%

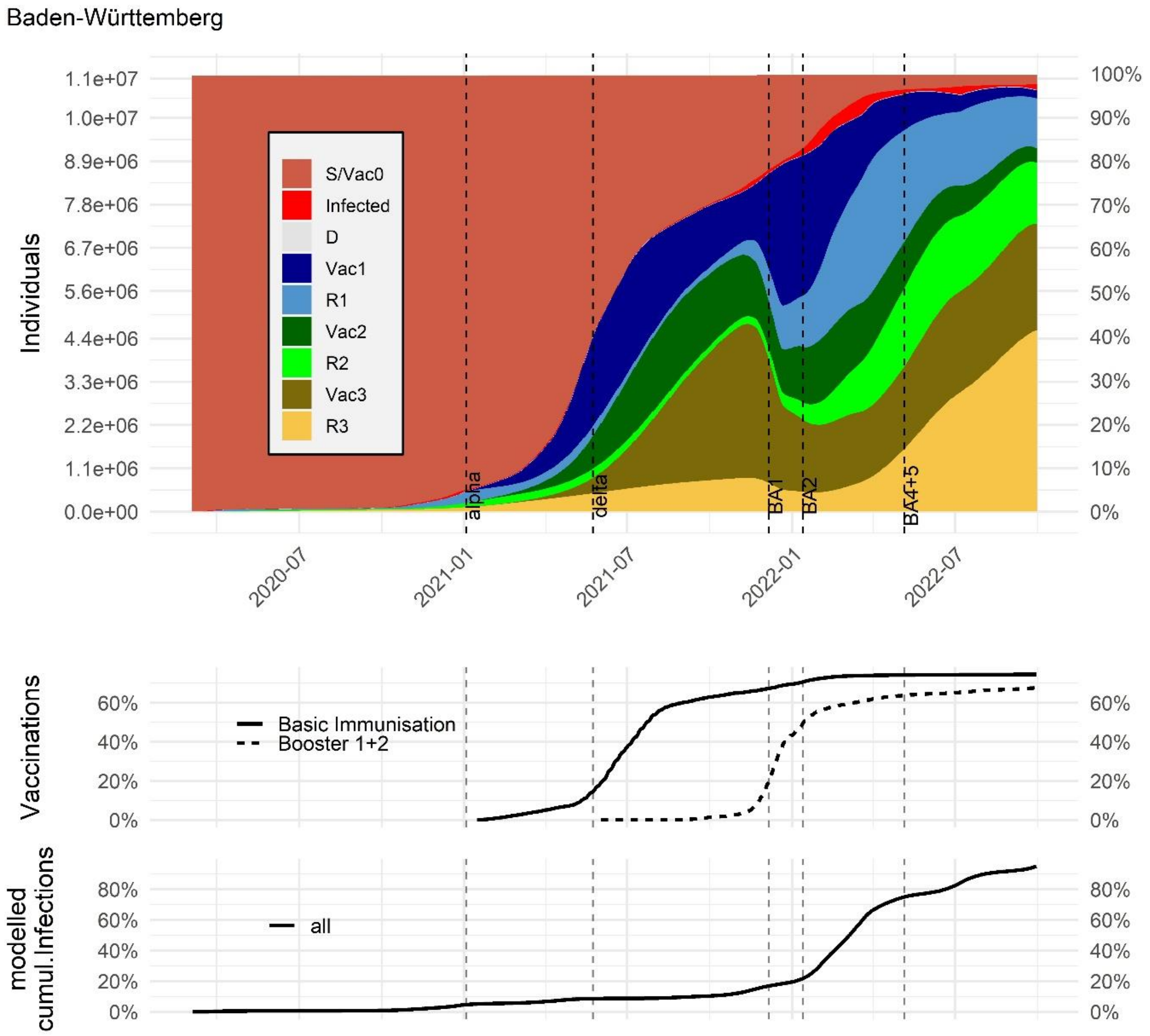
Baden-Württemberg
Individuals
1.1e+07
1.0e+07
8.9e+06
7.8e+06
6.7e+06
5.6e+06
4.4e+06
3.3e+06
2.2e+06
1.1e+06
0.0e+00
100%
90%
80%
70%
60%
50%
40%
30%
20%
10%
0%
S/Vac0
Infected
D
Vac1
R1
Vac2
R2
Vac3
R3
alpha
delta
BA1
BA2
BA4+5
2020-07
2021-01
2021-07
2022-01
2022-07
Vaccinations
60%
40%
20%
0%
Basic Immunisation
Booster 1+2
modelled cumul.Infections
80%
60%
40%
20%
0%
all

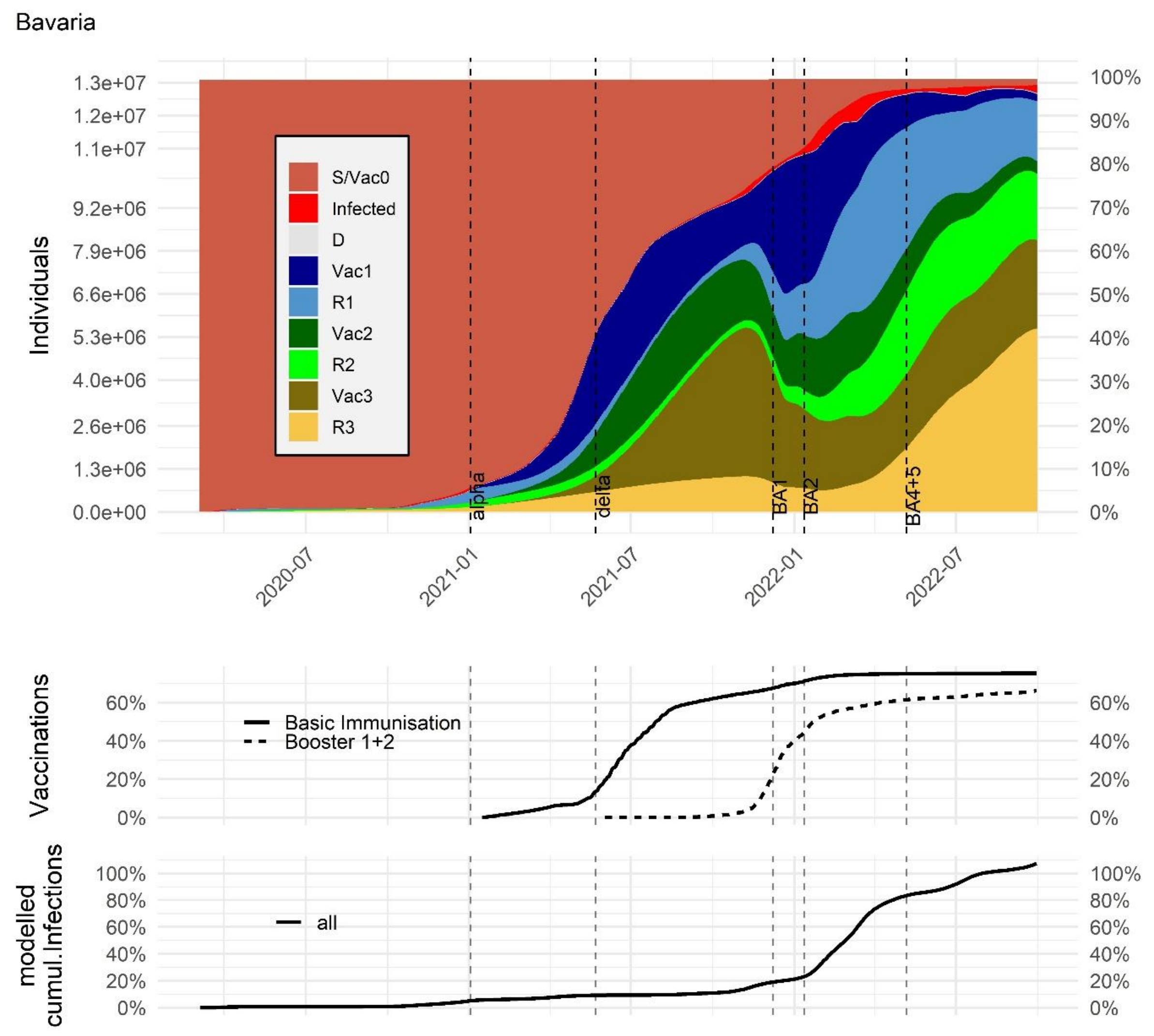
Bavaria
Individuals
S/Vac0
Infected
D
Vac1
R1
Vac2
R2
Vac3
R3
alpha
delta
BA1
BA2
BA4+5
1.3e+07
1.2e+07
1.1e+07
9.2e+06
7.9e+06
6.6e+06
5.3e+06
4.0e+06
2.6e+06
1.3e+06
0.0e+00
100%
90%
80%
70%
60%
50%
40%
30%
20%
10%
0%
2020-07
2021-01
2021-07
2022-01
2022-07
Vaccinations
Basic Immunisation
Booster 1+2
60%
40%
20%
0%
modelled cumul.Infections
all
100%
80%
60%
40%
20%
0%

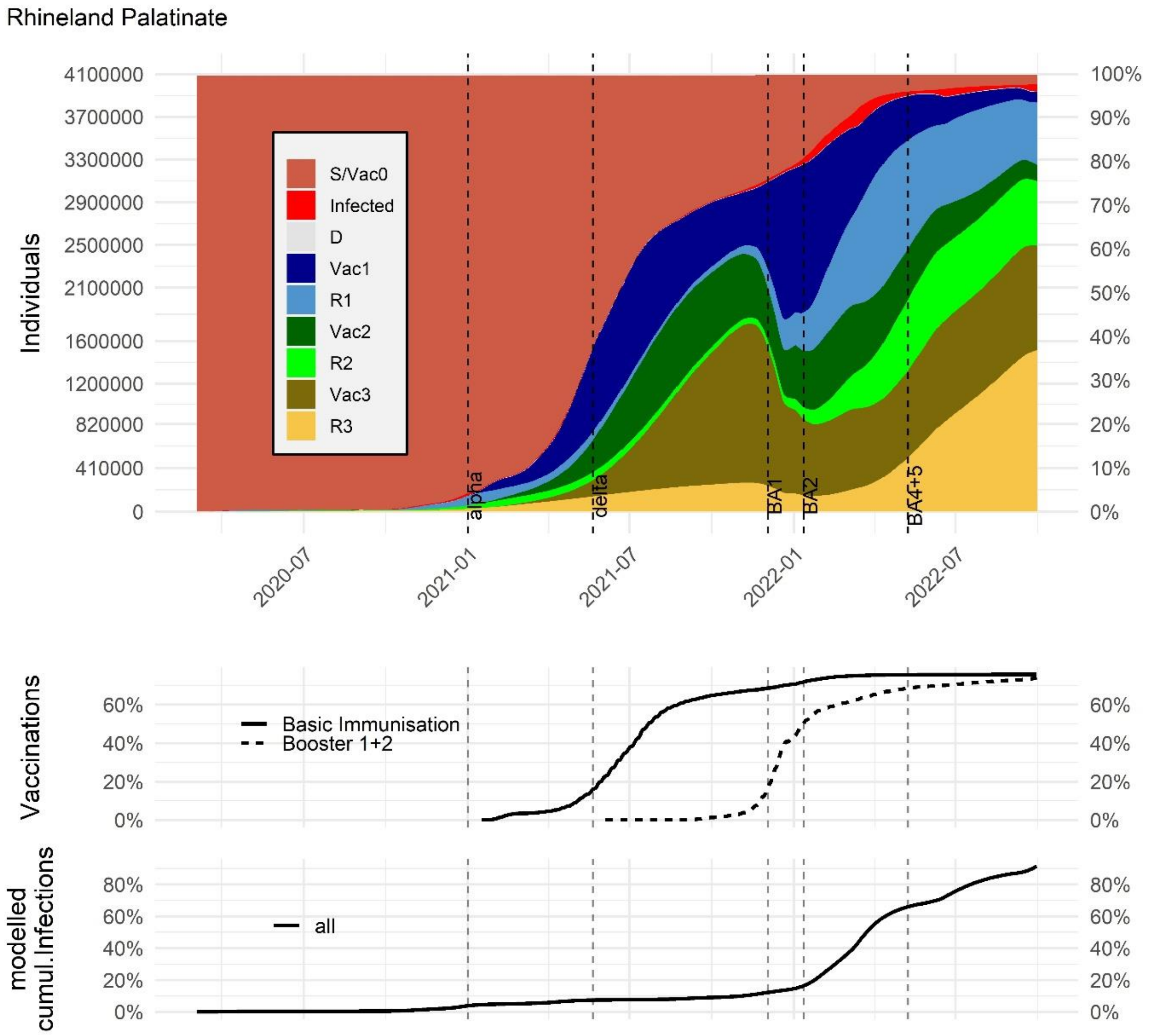

Rhineland Palatinate
Individuals
4100000
3700000
3300000
2900000
2500000
2100000
1600000
1200000
820000
410000
0
100%
90%
80%
70%
60%
50%
40%
30%
20%
10%
0%
S/Vac0
Infected
D
Vac1
R1
Vac2
R2
Vac3
R3
alpha
delta
BA1
BA2
BA4+5
2020-07
2021-01
2021-07
2022-01
2022-07
Vaccinations
60%
40%
20%
0%
Basic Immunisation
Booster 1+2
modelled cumul.Infections
80%
60%
40%
20%
0%
all

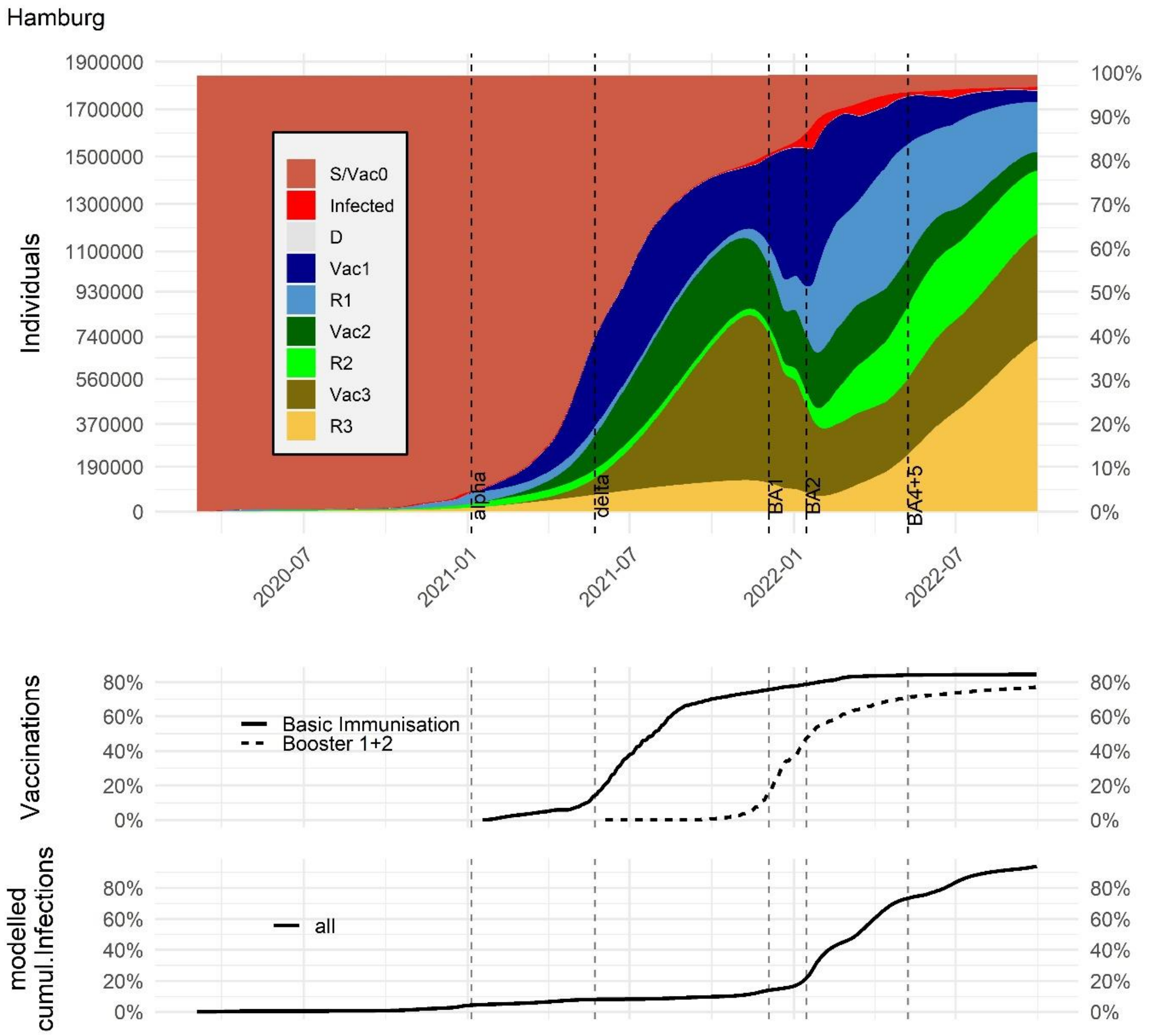

Hamburg
Individuals
1900000
1700000
1500000
1300000
1100000
930000
740000
560000
370000
190000
0
100%
90%
80%
70%
60%
50%
40%
30%
20%
10%
0%
S/Vac0
Infected
D
Vac1
R1
Vac2
R2
Vac3
R3
alpha
delta
BA1
BA2
BA4+5
2020-07
2021-01
2021-07
2022-01
2022-07
Vaccinations
Basic Immunisation
Booster 1+2
80%
60%
40%
20%
0%
modelled cumul.Infections
all

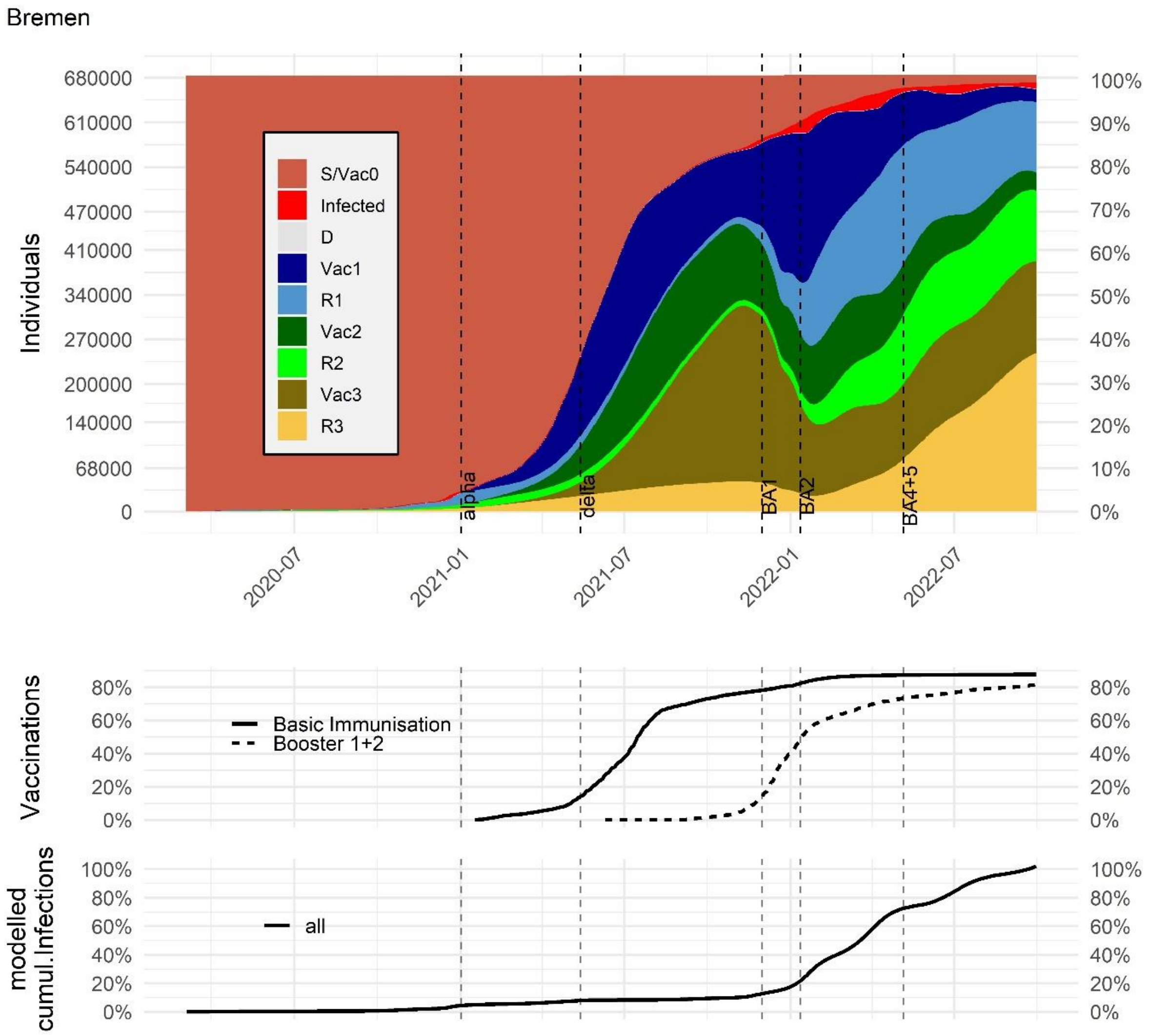
Bremen
Individuals
680000
610000
540000
470000
410000
340000
270000
200000
140000
68000
0
100%
90%
80%
70%
60%
50%
40%
30%
20%
10%
0%
S/Vac0
Infected
D
Vac1
R1
Vac2
R2
Vac3
R3
alpha
delta
BA1
BA2
BA4+5
2020-07
2021-01
2021-07
2022-01
2022-07
Vaccinations
Basic Immunisation
Booster 1+2
modelled cumul.Infections
all

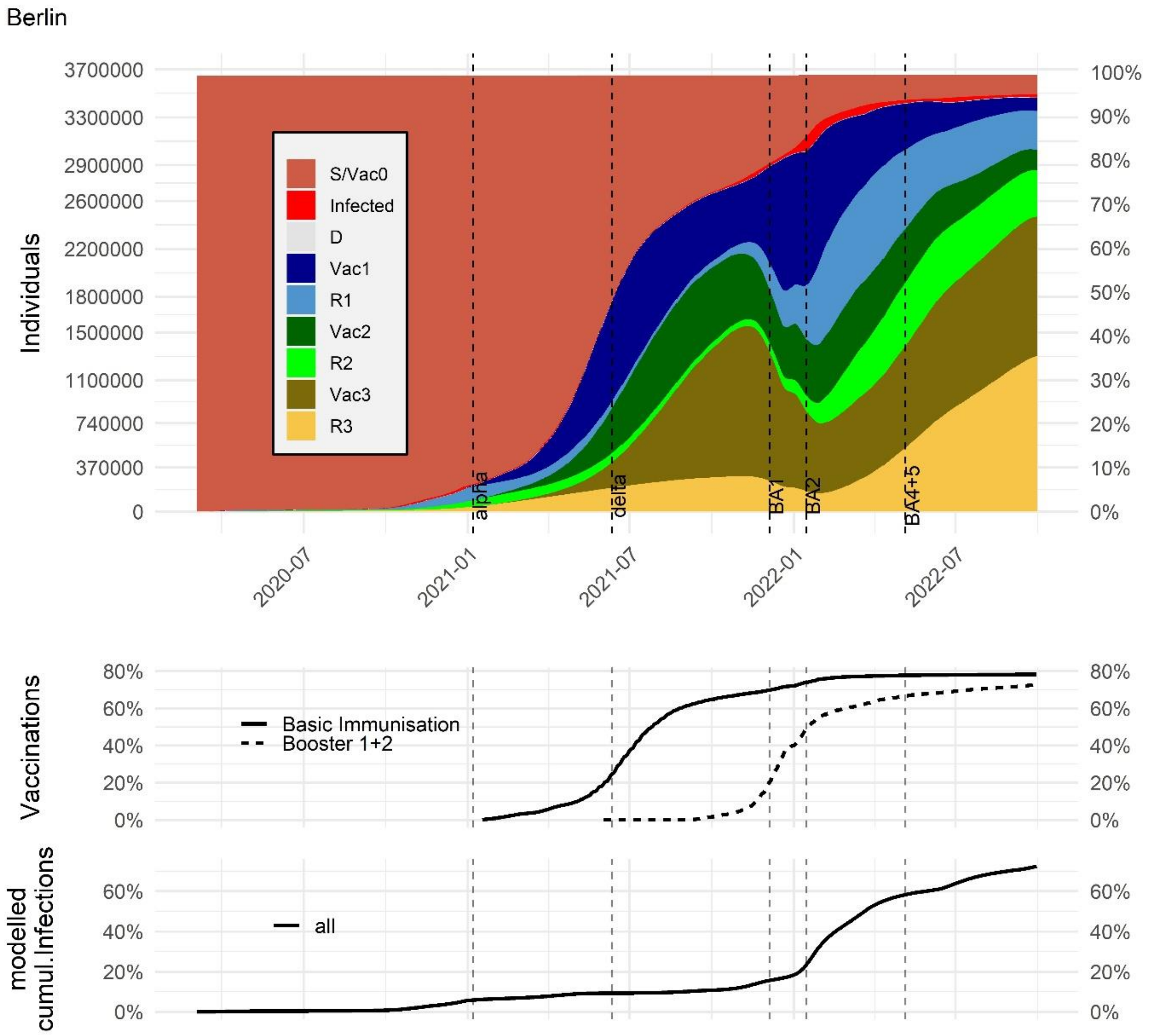
Berlin
Individuals
3700000
3300000
2900000
2600000
2200000
1800000
1500000
1100000
740000
370000
0
100%
90%
80%
70%
60%
50%
40%
30%
20%
10%
0%
S/Vac0
Infected
D
Vac1
R1
Vac2
R2
Vac3
R3
alpha
delta
BA1
BA2
BA4+5
2020-07
2021-01
2021-07
2022-01
2022-07
Vaccinations
80%
60%
40%
20%
0%
Basic Immunisation
Booster 1+2
modelled cumul.Infections
60%
40%
20%
0%
all

Saarland

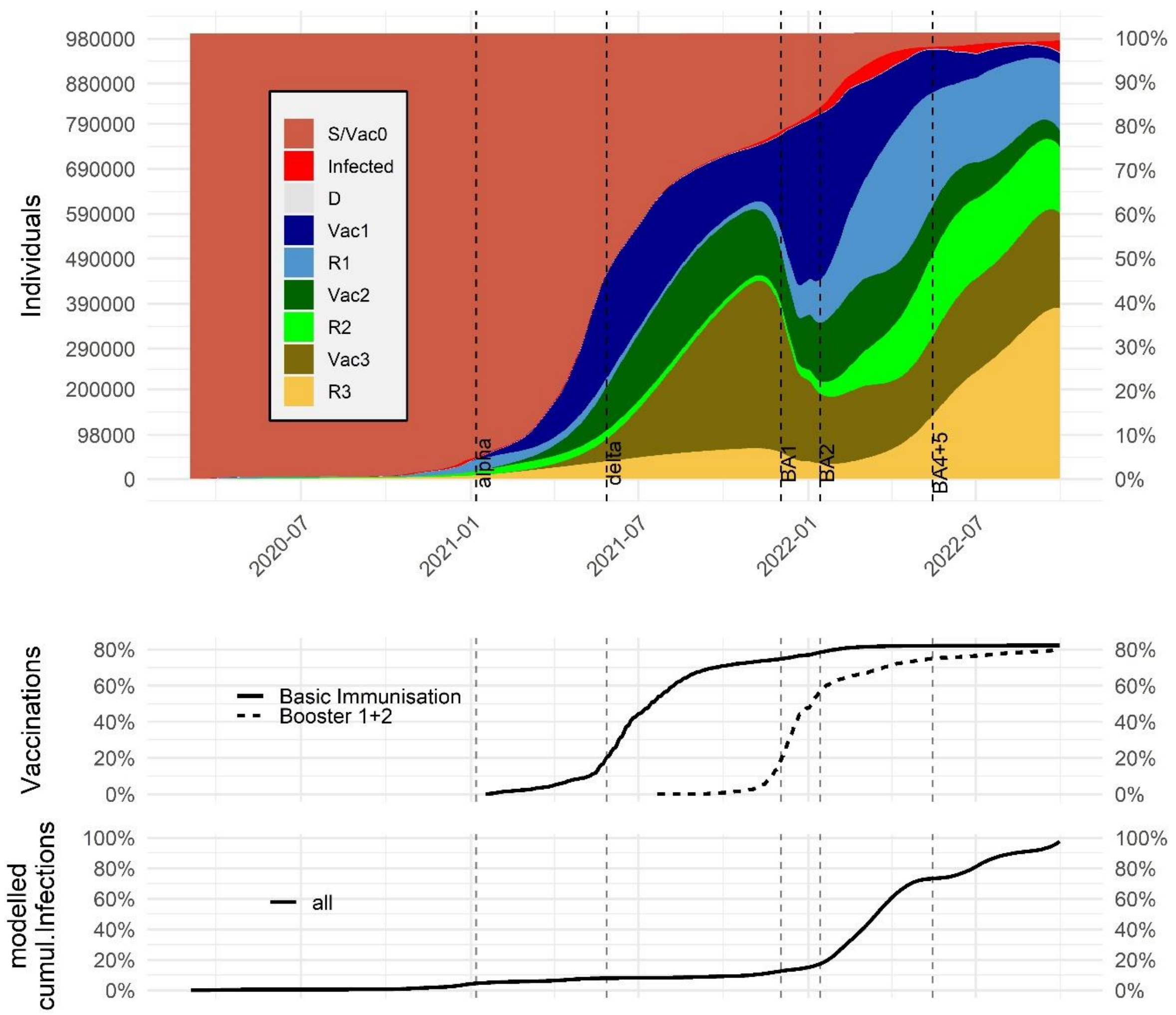
Individuals
980000
880000
790000
690000
590000
490000
390000
290000
200000
98000
0
100%
90%
80%
70%
60%
50%
40%
30%
20%
10%
0%
S/Vac0
Infected
D
Vac1
R1
Vac2
R2
Vac3
R3
alpha
delta
BA1
BA2
BA4+5
2020-07
2021-01
2021-07
2022-01
2022-07
Vaccinations
Basic Immunisation
Booster 1+2
modelled cumul.Infections
all

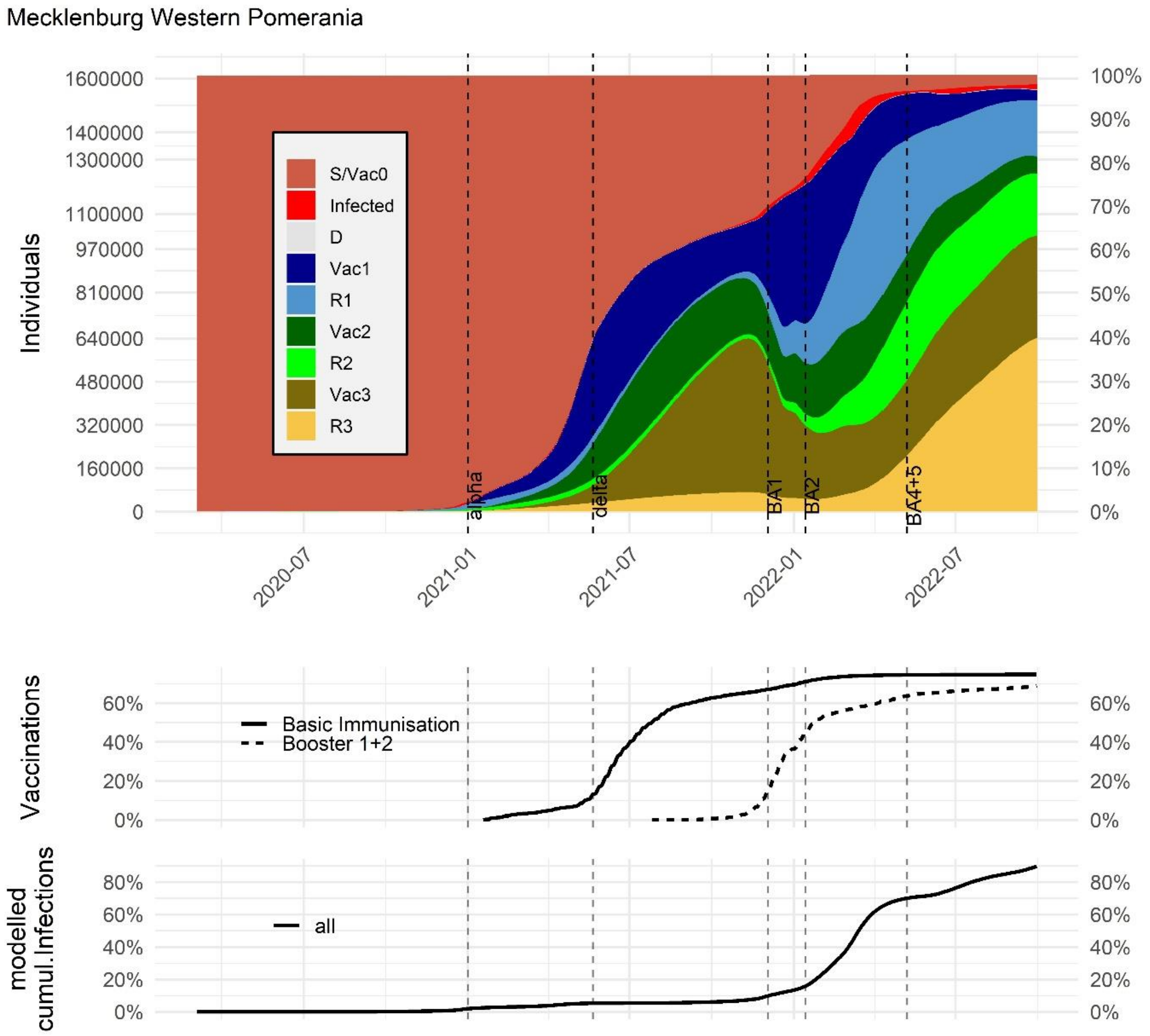
Mecklenburg Western Pomerania
Individuals
1600000
1400000
1300000
1100000
970000
810000
640000
480000
320000
160000
0
100%
90%
80%
70%
60%
50%
40%
30%
20%
10%
0%
S/Vac0
Infected
D
Vac1
R1
Vac2
R2
Vac3
R3
alpha
delta
BA1
BA2
BA4+5
2020-07
2021-01
2021-07
2022-01
2022-07
Vaccinations
Basic Immunisation
Booster 1+2
60%
40%
20%
0%
modelled cumul.Infections
all
80%
60%
40%
20%
0%

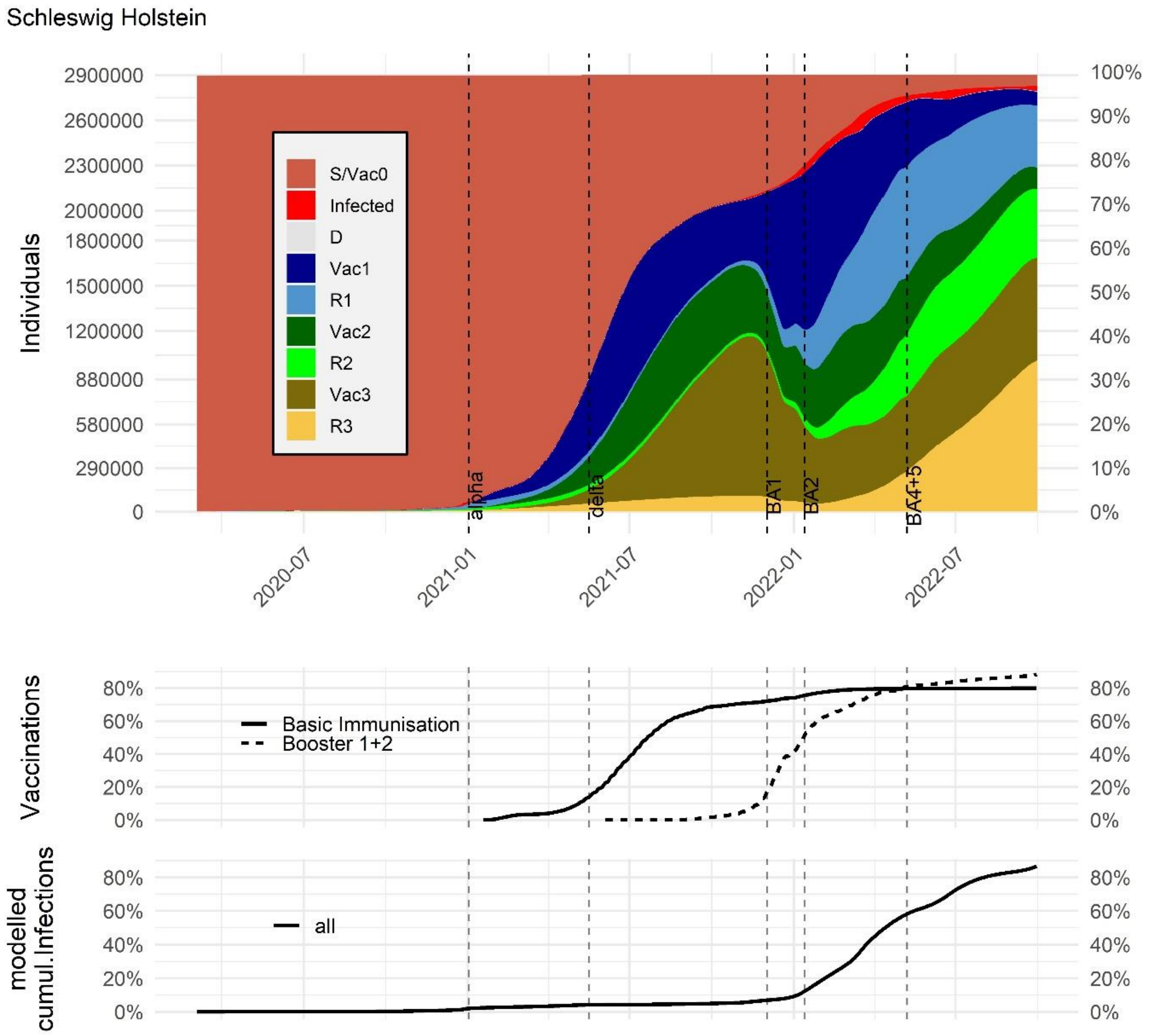
Schleswig Holstein
Individuals
S/Vac0
Infected
D
Vac1
R1
Vac2
R2
Vac3
R3
alpha
delta
BA1
BA2
BA4+5
2020-07
2021-01
2021-07
2022-01
2022-07
Vaccinations
Basic Immunisation
Booster 1+2
modelled cumul.Infections
all

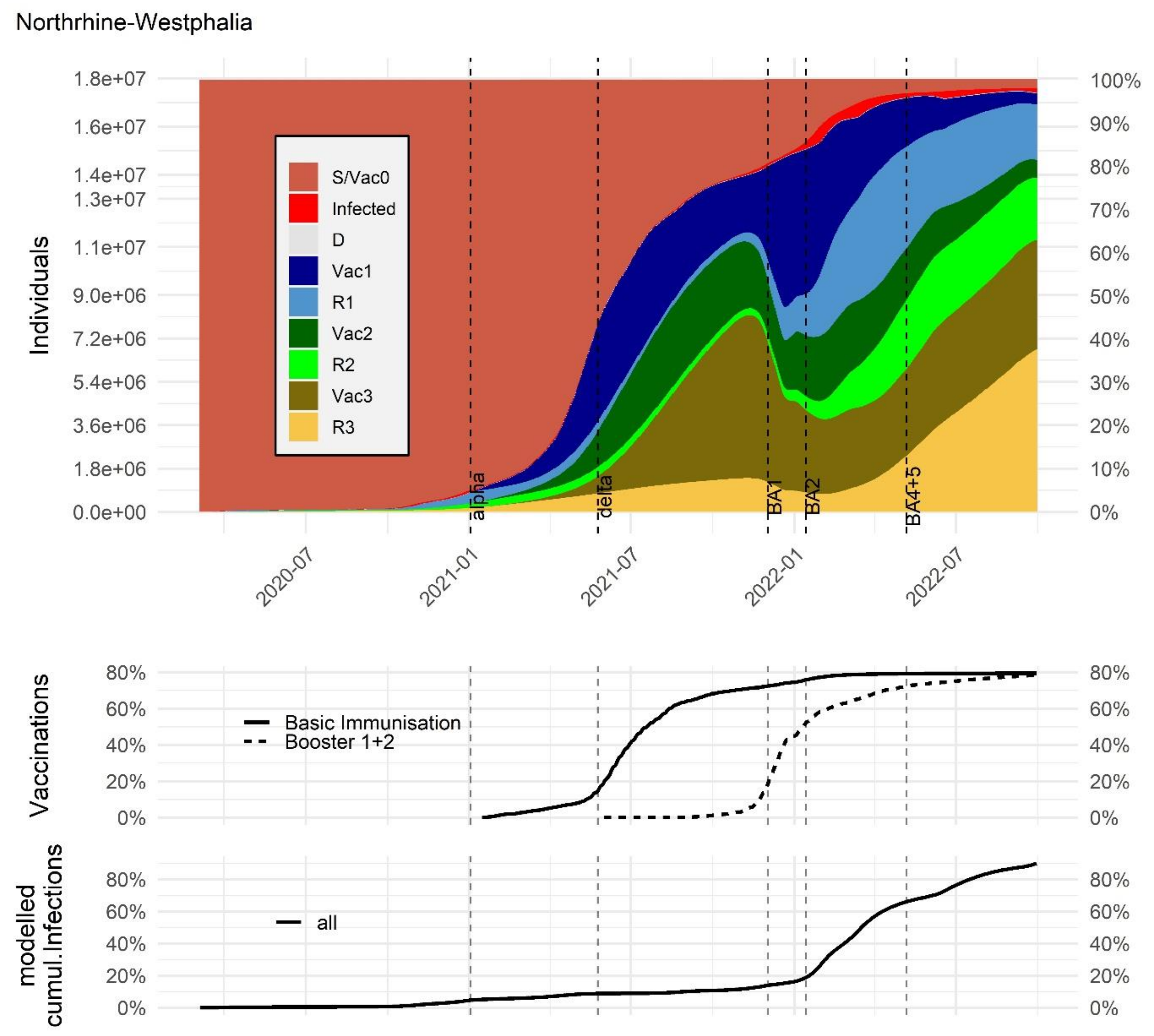
Northrhine-Westphalia
Individuals
1.8e+07
1.6e+07
1.4e+07
1.3e+07
1.1e+07
9.0e+06
7.2e+06
5.4e+06
3.6e+06
1.8e+06
0.0e+00
100%
90%
80%
70%
60%
50%
40%
30%
20%
10%
0%
S/Vac0
Infected
D
Vac1
R1
Vac2
R2
Vac3
R3
alpha
delta
BA1
BA2
BA4+5
2020-07
2021-01
2021-07
2022-01
2022-07
Vaccinations
Basic Immunisation
Booster 1+2
modelled cumul.Infections
all

Hesse

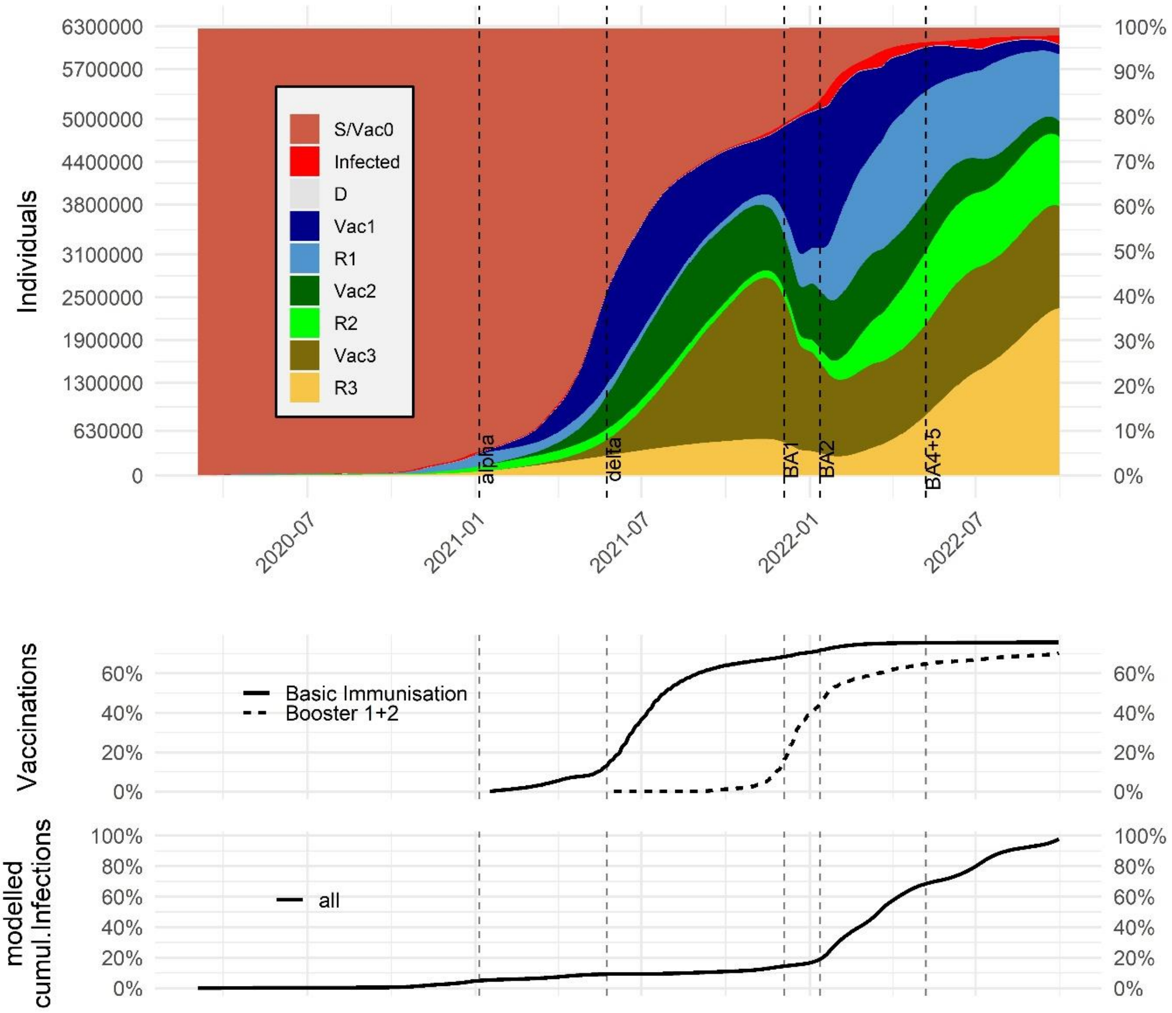

Individuals
6300000
5700000
5000000
4400000
3800000
3100000
2500000
1900000
1300000
630000
0
100%
90%
80%
70%
60%
50%
40%
30%
20%
10%
0%
S/Vac0
Infected
D
Vac1
R1
Vac2
R2
Vac3
R3
alpha
delta
BA1
BA2
BA4+5
2020-07
2021-01
2021-07
2022-01
2022-07
Vaccinations
60%
40%
20%
0%
Basic Immunisation
Booster 1+2
modelled cumul.Infections
100%
80%
60%
40%
20%
0%
all

Supplementary Material N. Modelled age-stratified immune state dynamics of infected subjects and vaccination coverage across German federal states during the COVID-19 pandemic

The figure illustrates temporal patterns of modelled immunity and reported vaccination status for all 16 federal states of Germany, stratified by age groups. The top panel shows the proportion of each age group by immune status. The middle panel tracks vaccination progress by age group, comparing basic immunization (solid lines) versus booster doses 1+2 (dashed lines). The bottom panel displays cumulative infections by age group allowing for reinfection with vertical dashed lines marking variant emergence. The aggregate population trend is shown in magenta ("all").

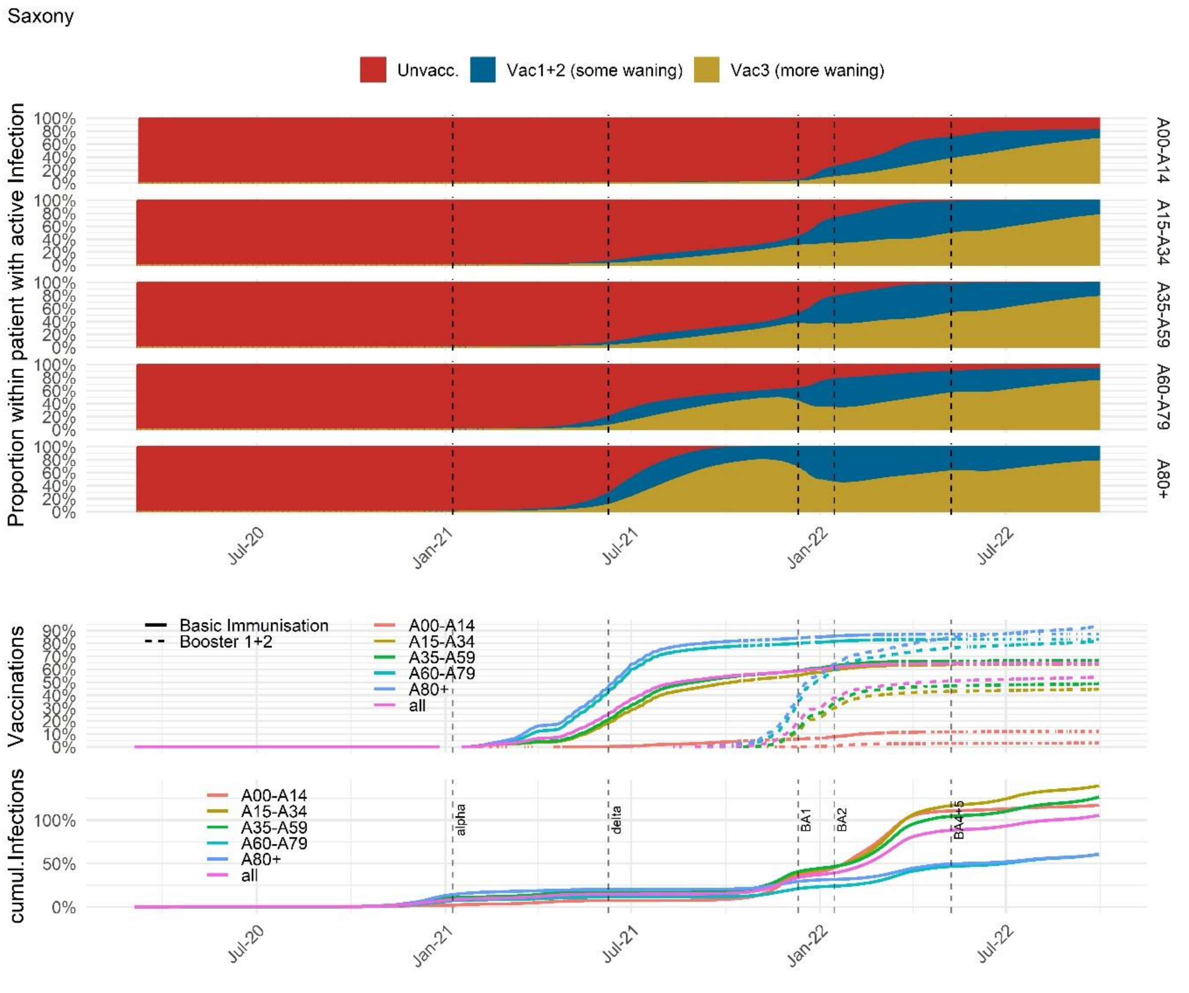

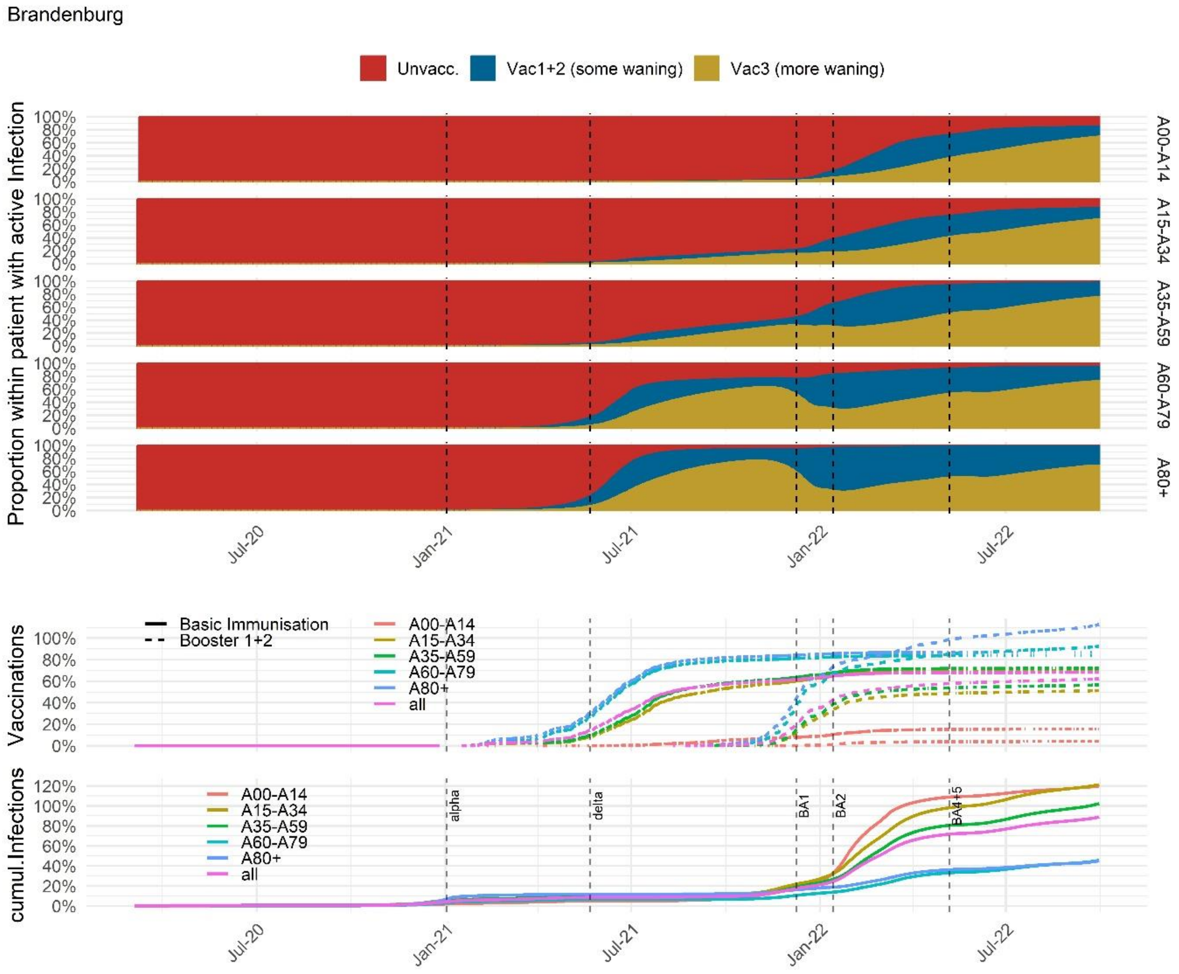
Brandenburg
Unvacc.
Vac1+2 (some waning)
Vac3 (more waning)
Proportion within patient with active Infection
A00-A14
A15-A34
A35-A59
A60-A79
A80+
100%
80%
60%
40%
20%
0%
Jul-20
Jan-21
Jul-21
Jan-22
Jul-22
Basic Immunisation
Booster 1+2
A00-A14
A15-A34
A35-A59
A60-A79
A80+
all
Vaccinations
cumul.Infections
120%
alpha
delta
BA1
BA2
BA4+5

Lower Saxony

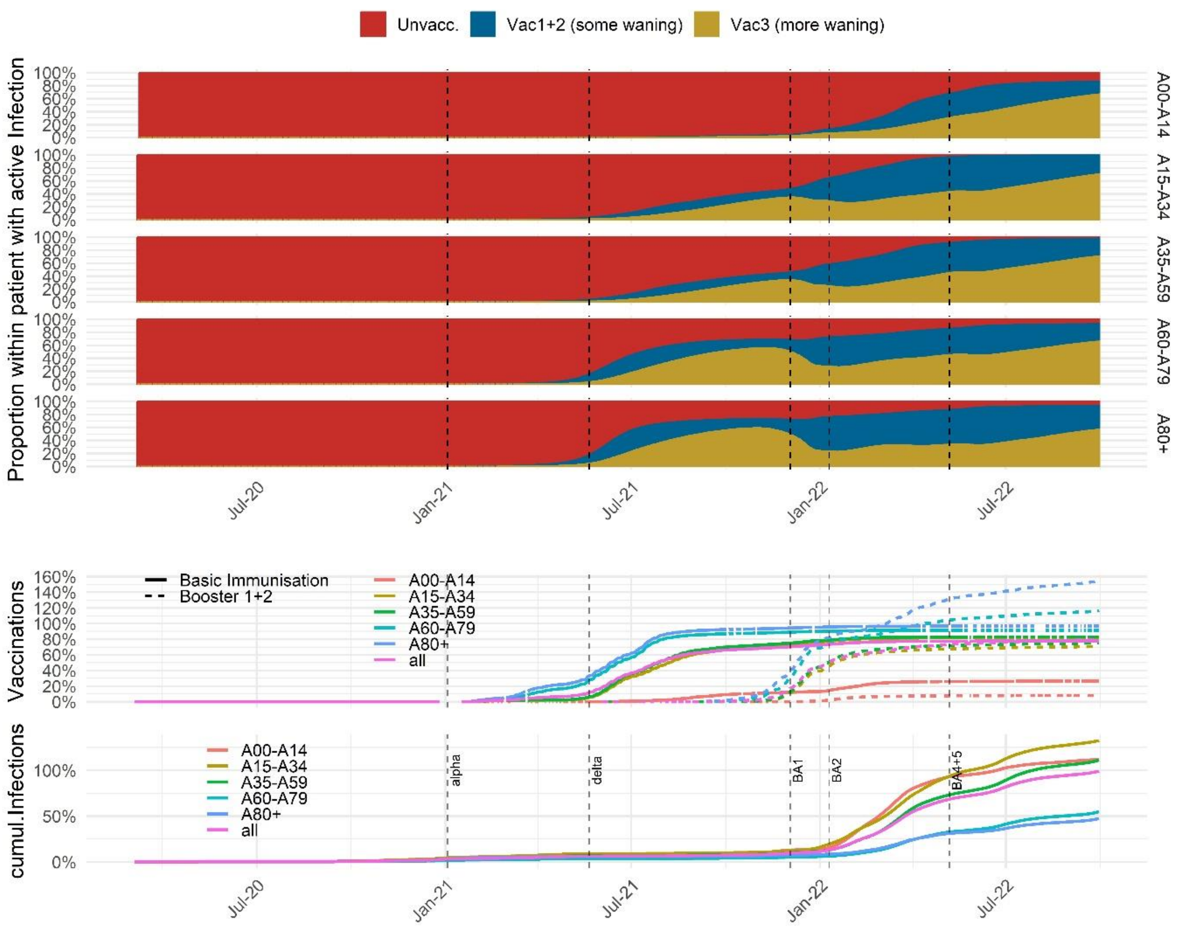

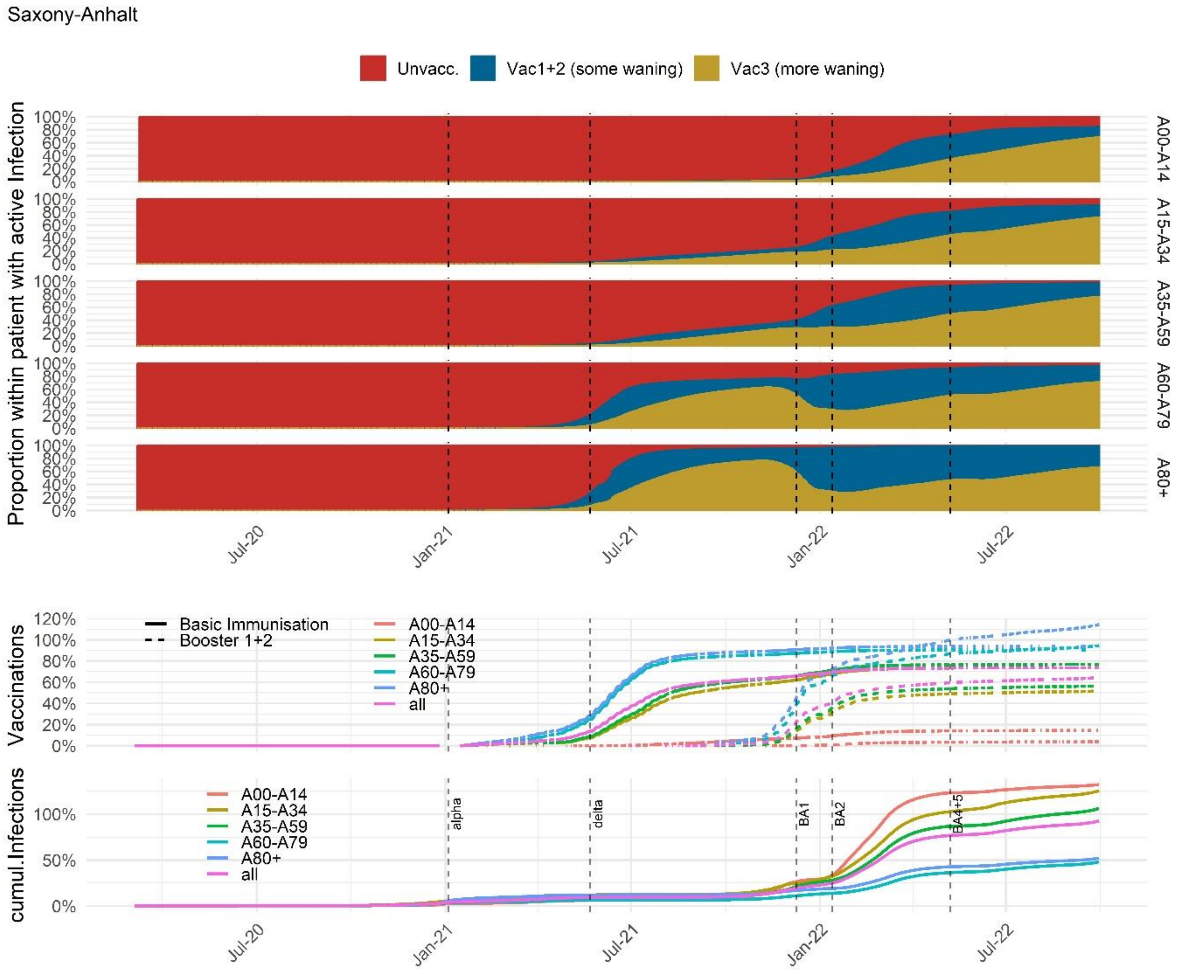
Saxony-Anhalt
Unvacc.
Vac1+2 (some waning)
Vac3 (more waning)
Proportion within patient with active Infection
A00-A14
A15-A34
A35-A59
A60-A79
A80+
Jul-20
Jan-21
Jul-21
Jan-22
Jul-22
Vaccinations
Basic Immunisation
Booster 1+2
all
cumul.Infections
alpha
delta
BA1
BA2
BA4+5

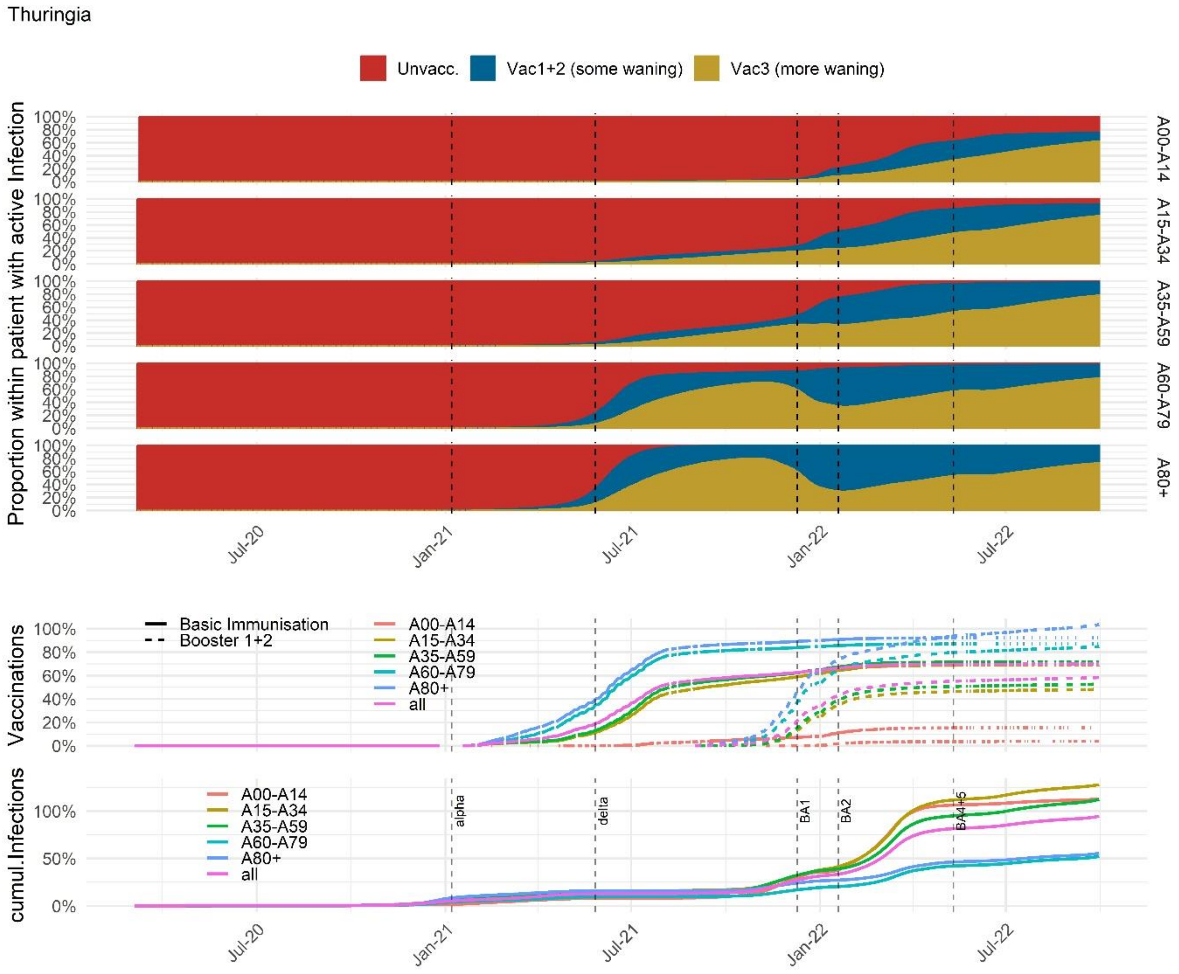
Thuringia
Unvacc.
Vac1+2 (some waning)
Vac3 (more waning)
Proportion within patient with active Infection
100%
80%
60%
40%
20%
0%
A00-A14
A15-A34
A35-A59
A60-A79
A80+
Jul-20
Jan-21
Jul-21
Jan-22
Jul-22
Basic Immunisation
Booster 1+2
A00-A14
A15-A34
A35-A59
A60-A79
A80+
all
Vaccinations
cumul.Infections
100%
50%
0%
alpha
delta
BA1
BA2
BA4+5

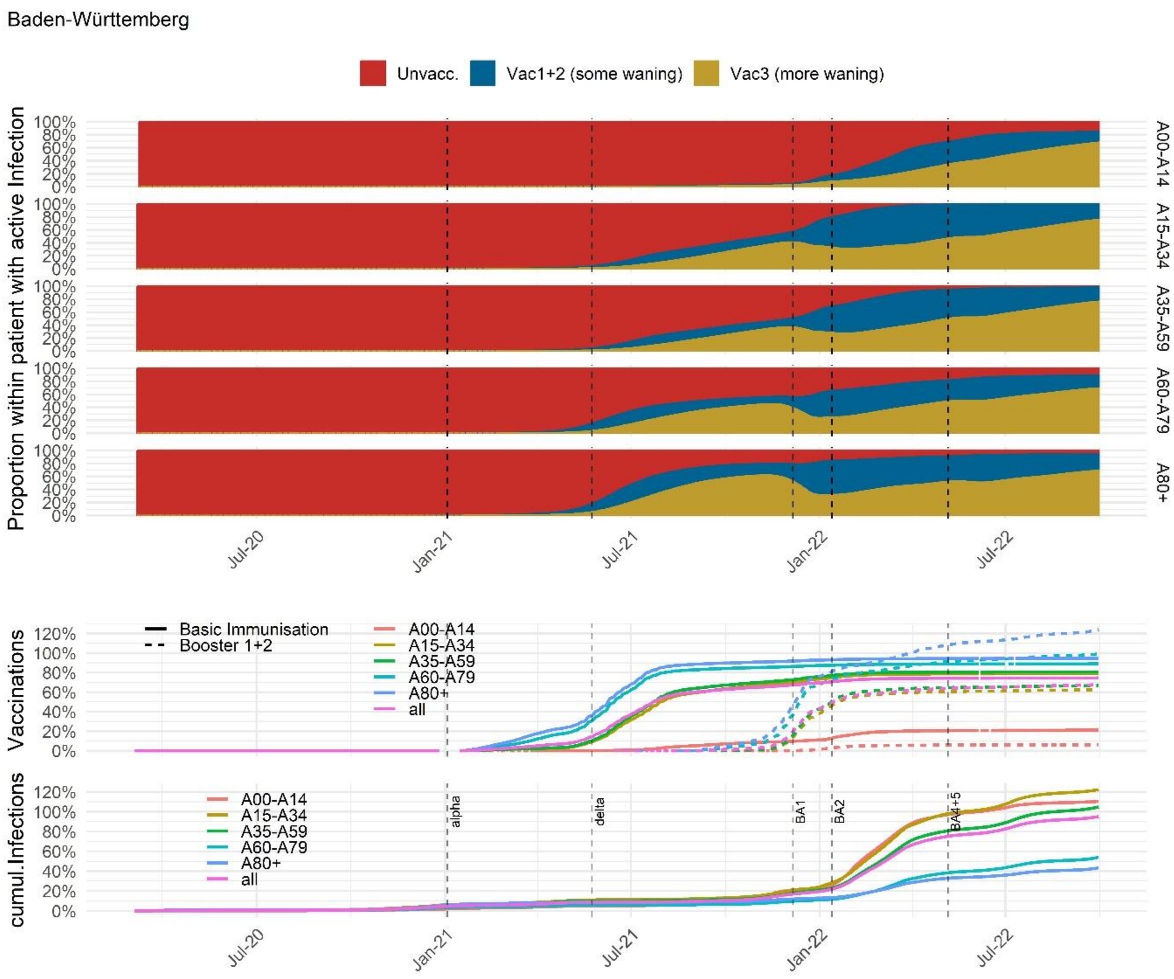
Baden-Württemberg
Unvacc.
Vac1+2 (some waning)
Vac3 (more waning)
Proportion within patient with active Infection
100%
80%
60%
40%
20%
0%
A00-A14
A15-A34
A35-A59
A60-A79
A80+
Jul-20
Jan-21
Jul-21
Jan-22
Jul-22
Vaccinations
120%
100%
80%
60%
40%
20%
0%
Basic Immunisation
Booster 1+2
A00-A14
A15-A34
A35-A59
A60-A79
A80+
all
cumul.Infections
alpha
delta
BA1
BA2
BA4+5

Bavaria

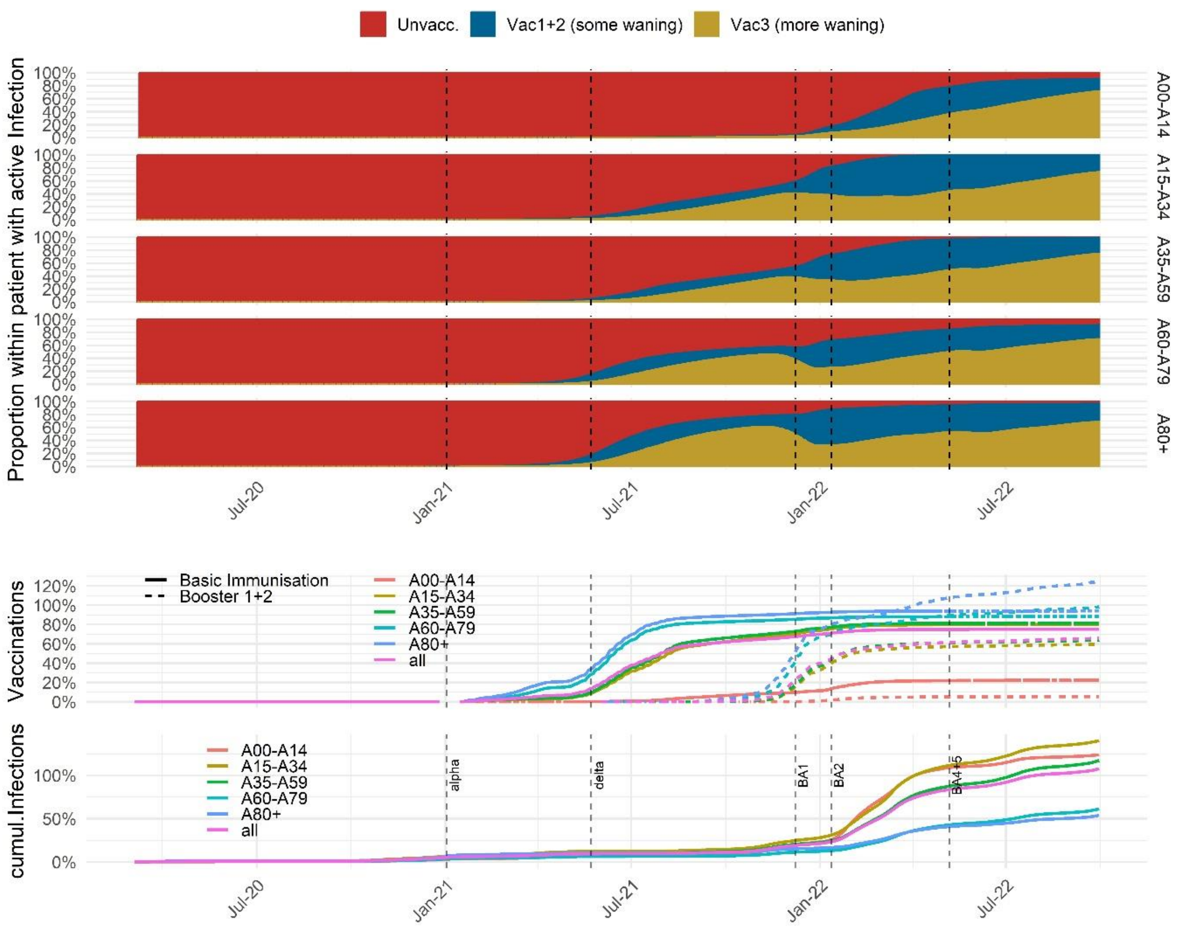
Unvacc.
Vac1+2 (some waning)
Vac3 (more waning)
Proportion within patient with active Infection
100%
80%
60%
40%
20%
0%
A00-A14
A15-A34
A35-A59
A60-A79
A80+
Jul-20
Jan-21
Jul-21
Jan-22
Jul-22
Basic Immunisation
Booster 1+2
A00-A14
A15-A34
A35-A59
A60-A79
A80+
all
Vaccinations
120%
100%
80%
60%
40%
20%
0%
cumul.Infections
100%
50%
0%
alpha
delta
BA1
BA2
BA4+5

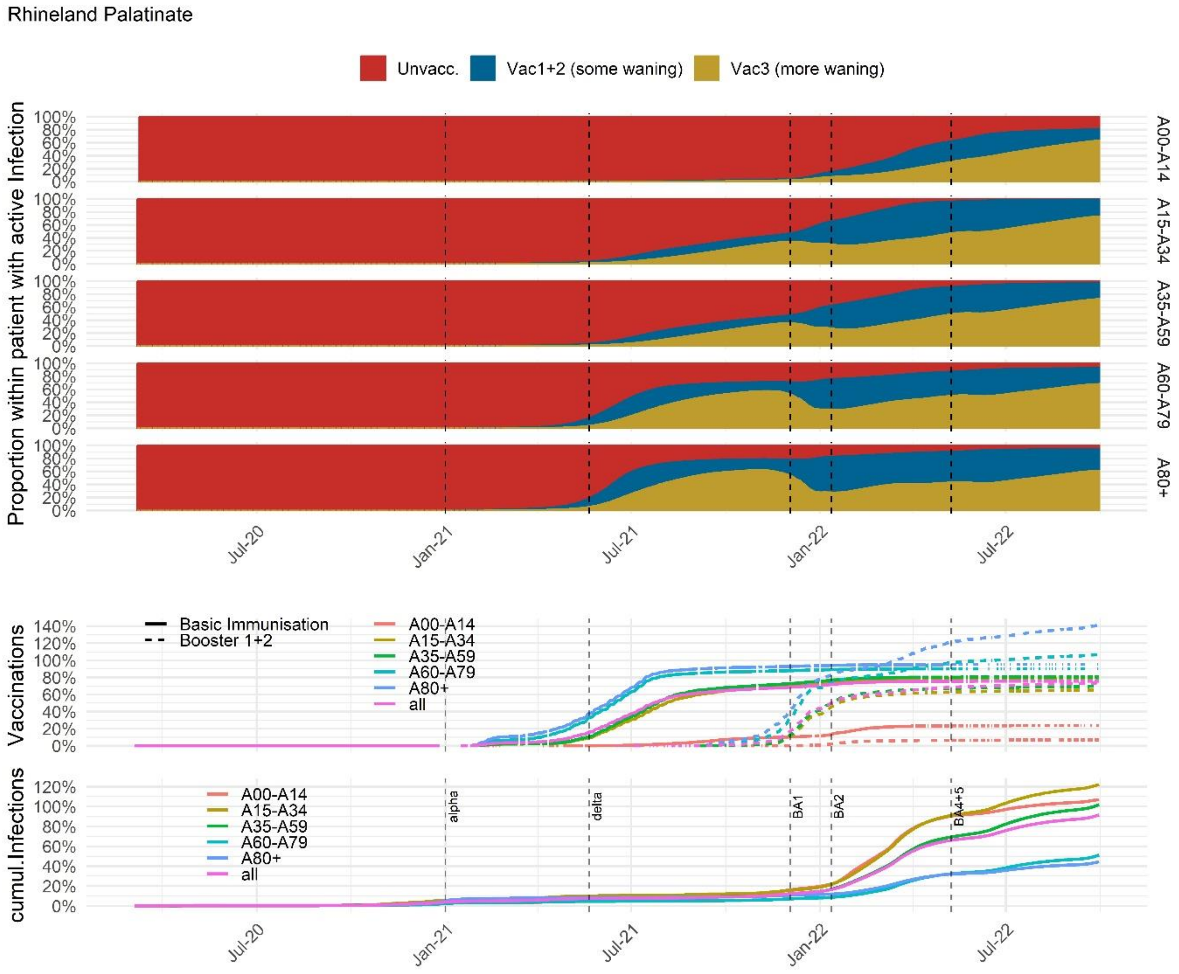
Rhineland Palatinate
Unvacc.
Vac1+2 (some waning)
Vac3 (more waning)
Proportion within patient with active Infection
A00-A14
A15-A34
A35-A59
A60-A79
A80+
Jul-20
Jan-21
Jul-21
Jan-22
Jul-22
Vaccinations
Basic Immunisation
Booster 1+2
all
cumul.Infections
alpha
delta
BA1
BA2
BA4+5

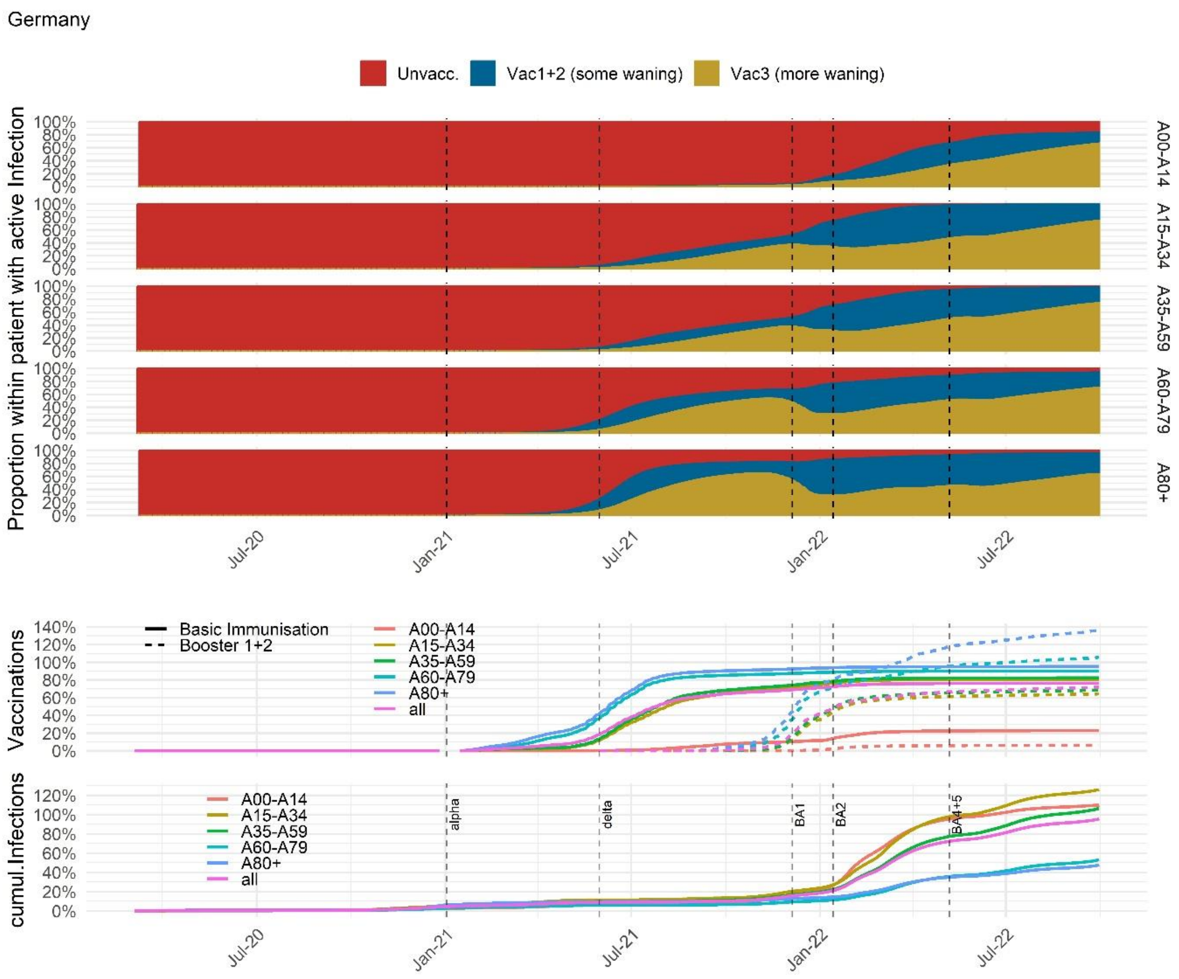
Germany
Unvacc.
Vac1+2 (some waning)
Vac3 (more waning)
Proportion within patient with active Infection
A00-A14
A15-A34
A35-A59
A60-A79
A80+
Jul-20
Jan-21
Jul-21
Jan-22
Jul-22
Vaccinations
Basic Immunisation
Booster 1+2
A00-A14
A15-A34
A35-A59
A60-A79
A80+
all
cumul.Infections
alpha
delta
BA1
BA2
BA4+5

Hamburg

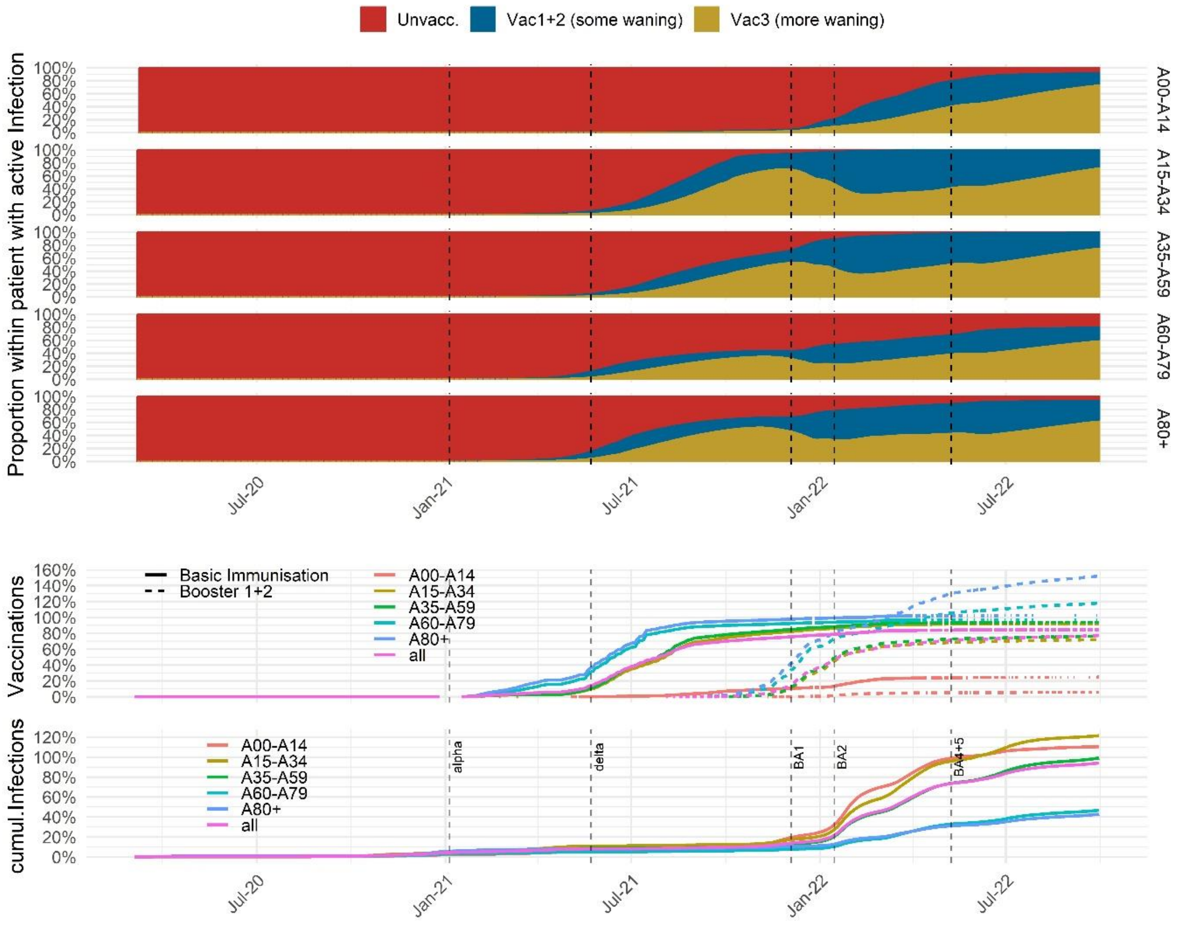
Unvacc.
Vac1+2 (some waning)
Vac3 (more waning)
Proportion within patient with active Infection
A00-A14
A15-A34
A35-A59
A60-A79
A80+
100%
80%
60%
40%
20%
0%
Jul-20
Jan-21
Jul-21
Jan-22
Jul-22
Vaccinations
Basic Immunisation
Booster 1+2
all
160%
140%
120%
cumul.Infections
alpha
delta
BA1
BA2
BA4+5

Bremen

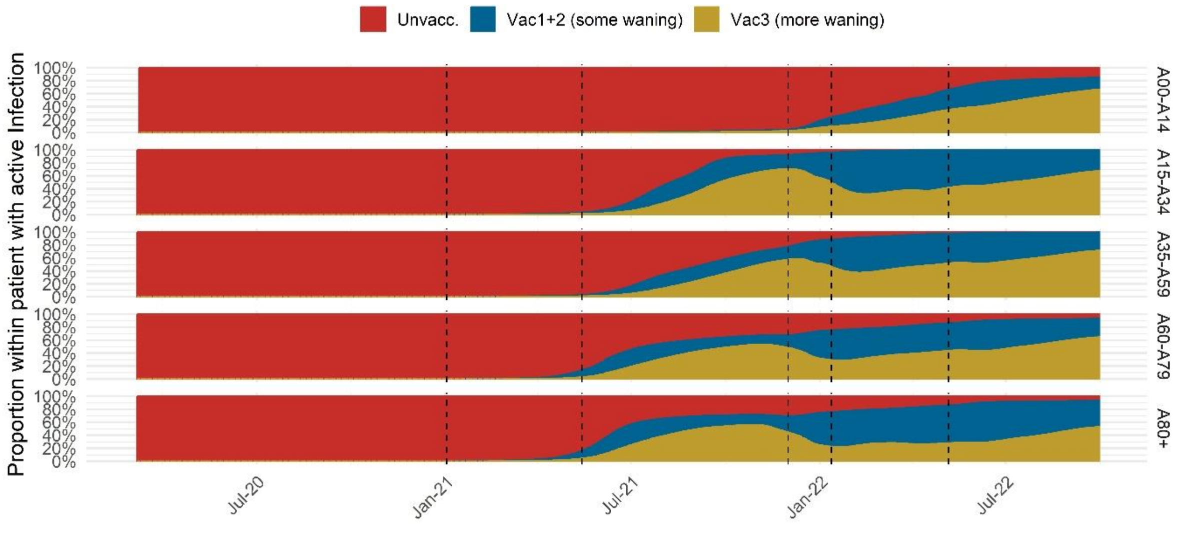
Unvacc.
Vac1+2 (some waning)
Vac3 (more waning)
Proportion within patient with active Infection
100%
80%
60%
40%
20%
0%
A00-A14
A15-A34
A35-A59
A60-A79
A80+
Jul-20
Jan-21
Jul-21
Jan-22
Jul-22

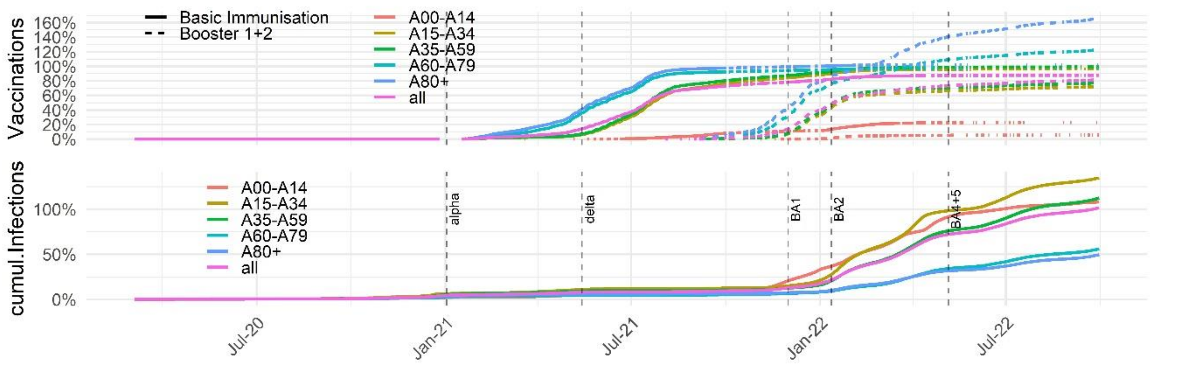
Basic Immunisation
Booster 1+2
A00-A14
A15-A34
A35-A59
A60-A79
A80+
all
Vaccinations
160%
140%
120%
100%
80%
60%
40%
20%
0%
cumul.Infections
100%
50%
0%
alpha
delta
BA1
BA2
BA4+5
Jul-20
Jan-21
Jul-21
Jan-22
Jul-22

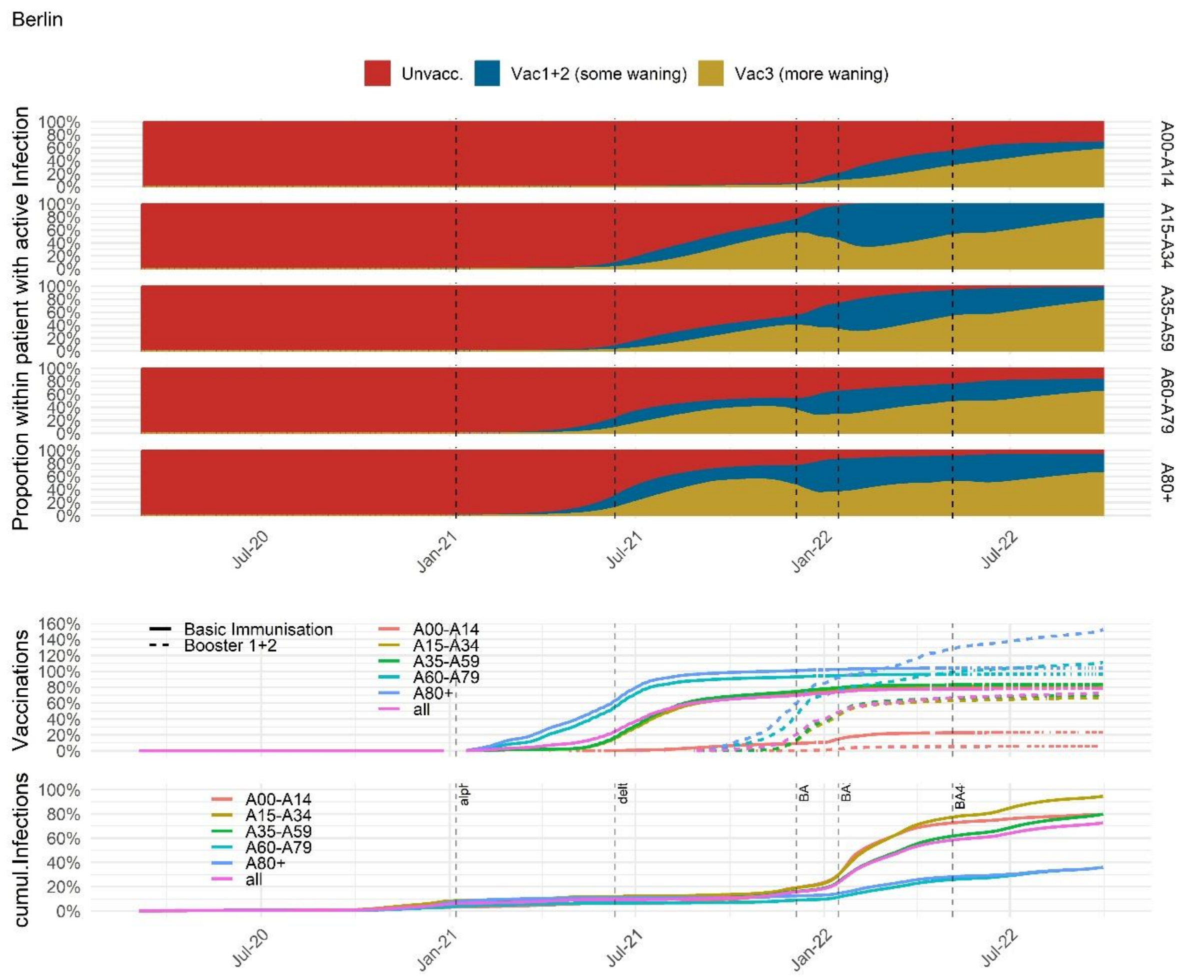
Berlin
Unvacc.
Vac1+2 (some waning)
Vac3 (more waning)
Proportion within patient with active Infection
100%
80%
60%
40%
20%
0%
A00-A14
A15-A34
A35-A59
A60-A79
A80+
Jul-20
Jan-21
Jul-21
Jan-22
Jul-22
Vaccinations
160%
140%
120%
Basic Immunisation
Booster 1+2
all
cumul.Infections
alp
delt
BA
BA4

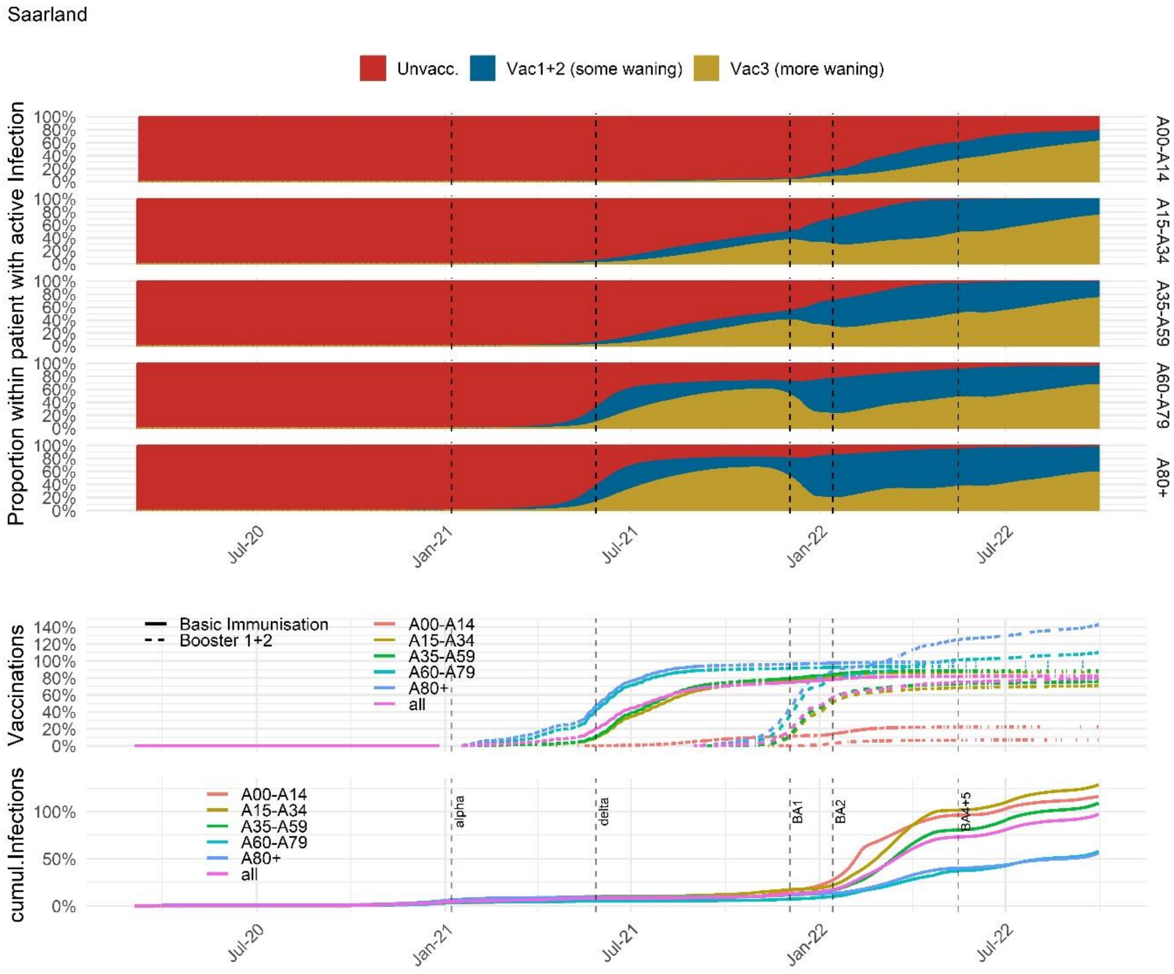
Saarland
Unvacc.
Vac1+2 (some waning)
Vac3 (more waning)
Proportion within patient with active Infection
100%
80%
60%
40%
20%
0%
A00-A14
A15-A34
A35-A59
A60-A79
A80+
Jul-20
Jan-21
Jul-21
Jan-22
Jul-22
Vaccinations
140%
120%
100%
Basic Immunisation
Booster 1+2
A00-A14
A15-A34
A35-A59
A60-A79
A80+
all
cumul.Infections
50%
alpha
delta
BA1
BA2
BA4+5

Mecklenburg Western Pomerania

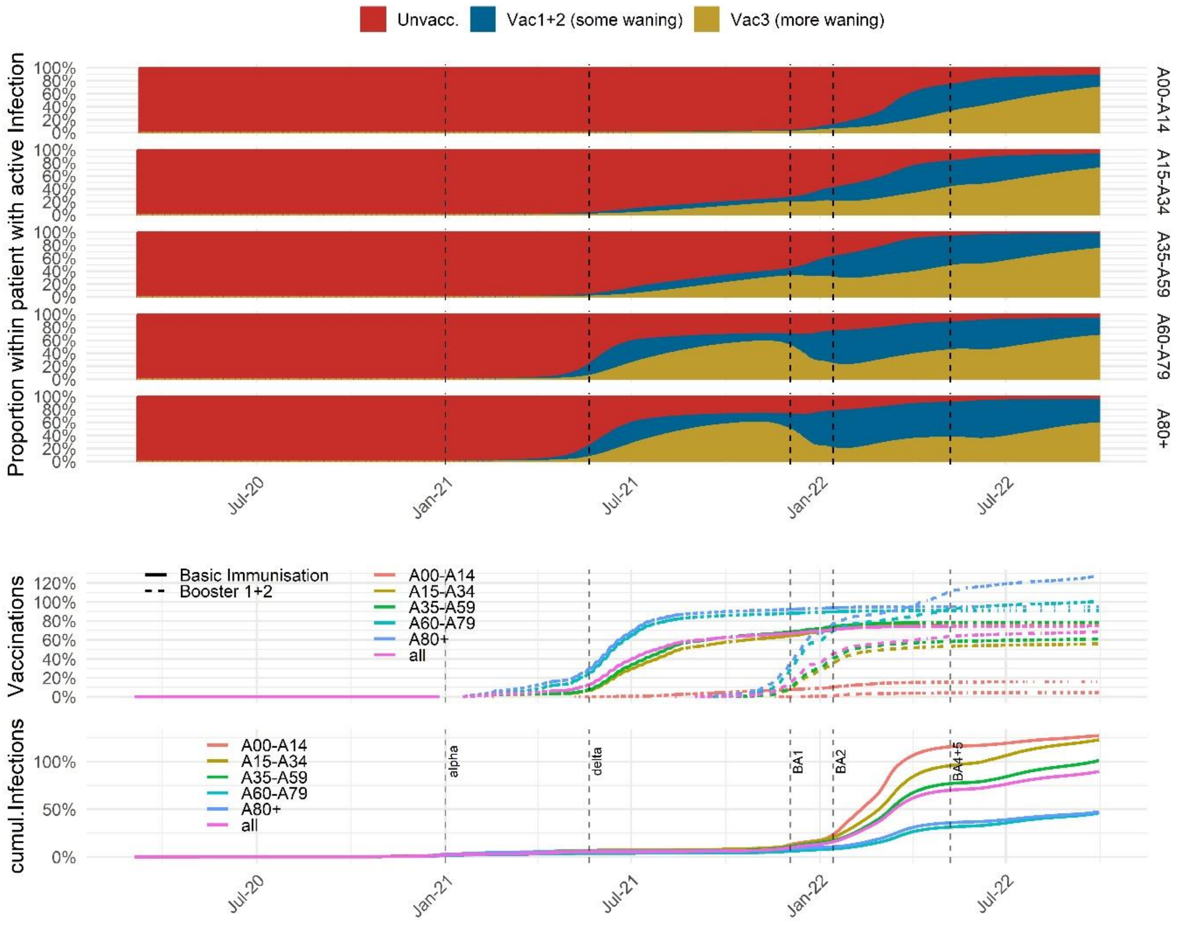

Schleswig Holstein

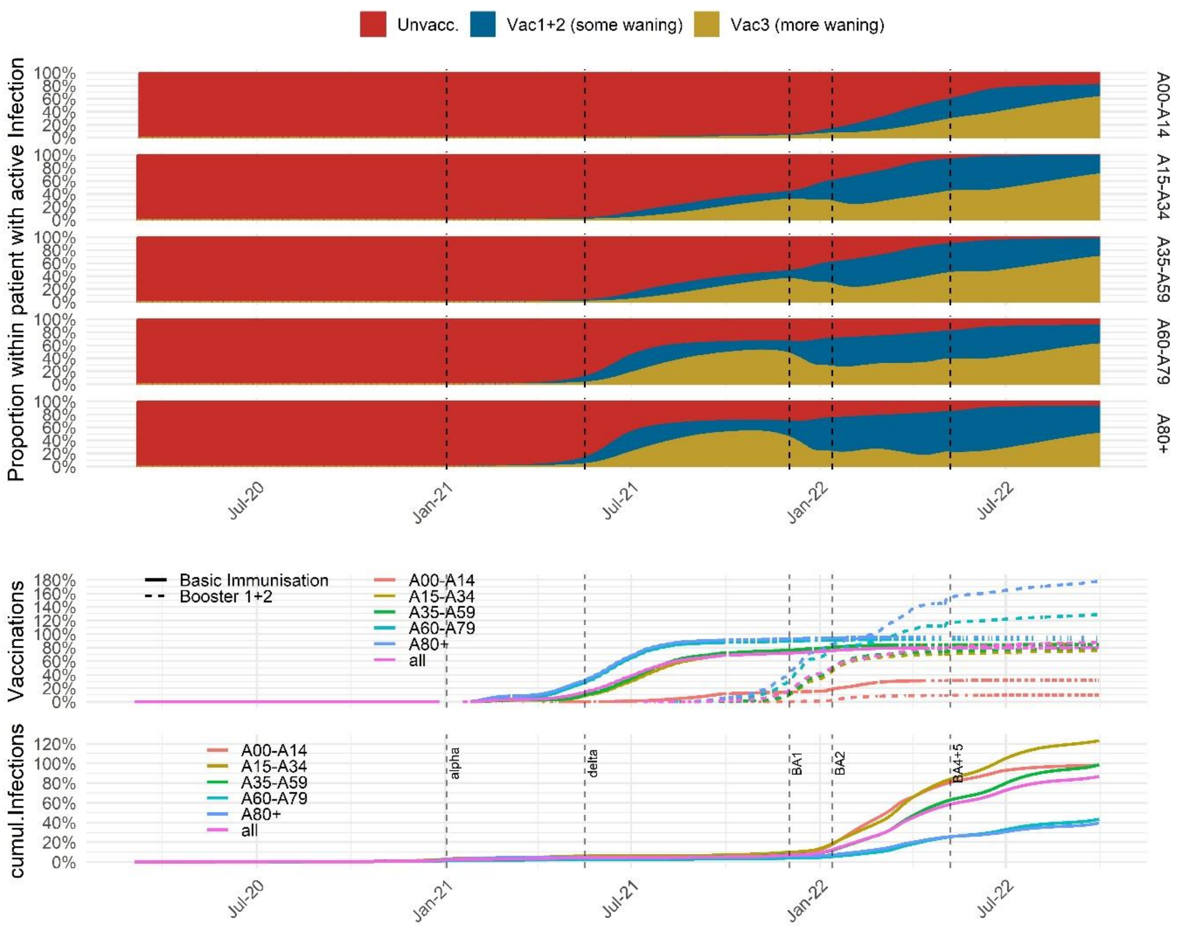

Northrhine-Westphalia

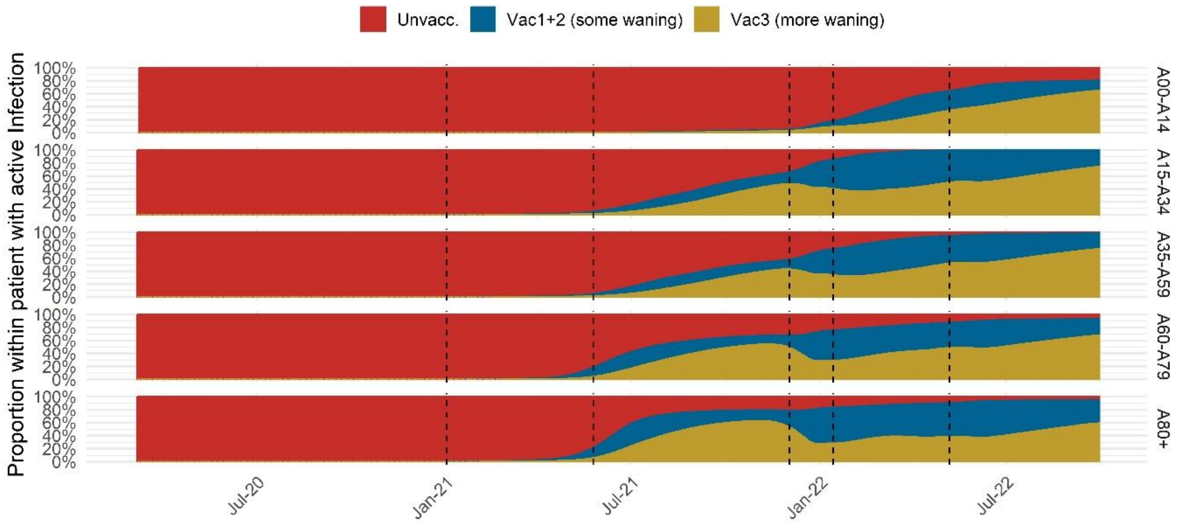

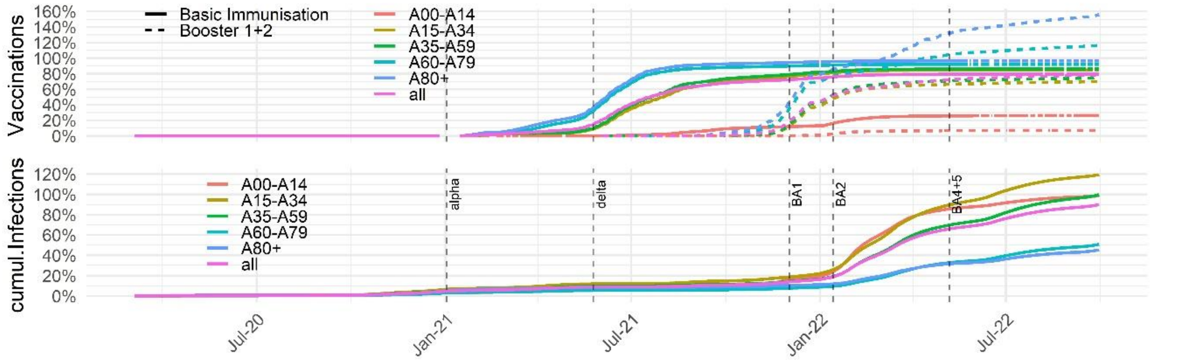

Hesse

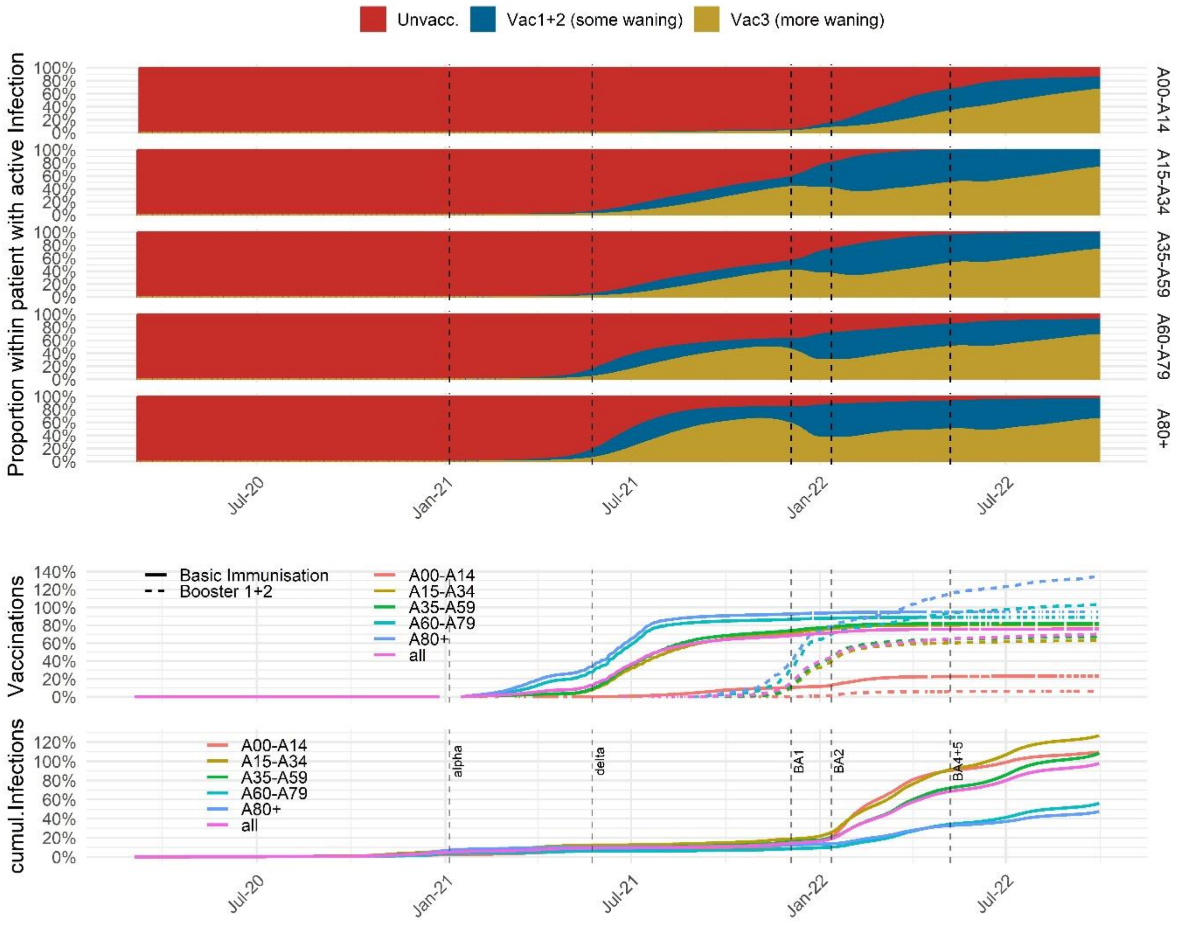

Unvacc.
Vac1+2 (some waning)
Vac3 (more waning)
Proportion within patient with active Infection
100%
80%
60%
40%
20%
0%
A00-A14
A15-A34
A35-A59
A60-A79
A80+
Jul-20
Jan-21
Jul-21
Jan-22
Jul-22
Vaccinations
140%
120%
Basic Immunisation
Booster 1+2
A00-A14
A15-A34
A35-A59
A60-A79
A80+
all
cumul.Infections
alpha
delta
BA1
BA2
BA4+5

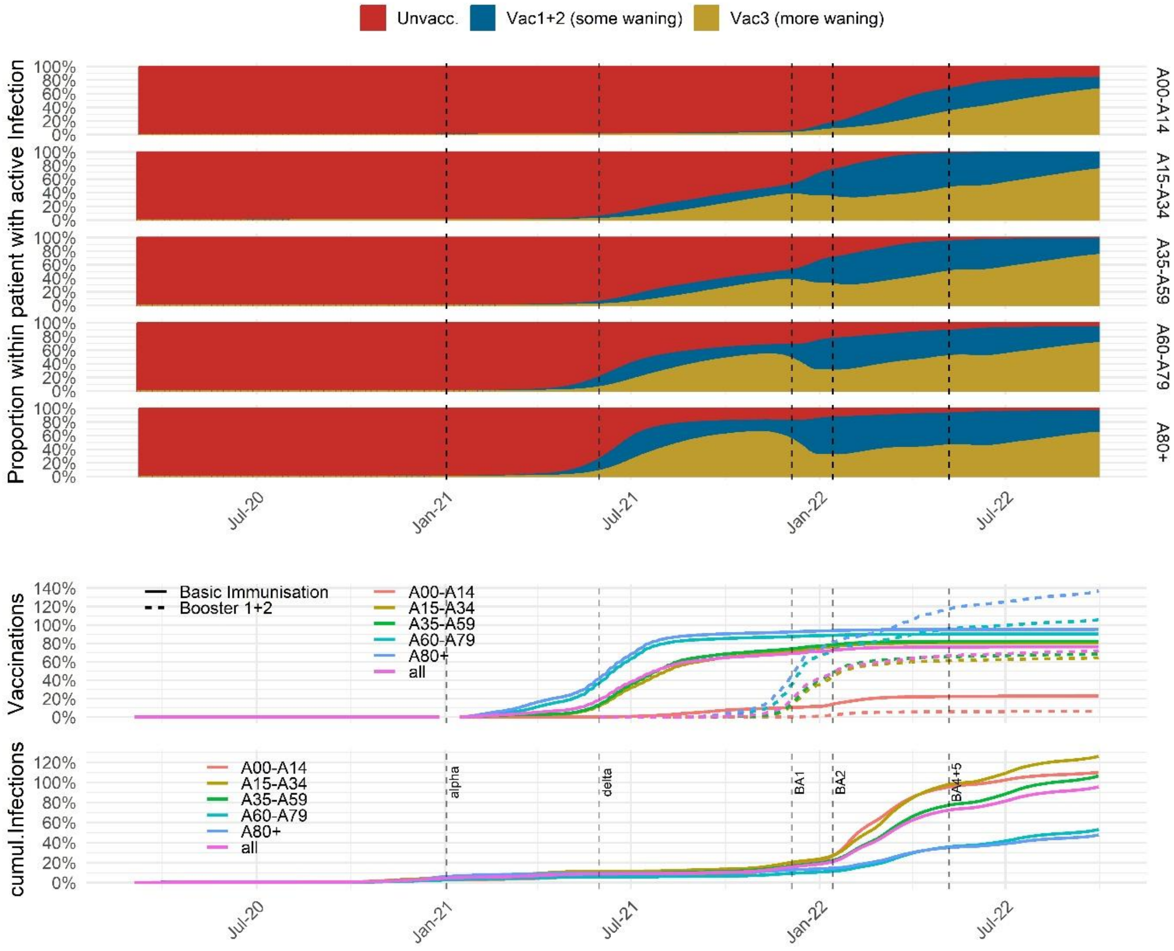

Unvacc.
Vac1+2 (some waning)
Vac3 (more waning)
Proportion within patient with active Infection
100%
80%
60%
40%
20%
0%
A00-A14
A15-A34
A35-A59
A60-A79
A80+
Jul-20
Jan-21
Jul-21
Jan-22
Jul-22
Vaccinations
140%
120%
Basic Immunisation
Booster 1+2
A00-A14
A15-A34
A35-A59
A60-A79
A80+
all
cumul.Infections
alpha
delta
BA1
BA2
BA4+5

Supplementary Material O. Development of age-groups over the course of the pandemic

Based on census data, we calculated the absolute (left) and relative (right) size of the age-groups considered in our model for Germany. Relative size of age-groups remains approximately constant justifying that birth rates, non-COVID-19 related deaths and aging is not considered in the current version of the model.

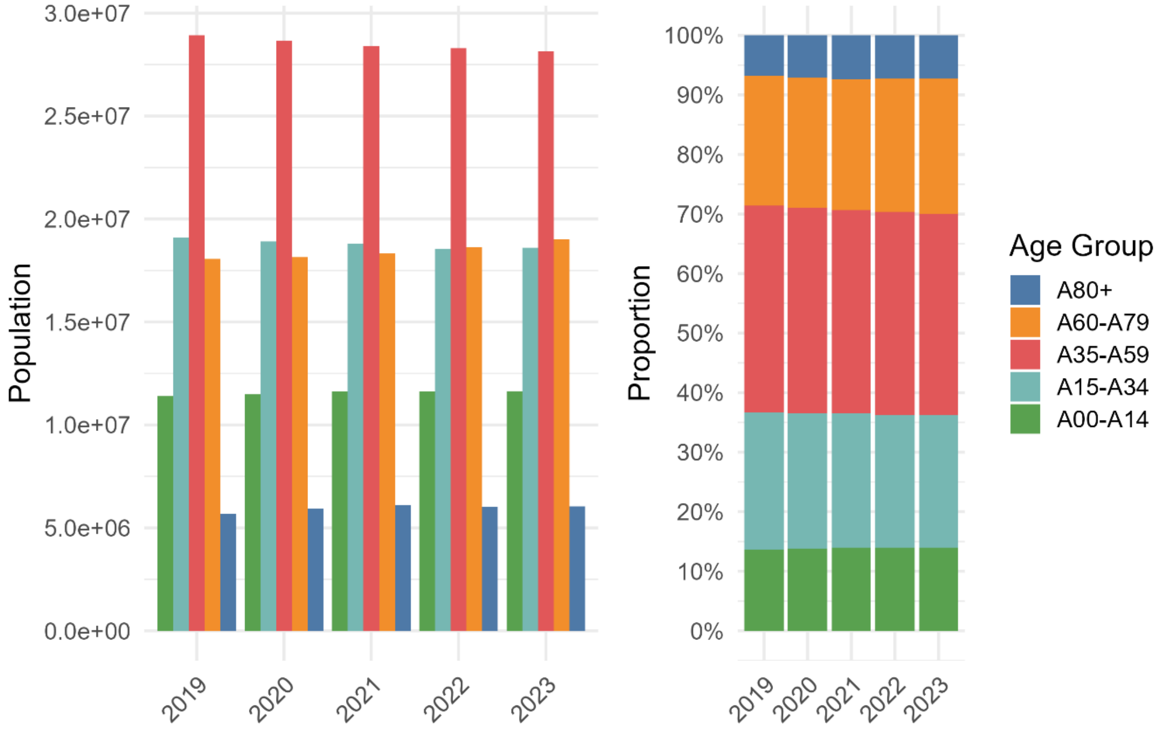

References

1. Mossong J, Hens N, Jit M, Beutels P, Auranen K, Mikolajczyk R, et al. Social contacts and mixing patterns relevant to the spread of infectious diseases. PLoS Med. 2008; 5:e74. doi: 10.1371/journal.pmed.0050074 PMID: 18366252.
2. Der Heiden M an, Buchholz U. Modellierung von Beispielszenarien der SARS-CoV-2-Ausbreitung und Schwere in Deutschland. Robert Koch-Institut; 2020.
3. Nishiura H, Linton NM, Akhmetzhanov AR. Serial interval of novel coronavirus (COVID-19) infections. Int J Infect Dis. 2020; 93:284–6. Epub 2020/03/04. doi: 10.1016/j.ijid.2020.02.060 PMID: 32145466.
4. Tindale LC, Stockdale JE, Coombe M, Garlock ES, Lau WYV, Saraswat M, et al. Evidence for transmission of COVID-19 prior to symptom onset. Elife. 2020; 9. Epub 2020/06/22. doi: 10.7554/eLife.57149 PMID: 32568070.
5. Böhmer MM, Buchholz U, Corman VM, Hoch M, Katz K, Marosevic DV, et al. Investigation of a COVID-19 outbreak in Germany resulting from a single travel-associated primary case: a case series. The Lancet Infectious Diseases. 2020; 20:920–8. doi: 10.1016/S1473-3099(20)30314-5.
6. Ganyani T, Kremer C, Chen D, Torneri A, Faes C, Wallinga J, et al. Estimating the generation interval for coronavirus disease (COVID-19) based on symptom onset data, March 2020. Euro Surveill. 2020; 25. doi: 10.2807/1560-7917.ES.2020.25.17.2000257 PMID: 32372755.

7. Zhou F, Yu T, Du R, Fan G, Liu Y, Liu Z, et al. Clinical course and risk factors for mortality of adult inpatients with COVID-19 in Wuhan, China: a retrospective cohort study. The Lancet. 2020; 395:1054–62. doi: 10.1016/S0140-6736(20)30566-3.

8. Sanche S, Lin YT, Xu C, Romero-Severson E, Hengartner N, Ke R. High Contagiousness and Rapid Spread of Severe Acute Respiratory Syndrome Coronavirus 2. Emerg Infect Dis. 2020; 26:1470–7. Epub 2020/06/21. doi: 10.3201/eid2607.200282 PMID: 32255761.

9. COVID-19 National Emergency Response Center. Coronavirus Disease-19: The First 7,755 Cases in the Republic of Korea. Osong Public Health Res Perspect. 2020; 11:85–90. doi: 10.24171/j.phrp.2020.11.2.05 PMID: 32257774.

10. Schuppert A, Theisen S, Fränkel P, Weber-Carstens S, Karagiannidis C. Bundesweites Belastungsmodell für Intensivstationen durch COVID-19. Med Klin Intensivmed Notfmed. 2021. Epub 2021/02/03. doi: 10.1007/s00063-021-00791-7 PMID: 33533980.

11. Tolksdorf K, Buda S, Schuler E, Wieler LH, Haas W. Eine höhere Letalität und lange Beatmungsdauer unterscheiden COVID-19 von schwer verlaufenden Atemwegsinfektionen in Grippewellen. 2020. Epub 2020/08/28. doi: 10.25646/7111.

12. Karagiannidis C, Mostert C, Hentschker C, Voshaar T, Malzahn J, Schillinger G, et al. Case characteristics, resource use, and outcomes of 10 021 patients with COVID-19 admitted to 920 German hospitals: an observational study. The Lancet Respiratory Medicine. 2020; 8:853–62. doi: 10.1016/S2213-2600(20)30316-7.

13. Verity R, Okell LC, Dorigatti I, Winskill P, Whittaker C, Imai N, et al. Estimates of the severity of coronavirus disease 2019: a model-based analysis. The Lancet Infectious Diseases. 2020; 20:669–77. doi: 10.1016/S1473-3099(20)30243-7.

14. Linton NM, Kobayashi T, Yang Y, Hayashi K, Akhmetzhanov AR, Jung S-M, et al. Incubation Period and Other Epidemiological Characteristics of 2019 Novel Coronavirus Infections with Right Truncation: A Statistical Analysis of Publicly Available Case Data. J Clin Med. 2020; 9. Epub 2020/02/17. doi: 10.3390/jcm9020538. PMID: 32079150.

15. UK Health Security Agency, editor. COVID-19 vaccine surveillance report. Week 14. 7 April 2022. Available from: https://assets.publishing.service.gov.uk/government/uploads/system/uploads/attachment_data/file/1067158/vaccine-surveillance-report-week-14.pdf.

16. UK Publich Health England, editor. COVID-19 vaccine surveillance report. Week 19. Available from: https://assets.publishing.service.gov.uk/government/uploads/system/uploads/attachment_data/file/986361/Vaccine_surveillance__report__week_19.pdf.

17. UK Health Security Agency. Vaccine surveillance report, week 26. Available from: https://assets.publishing.service.gov.uk/government/uploads/system/uploads/attachment_data/file/998411/Vaccine_surveillance_report_-_week_26.pdf.

18. Altarawneh HN, Chemaitelly H, Ayoub HH, Hasan MR, Coyle P, Yassine HM, et al. Protection of SARS-CoV-2 natural infection against reinfection with the Omicron BA.4 or BA.5 subvariants. ; 2022.

19. Altarawneh HN, Chemaitelly H, Ayoub H, Tang P, Hasan MR, Yassine HM, et al. Effect of prior infection, vaccination, and hybrid immunity against symptomatic BA.1 and BA.2 Omicron infections and severe COVID-19 in Qatar. ; 2022.

20. Chemaitelly H, Ayoub HH, Coyle P, Tang P, Yassine HM, Al-Khatib HA, et al. Protection of Omicron sub-lineage infection against reinfection with another Omicron sub-lineage. ; 2022.

21. Robert Koch Institut, editor. SARS-CoV-2 Sequenzdaten aus Deutschland. Available from: https://robert-koch-institut.github.io/SARS-CoV-2-Sequenzdaten_aus_Deutschland/.

22. Kheifetz Y, Kirsten H, Scholz M. On the Parametrization of Epidemiologic Models—Lessons from Modelling COVID-19 Epidemic. Viruses. 2022; 14. doi: 10.3390/v14071468 PMID: 35891447.

23. Kheifetz Y, Scholz M. Modeling individual time courses of thrombopoiesis during multi-cyclic chemotherapy. PLoS Comput Biol. 2019; 15:e1006775. Epub 2019/03/06. doi: 10.1371/journal.pcbi.1006775 PMID: 30840616.

24. Hooke R, Jeeves TA. `` Direct Search'' Solution of Numerical and Statistical Problems. J ACM. 1961; 8:212–29. doi: 10.1145/321062.321069.

25. Wu Y, Kang L, Guo Z, Liu J, Liu M, Liang W. Incubation Period of COVID-19 Caused by Unique SARS-CoV-2 Strains: A Systematic Review and Meta-analysis. JAMA Netw Open. 2022; 5:e2228008. Epub 2022/08/01. doi: 10.1001/jamanetworkopen.2022.28008 PMID: 35994285.

26. Li R, Pei S, Chen B, Song Y, Zhang T, Yang W, et al. Substantial undocumented infection facilitates the rapid dissemination of novel coronavirus (SARS-CoV-2). Science. 2020; 368:489–93. Epub 2020/03/16. doi: 10.1126/science.abb3221 PMID: 32179701.

27. Baden LR, El Sahly HM, Essink B, Kotloff K, Frey S, Novak R, et al. Efficacy and Safety of the mRNA-1273 SARS-CoV-2 Vaccine. N Engl J Med. 2021; 384:403–16. Epub 2020/12/30. doi: 10.1056/NEJMoa2035389 PMID: 33378609.

28. Lord JM. The effect of ageing of the immune system on vaccination responses. Hum Vaccin Immunother. 2013; 9:1364–7. Epub 2013/04/12. doi: 10.4161/hv.24696 PMID: 23584248.

29. Wang W, Balfe P, Eyre DW, Lumley SF, O'Donnell D, Warren F, et al. Time of Day of Vaccination Affects SARS-CoV-2 Antibody Responses in an Observational Study of Health Care Workers. J Biol Rhythms. 2022; 37:124–9. Epub 2021/12/04. doi: 10.1177/07487304211059315 PMID: 34866459.

30. Altarawneh HN, Chemaitelly H, Ayoub HH, Tang P, Hasan MR, Yassine HM, et al. Effects of Previous Infection and Vaccination on Symptomatic Omicron Infections. N Engl J Med. 2022; 387:21–34. Epub 2022/06/15. doi: 10.1056/NEJMoa2203965 PMID: 35704396.

31. https://www.rki.de/DE/Content/InfAZ/N/Neuartiges_Coronavirus/Projekte_RKI/SeBluCo_Zwischenbericht.html. Serologische Untersuchungen von Blutspenden auf Antikörper gegen SARS-CoV-2 (SeBluCo-Studie). 2021.

32. RKI, editor. Serologische Untersuchungen von Blutspenden auf Antikörper gegen SARS-CoV-2 (SeBluCo-Studie). Available from: https://www.rki.de/DE/Content/InfAZ/N/Neuartiges_Coronavirus/Projekte_RKI/SeBluCo_Zwischenbericht.html.

33. Schulze-Wundling K, Ottensmeyer PF, Meyer-Schlinkmann KM, Deckena M, Krüger S, Schlinkert S, et al. Immunity Against SARS-CoV-2 in the German Population. Dtsch Arztebl Int. 2023; 120:337–44. doi: 10.3238/arztebl.m2023.0072 PMID: 37155224.

34. Wachtler B, Neuhauser H, Haller S, Grabka MM, Zinn S, Schaade L, et al. The Risk of Infection with SARS-CoV-2 Among Healthcare Workers During the Pandemic. Dtsch Arztebl Int. 2021; 118:842–3. doi: 10.3238/arztebl.m2021.0376 PMID: 35264284.
